# Chiral Induced Spin Selectivity

**DOI:** 10.1021/acs.chemrev.3c00661

**Published:** 2024-02-16

**Authors:** Brian P. Bloom, Yossi Paltiel, Ron Naaman, David H. Waldeck

**Affiliations:** † Department of Chemistry, University of Pittsburgh, Pittsburgh, Pennsylvania 15260, United States; ‡ Applied Physics Department and Center for Nano-Science and Nano-Technology, The Hebrew University of Jerusalem, Jerusalem 9190401, Israel; § Department of Chemical and Biological Physics, Weizmann Institute, Rehovot 76100, Israel

## Abstract

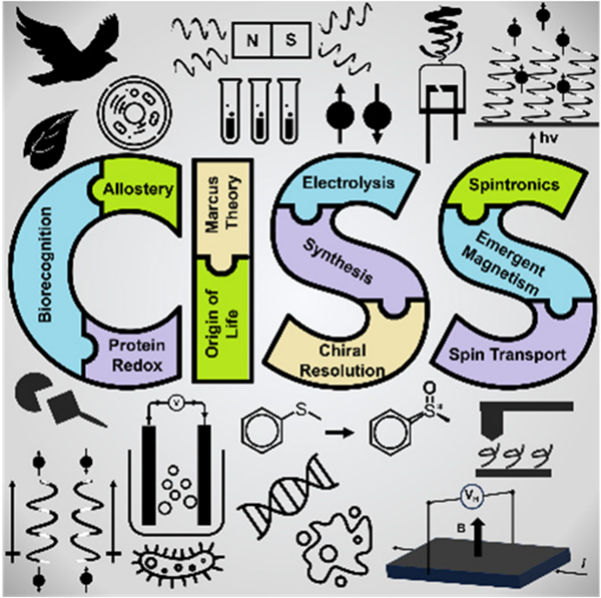

Since the initial landmark study on the chiral induced spin selectivity (CISS) effect in 1999, considerable experimental and theoretical efforts have been made to understand the physical underpinnings and mechanistic features of this interesting phenomenon. As first formulated, the CISS effect refers to the innate ability of chiral materials to act as spin filters for electron transport; however, more recent experiments demonstrate that displacement currents arising from charge polarization of chiral molecules lead to spin polarization without the need for net charge flow. With its identification of a fundamental connection between chiral symmetry and electron spin in molecules and materials, CISS promises profound and ubiquitous implications for existing technologies and new approaches to answering age old questions, such as the homochiral nature of life. This review begins with a discussion of the different methods for measuring CISS and then provides a comprehensive overview of molecules and materials known to exhibit CISS-based phenomena before proceeding to identify structure–property relations and to delineate the leading theoretical models for the CISS effect. Next, it identifies some implications of CISS in physics, chemistry, and biology. The discussion ends with a critical assessment of the CISS field and some comments on its future outlook.

## 1 Introduction

Since the time of Louis Pasteur, chiral symmetry and chiral molecules have intrigued chemists. Chiral molecules exist as stereoisomers, termed enantiomers, that are nonsuperimposable mirror-image structures of each other, like right and left hands. While the chemical formula and atomic connectivity of enantiomers are identical, their three-dimensional structure is not and gives rise to distinctive interactions with circularly polarized light. Chiral molecules that appear in organisms (lipids, carbohydrates, nucleic acids, and proteins) are homochiral, and even though the chemical behavior of enantiomers is often very similar, their bioactivity is not. Although conventional wisdom considers changes in chemical behavior to arise from differences in shape (lock and key mechanism of binding and enzymatic function), the reasons for homochirality and what might have guided Nature’s choice of one enantiomer over the other in biomolecules have long intrigued chemists.^1−3^ Even more generally, one might ask, “Why is chirality, as such, preserved so persistently throughout evolution?” or “What makes chiral symmetry so important to life?” Answers to these questions could involve the relationship between chirality and the electron spin, which manifests as the chiral induced spin-selectivity effect (CISS). CISS refers to the connection between chiral symmetry and electron spin in molecules and materials and it can manifest for electron transmission and for electron displacement currents.

**Figure 1:**
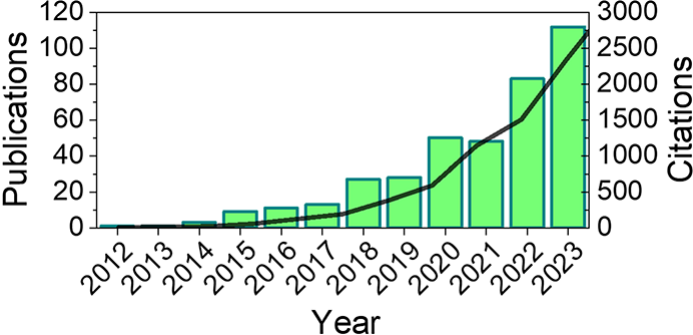
Number of publications, and the citations of those publications, using the phrase “chiral induced spin selectivity” or “chirality induced spin selectivity” from 2012 to 2022. The bars show the number of publications each year, and the solid curve shows the cumulative growth in citations. Data are from Clarivate Web of Science.

The idea that spin-polarized electrons scatter asymmetrically from chiral molecules was explored soon after the discovery of parity violation, i.e., the weak force breaks parity conservation, by Lee and Yang.^4^ However, studies with chiral molecules in the gas phase gave scattering asymmetries of *A* < 10^–4^, where *A* = (*I*_+_ – *I*_–_)/(*I*_+_ + *I*_–_) where *I*_+_ and *I*_–_ are the intensities of the electron beam with spin angular momentum parallel and antiparallel to the velocity. In 1999, we showed that the asymmetry in photoelectron scattering is >100-fold larger, ca. 0.1–0.2, when the electrons traverse through an ordered film of chiral molecules.^5^ Subsequent studies, using the same approach, reproduced these findings for other chiral molecular adlayers.^6−8^ In 2006, Wei et al. first showed that the phenomenon manifests for electron transport in electrochemical tunnel junctions,^9^ and since that time a large number of tunnel junction measurements and proximal probe studies have observed spin-dependent electron transport through chiral molecules and ultrathin chiral films.^10,11^ In 2011, Göhler et al. used Mott polarimetry to measure the photoelectron spin distributions through films of duplex DNA and found spin asymmetries as high as 60%.^12^ A perspective/mini-review of this early work in 2012 helped spark interest in this phenomenon, which is now called the CISS effect.^13^ Over the past decade, the number of publications using the term chiral-induced spin selectivity, and their corresponding citations has grown considerably year over year (see Figure 1).

This review aims to provide a more comprehensive description of the field and current understanding of CISS-related phenomena than that of reviews prior and comprises eight parts. In the next section we overview the different methodologies that have been used to measure the CISS effect, and then we follow with a section that summarizes the classes of molecules and materials shown to exhibit CISS. In Section 4, we identify general trends and inferences that can be drawn from particular experiments described in Sections 2 and 3, and in Section 5 we provide a brief assessment on the current status of theory, and its advances, since the review published in 2022.^14^ In Section 6, we describe some implications and applications of CISS for physics, chemistry, and biology. Lastly, we conclude with a critical assessment of the field (Section 7) and then offer some forward-looking sentiments (Section 8)

## 2 Methods for Measuring CISS

Direct experimental determinations of the CISS effect fall largely into two main measurement modalities: the observation of spin-dependent electron transport through chiral systems and the measurement of charge polarization-induced spin polarization of chiral systems. Transport/transmission measurements have been performed, both above the vacuum level (Section 2.1) and below the vacuum level (Section 2.2). While studies have attempted to calibrate the magnitude of the CISS-response across different measurement techniques,^15^ this process remains challenging because of differences in how the measurements are performed and how the CISS-response is quantified. In CISS studies the “spin polarization” has often been defined as the difference of measurables, for some process that selects for spin, divided by their sum. For example, the CISS literature often defines polarizations as normalized anisotropies in electron currents or charge transfer rate constants, and these quantities can be convoluted with the spin density of states. However, this treatment contrasts with classical definitions in which the spin polarization is formally given as the difference in populations for spin up and spin down electrons.^16,17^ Thus, care must be taken when comparing findings between different measurement techniques.

In addition to direct measurements, indirect probes for the CISS effect rely on the spin-selectivity of product formation in electrochemical reactions,^18^ or of charge polarization-induced spin polarization, and the complementary phenomenon of spin polarization-induced charge polarization, of chiral molecules and materials.^19,20^ In the latter case CISS has been shown to give rise to enantiospecific interactions, be they intermolecular or with ferromagnetic substrates,^21,22^ as well as spin-dependent charge delocalization.^19^ It is important to note that CISS is often a transient process,^23,24^ particularly as it pertains to enantiospecific interactions or measurements affected by decoherence, *vide infra*; therefore, measurement time scales are important for revealing spin selectivity.^25,26^ Below we summarize different measurement techniques that have been used to probe the CISS effect in chiral molecules and chiral materials.

### 2.1 Photoelectron Spectroscopy

Spin-resolved photoemission of electrons through chiral molecular films or ultrathin chiral materials is often considered the “gold standard” for quantifying the CISS effect because measurement of the electron spin population is not convoluted with charge displacement currents. These studies have provided insights into the importance of molecular helicity and length, as well as substrate spin–orbit coupling^12,15,27−29^ on the spin-dependent electron transmission. In cognate approaches, researchers have begun to explore CISS effects indirectly, e.g., through shifts in the substrate work function^30,31^ and through spin-dependent electron-induced chemical reactions.^32^

#### 2.1.1 Mott Polarimetry

**Figure 2:**
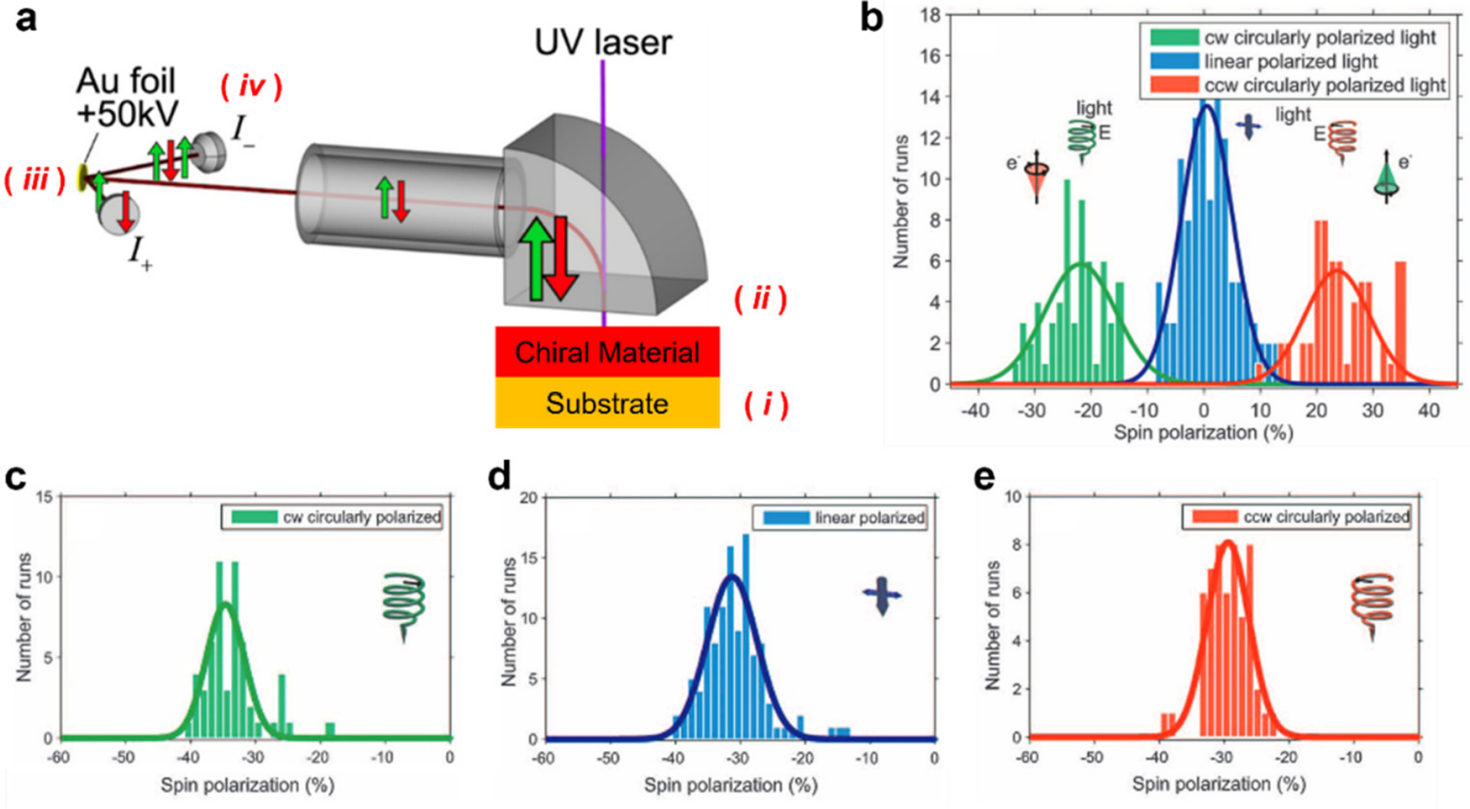
Representative schematic diagram (a) for the determination of CISS using Mott polarimetry measurements. First, photoelectrons in a substrate are excited (*i*) and then transmit through the chiral spin filter (*ii*), resulting in a net spin polarization. The photoelectrons are scattered on an Au foil target according to their spin (*iii*) and quantified at two independent detectors (*iv*). The schematic is reproduced with permission from ref (39). Copyright 2022 American Chemical Society. Panel b shows the photoelectron spin polarization from a bare Au(111) substrate excited with clockwise (green), linear (blue), and counterclockwise (red) polarized light. Panels c–e show the spin polarization for photoelectrons from an Au(111) surface that is coated with double-stranded DNA for clockwise, linear, and counterclockwise excitation, respectively. The data are adapted from ref (12) with permission. Copyright 2011 Science.

Mott polarimetry has historically been used for analyzing the magnetic characteristics of thin films^33−35^ and characterizing spin-polarized electron sources,^36^ among other applications.^37,38^ In this method electrons incident on a crystalline solid with large spin–orbit coupling (e.g., Au) scatter at different angles based on their spin orientation, and their angle dependent detection provides quantitative information about the spin population of the electrons. See ref (38) for a more detailed explanation and a historical perspective. For CISS studies, the typical process proceeds as follows: (*i*) electrons from a substrate, or a chiral material,^39^ are photoexcited, (*ii*) the photoelectrons transit through (from) a chiral layer where spin-filtering manifests, (*iii*) the photoelectrons are directed to a Mott polarimeter and spatially resolved according to their spin. The number of photoelectrons observed at the two detectors (gray spheres, *iv*) are used to determine the asymmetry or spin polarization, *P* (see Figure 2) through
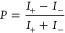
1where *I*_*+*_ and *I*_*–*_ correspond to the intensity of photoelectrons measured at the two different detectors.

By way of example, consider an Au substrate. Excitation of an Au(111) surface with clockwise and counterclockwise circularly polarized light produces spin-polarized photoelectrons with equal, but opposite, polarizations, whereas excitation with linearly polarized light does not give rise to a net spin polarization (see Figure 2b).^12^ Conversely, when the substrate is coated with double-stranded DNA, the polarizations measured for clockwise (Figure 2c), linear (Figure 2d), and counterclockwise polarized light excitation (Figure 2e) was negative, owing to the CISS effect. Similar studies have been carried out showing the spin selectivity of other oligonucleotides,^40^ oligopeptides,^15,40^ metal oxides,^39,41^ and helicenes.^28^ For a recent review of CISS studies using Mott polarimetry, see ref (29).

#### 2.1.2 Ultraviolet Photoelectron Spectroscopy

The determination of CISS using ultraviolet photoelectron spectroscopy (UPS) was first demonstrated by Weiss and co-workers for α-helical peptides immobilized on Co/Pt ferromagnetic substrates.^30^ Here, they measured changes in the photoelectron energies as a function of North and South magnetization of a Co/Pt substrate. A change in work function of ∼100 meV was observed and attributed to the spin-dependent exchange interactions occurring between the chiral molecules and the magnetized ferromagnetic substrate. Related studies on ferromagnetic substrates by Kelvin probe force microscopy have similar work function shifts.^19^ Because of the spin-selective electron delocalization of chiral molecules, the surface dipole moment is affected by the substrate magnetization. As a result, magnetization determines how much the adsorbates accept, or donate, charge density with the substrate and change the work function. Note that a spin-polarized detection scheme, such as Mott polarimetry, can also be used in tandem with UPS; work by Viswanatha et al. showed that the spin and momentum, both transverse and longitudinal, of the photoelectrons can be resolved for 3-methylcyclohexanone adsorbates on Cu(643) surfaces.^27^

### 2.2 Electron Transport

Since the first report in 2006,^9^ many studies have explored spin-polarized electron currents through tunnel junctions of various types. Notably, studies also show that electron transport through chiral semiconductors and chiral metals is spin-filtered. Thus, CISS does not originate from a particular subclass of conventional conductance mechanisms; however, it remains unclear if the mechanism underlying the spin selectivity is different for metallic conduction than it is for tunneling. To date, no correlation between reported spin polarizations and corresponding conductivities among different classes of materials has been reported.^42^

#### 2.2.1 Conductive Probe Atomic Force Microscopy

**Figure 3:**
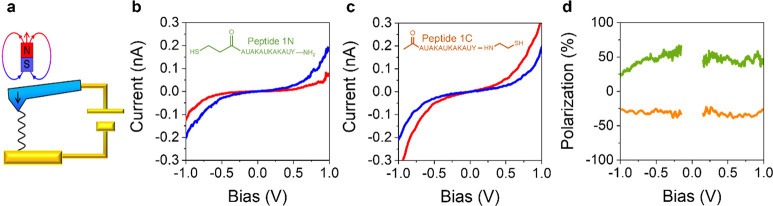
Panel a shows the experimental geometry used for a measurement of the current–voltage curves. Panel b shows current–voltage curves for peptide 1N with the linker on the N-terminus of the peptide. The blue curve corresponds to a South magnetized tip in which the electron transport is aligned parallel with its spin and the red curve corresponds to a North magnetized tip in which the electron transport is aligned antiparallel to its spin. Panel c shows current voltage curves for peptide 1C; in this case the South magnetized tip shows a lower current and the North magnetized tip shows the higher current. Panel d plots the percent spin polarization, as calculated from the data in panels b and c for peptide 1N (green, 44%) and peptide 1C (orange, −32%). The figure is adapted from ref (43) with permission. Copyright 2022 John Wiley and Sons.

Magnetic conductive probe-atomic force microscopy (mc-AFM) studies, in which a ferromagnetic electrode acts as a spin analyzer, are now available for a large range of organic and bio-organic molecules, hybrid organic–inorganic materials, and inorganic materials (see Section 3). mc-AFM measurements display distinctive characteristics (*vide infra*) and are being widely used. It is important to note, however, that the geometry in which the experiment is performed can determine the sign of the polarization; see ref (43) for a recent discussion on this topic. Figure 3 exemplifies these features for the case in which the AFM tip is magnetized, either North or South, relative to the molecule. The current voltage curve for the peptide 1N (Figure 3b) shows that the current is higher when the tip is magnetized to select for electrons with their spins oriented parallel to their velocity (blue) as compared to the case in which the electron spins are oriented antiparallel (red) to their velocity. That is, the magnitude of the current is higher when the electron spin direction and the electron velocity are aligned parallel. It is common to define a percent polarization as
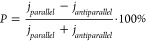
2and this is plotted in Figure 3d for the peptide 1N (green). The same measurement on peptide 1C (linker attached to the C-terminus of the peptide rather than to the N-terminus) displays an opposite behavior. That is, the current is higher for the case where the spin is aligned antiparallel to the electron velocity (see Figure 3c). An analogous dependence on the peptide’s attachment to the surface was reported from photoemission studies.^8,44^

The experimental mc-AFM studies have caused intensive discussion because they display behavior that differs from those commonly expected for magnetoresistance devices used in spintronics.^45^ Those devices are based on two ferromagnetic electrodes comprising a “hard” magnet (high coercivity) and a “soft” magnet, with an insulating metal oxide layer, typically tens of nanometers thick, between them. In common magnetoresistance devices, the current behaves as if it flows through a diode, namely one spin current is dominant under positive voltage bias and the other under negative bias. This is not the case for CISS-based devices where the same spin-current is dominant, independent of the voltage sign. In addition, CISS-based devices often display spin polarizations that are higher than what is expected for the magnetic layer acting as the analyzer polarizer, which implies that the chiral molecular junction must be nonlinear. In fact, the current in CISS-based junctions often depend nonlinearly on the voltage, see Section 3.1.1 and the discussion below.

The current–voltage data in the mc-AFM measurements can be understood by considering a model in which the applied voltage polarizes the chiral system and this charge polarization is accompanied by spin polarization (see ref (46)). The model considers a chiral molecular film located between two leads, one of them a ferromagnet, and assumes that charge polarization of the chiral molecules by the applied voltage causes spin polarization. The positive pole of the chiral molecules is associated with one spin and the negative pole is associated with the opposite spin in an enantiospecific manner, i.e., depends on the handedness. Hence, electrons that have to penetrate into the chiral system from the ferromagnetic electrode confront a spin-dependent barrier whose magnitude is proportional to the charge at the pole times the spin-exchange interaction. By assuming that the charge at the pole is about 10% of an electron charge and that the magnitude of the spin-exchange interaction is on the order of 1 eV, one finds the difference in barrier height for the two spins to be ∼100 meV. Indeed, experiments indicate that the difference in injection barrier for the two spins is of this order of magnitude, e.g., see ref (10). Such a barrier explains the very high spin selectivity at room temperature. Other works have proposed “spinterface” models to explain these experimental signatures in a more quantitative manner (see Section 5.1).

#### 2.2.2 Scanning Tunneling Microscopy Methods

Diez-Perez and co-workers^11^ used scanning tunneling microscopy (STM) break-junction measurements to show that CISS manifests in single molecule junctions, i.e., the spin-filtering does not depend on having a chiral film, but can manifest at the single molecule level. In this work, they trapped individual peptide molecules between a magnetized STM Ni tip and an Au electrode and measured the current. They found that the molecular conductance depends on the magnetization state of the STM tip and on the enantiomeric form of the peptide. Note that these measurements did not display a perfect antisymmetry, and it was necessary to invoke a spin-to-charge voltage (or “spinterface” effect, see Section 5.1) to fully explain the data. More recently, CISS studies at the single molecule level have been performed by Bürgler and co-workers, who used spin-polarized STM to examine the enantioselective adsorption of chiral molecules on magnetic surfaces,^47,48^ and by Ortuño et al., who have developed a chiral oligo(phenylene)ethynylene based molecular tunnel junction and used theoretical calculations to predict spin polarizations of 20% to 40%.^49^ Collectively, these experiments demonstrate that CISS manifests at the single molecule level.

#### 2.2.3 Magnetoresistance and Spin Valve Studies

The mc-AFM method described in Section 2.2.1 can be viewed as a spin valve, in which the magnetic probe tip, or the substrate, acts as the ferromagnetic contact. A vertical magnetic spin valve device comprises a chiral film that is contacted on one side to a normal metal electrode and on the other side to a magnetic electrode, whose magnetization direction can be changed by an applied external magnetic field. In this device, a magnetoresistance is obtained from measurements of the current–voltage response through the top and bottom contacts as a function of an applied magnetic field and probe how the chiral film affects the magnetoresistance. This configuration was first used to measure spin-selective charge transfer through a self-assembled monolayer of polyalanine with a magnetized Ni layer,^50^ and has since been used to evaluate the magnitude of the CISS effect with other chiral systems.^51^ Unlike standard spintronic devices, in this configuration only one magnetic layer is needed for a CISS-based device because the chiral axis direction of the insulating layer determines the spin direction that is analyzed. In a four-probe setup it is possible to measure the magnetoresistance without the contribution of the contact resistances. In this configuration, larger area devices are used and smaller magnetoresistance values are found; see ref (52) for further explanation and Figure 9 for a representative example. Note that more elaborate spin-valve structures have also been used for studying CISS.^53,54^

#### 2.2.4 Electrochemical Tunnel Junctions

The determination of CISS in a tunnel junction configuration was first shown using electrochemical methods on porphyrin terminated chiral molecular scaffolds, l-Cys-(pro4(2S4S))_4_-Porph and d-Cys-(pro4(2R4R))_4_-Porph, immobilized on gold electrodes.^9^ Here, excitation of the porphyrin with circularly polarized light (left vs right) created a spin-polarized population of porphyrin excited states and the subsequent photocurrent was measured. Interestingly, a spin polarization (ca. 0.5%) in the photocurrent was observed with excitation polarization and was found to depend on the handedness of the molecular scaffold. The results were rationalized as spin-dependent processes affecting the electronic coupling. Similar results have been shown for chiral oligopeptide SAMs with tethered CdSe quantum dots on ferromagnetic electrodes placed in contact with a ferri-/ferrocyanide redox couple; however, here the polarizations were determined by changing an applied magnetic field on the electrode.^55^ Upon excitation, polarizations as high as 30% were reported at the redox potentials of the ferri-/ferrocyanide and the findings were corroborated by steady-state fluorescence measurements that monitored the asymmetry in quenching associated with electron transport to the electrode. Electrochemical methods have also been used to measure spin polarization in the dark; Kettner et al. observed changes in the oxidation and reduction currents for ferri-/ferrocyanide solutions, using magnetized electrodes coated with oligopeptide SAMs.^15^ The larger current response was found when the spins were aligned antiparallel to their momentum, in agreement with the conclusions drawn from photoemission measurements for the same oligopeptide assemblies.

Researchers have quantified changes in the charge transfer rate, *k*^0^, of redox species attached to chiral monolayers. For instance, ferrocene-oligopeptide composites immobilized on gold showed an asymmetry in the charge transfer rate for reduction and oxidation that depends on the handedness of the oligopeptide, e.g., for l-oligopeptides the *k*^0^ for reduction was faster than the *k*^0^ for oxidation and for d-oligopeptides the *k*^0^ for reduction was slower than the *k*^0^ for oxidation.^56^ This behavior was attributed to an induced magnetization associated with the oligopeptide assembly, similar to that shown in other works,^57^ and the CISS-mediated transport properties of the oligopeptides. Experiments have also been performed on magnetized ferromagnetic electrodes, so as to exclude spontaneous magnetization effects.^58^ In these studies cytochrome *c* was immobilized on Cys-Ala-Glu tripeptide monolayers and electron transfer from the cytochrome *c*’s heme unit to the electrode was measured. For tripeptide SAMs in which each of the substituents was levorotatory (LLL), a North applied magnetic field led to a faster rate constant than a South magnetic field, whereas tripeptide SAMs comprising all dextrorotatory substituents (DDD) resulted in the opposite dependence, i.e., a South magnetic field led to a faster rate constant than North magnetic field. For SAMs with a heterochiral structure, e.g., LDL, the electron transfer rates for North and South applied magnetic fields were the same.

The spin-specific change in charge transport through chiral molecules in electrochemical tunnel junctions, be that through monitoring changes in current or charge transfer rate, likely arise from the same phenomenon: spin-dependent changes in resistance for charge transport. This supposition is supported by recent impedance measurements made on DNA coated ferromagnetic electrodes, in which an equivalent circuit model analysis is used to extract the charge transfer resistance as a function of applied magnetic field.^59−61^ For the DNA assemblies, deviations in charge transfer resistance with magnetization orientation are observed, owing to the CISS effect; however, in achiral systems the charge transfer resistance is unaffected by the magnetic field orientation.

#### 2.2.5 Hanle Rotation

The Hanle effect can probe the spin polarization of carriers in a semiconductor by measuring their spin precession and dephasing as they propagate through a transverse magnetic field.^62,63^ In addition to determining spin lifetimes, Hanle effect measurements also report on pure spin transport and on spin accumulation, which gradually reduces to zero with increasing magnetic field strength.^64,65^ In traditional electrical Hanle measurements a ferromagnet is used to generate a spin-polarized current in a transport channel that is probed through spin accumulation on a semiconductor. Recently, Xiong and co-workers generated spin-polarized current by injecting electrons from an Au electrode, which was coated with an α-helical polyalanine film, into a transport channel and detected the spin accumulation at a GaAs electrode (Au/l-polyaniline/Si:GaAs junctions).^66^ They observed universal temperature and bias current dependences for the spin-polarized carriers. These results provide further evidence that CISS-based spin polarization can be detected without the use of a ferromagnet.

### 2.3 Charge Polarization and Spin Polarization Methods

The methods in Section 2.2 rely on steady-state or periodic charge currents; however, this need not hold for observing spin polarization in chiral molecules. Recent studies show that it is enough to charge polarize chiral materials transiently to generate a spin polarization, and that the complementary response in which a magnetization induces a charge polarization can manifest.

#### 2.3.1 Spin-Dependent Polarization in Hall Voltage

Surface magnetizations, induced by the CISS response of a chiral film, have been investigated using the Hall and anomalous Hall effects.^20,67^ Hall effect devices^68^ are widely used for continuous monitoring of spin-induced magnetization,^69^ and they commonly have one of two configurations: a standard Hall bar configuration and a van der Pauw square configuration.^70^ It is important to note that in shallow two-dimensional electron gas (2DEG) devices the surface spins interact strongly with the 2DEG through Ruderman–Kittel–Kasuya–Yosida (RKKY) interactions.^71^ When spin-polarized electrons are injected into III–V heterostructures (such as AlGaN/GaN or AlGaAs/GaAs) that contain a 2DEG layer, the semiconductor becomes magnetized, even at room temperature, with a magnetization direction that depends on the direction of the polarization of the injected spins.^72^ For dry measurements, a shallow GaAs 2DEG is mostly utilized,^67^ while for liquid solutions GaN-based 2DEG are most used.^72^ The latter can also be used to monitor the spin dependence of electrochemical processes.

**Figure 4:**
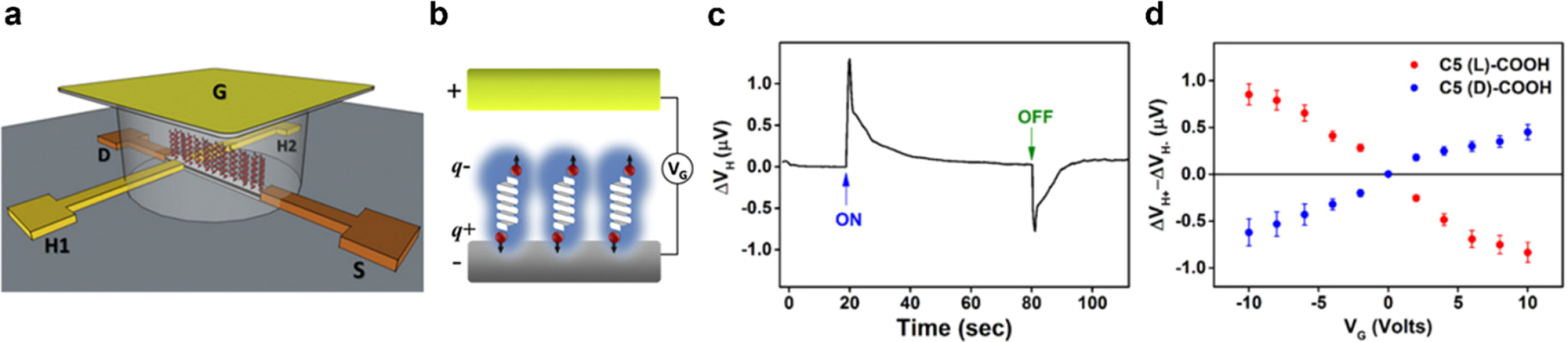
Panels a and b show a representative schematic diagram of a Hall device passivated with chiral oligopeptides. Panel c shows that upon charge polarization of the oligopeptides, a transient Hall voltage is generated. Panel d shows the dependence of the Hall voltage on the magnitude and sign of the gate voltage and the handedness of the oligopeptides. The figure is adapted from ref (20) with permission.

In 2017, Kumar et al. used a Hall bar device to show that an applied voltage acting on a chiral oligopeptide film generates a magnetization at the interface between the monolayer film and the Hall bar surface, even though no net current flows.^20^Figure 4a,b shows a schematic diagram of their experimental measurement design. They constructed a Hall bar circuit, which was buried under a few nm thick film of GaN and then coated with a self-assembled monolayer film. The application of a voltage between the bottom working electrode and the counter-electrode (G) creates a displacement current that charge polarizes the chiral molecular film and generates a transient Hall voltage signal (Figure 4c), which then decays. Upon release of the applied voltage, the film discharges and generates an opposite Hall voltage because of the opposite direction of current flow. Figure 4d shows that the signal increases with the magnitude of the applied voltage, which increases the charging current, and that it is enantiospecific, i.e., a film of l-oligopeptide has a response opposite in sign to that of a film of d-oligopeptide. Note that no magnetic materials and no external magnetic fields are present in this experiment; the chiral charge polarization of the molecules gives rise to a spin polarization at the bottom of the film that manifests as a magnetization that acts on the charge carriers moving in the source drain channel.

The Hall circuit design can be incorporated into a working electrode, which can be used to probe spin-selective charge transfer and charge displacement processes. The electrochemical cell used in the above experiment was constructed to not display any Faradaic current, so that the oligopeptide-coated electrode surface would closely approximate an ideally polarizable electrode. If instead, one constructs an electrochemical cell with a redox couple, then Faradaic current can flow and the working electrode, with its embedded Hall device, allows one to monitor the spin dependence of redox reactions in addition to the charge currents that are traditionally measured.^72^ By using a working electrode that possesses an embedded Hall probe, one can perform “3D spin electrochemistry”,^73^ i.e., measure the current, voltage, and spin simultaneously for redox reactions.^74^

Most of the Hall signal induced by chiral molecule adsorption on metals and semiconductors seems to arise from the anomalous Hall effect.^75^ This was verified by experimentally verifying the relation between the longitudinal and Hall resistance as a function of temperature.^76^

#### 2.3.2 Emergent Magnetic Properties and Magnetic Force Microscopy

**Figure 5:**
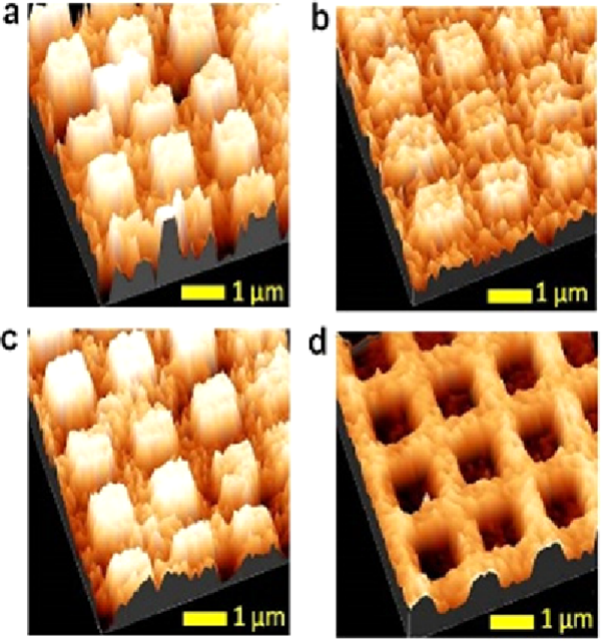
Topography and magnetic force microscopy phase images are shown for a molecular-induced magnetization orientation. The top row shows AFM topography images of SAMs of l-polylalanine (a) and d-polyalanine (b) adsorbed on Co thin ferromagnetic layers with a 5 nm Au overlayer, and the bottom row shows their corresponding magnetic AFM magnetic phase images (l-polylalanine (c) and d-polylalanine (d)). Adsorption of oligopeptides induce a magnetization, and the direction of the magnetization is controlled by the enantiomeric form of the molecules. The figure is adapted from ref (57) with permission (http://creativecommons.org/licenses/by/4.0/).

The spin polarization, which is generated by the charge redistribution in chiral molecules, can be stabilized in a ferromagnetic film. Figure 5 shows magnetic force microscopy images of lithographed surfaces in which chiral molecules, by virtue of their charge-polarization induced spin-polarization, imprint a magnetization onto a ferromagnet.^57^Figure 5a,b shows topographical images of a patterned surface on which l- and d-polyalanine monolayers are adsorbed, and the corresponding magnetic force microscopy images in Figure 5c,d show opposite magnetization directions. These findings establish that as chiral molecules and ferromagnetic layers come into contact, the spin-polarized current exchange between the chiral layer and the ferromagnet is very efficient in polarizing the spins of the ferromagnet (Figure 5).^50^ In this case, about 10^13^ electrons per cm^2^ are sufficient to induce magnetization reversal. The direction of the magnetization depends on the handedness of the adsorbed chiral molecules, i.e., it is enantiospecific. In contrast, the current density required for the spin-transfer torque in modern magnetoresistive random access memory is 10^6^A cm^2^, or about 10^25^ electrons cm^2^/s, a trillion times higher. Note that the inverse effect, enantiospecific interaction of chiral molecules with a magnetized substrate, can be used to separate chiral molecules^21^ (see Section 6.2).

#### 2.3.3 Spin Exchange Microscopy

The enantiospecific interaction between chiral molecules and ferromagnetic surfaces enables one to perform locally resolved magnetic imaging by adsorbing chiral molecules on an AFM tip.^78^ This technique is based on short-range spin-exchange interactions that can be scaled down to atomic resolution and only require a conventional AFM tip functionalized with a chiral molecule. A direct measurement of the force and tip displacement for the interaction between a ferromagnetic substrate and chiral molecules provides energy for the interaction, and the difference in energy for the two magnetization directions (North versus South) of the ferromagnet allows one to determine the difference in exchange energies. The mean pulling energy showed a difference of 150 meV for sample magnetizations of North and South along the sample normal.

**Figure 6:**
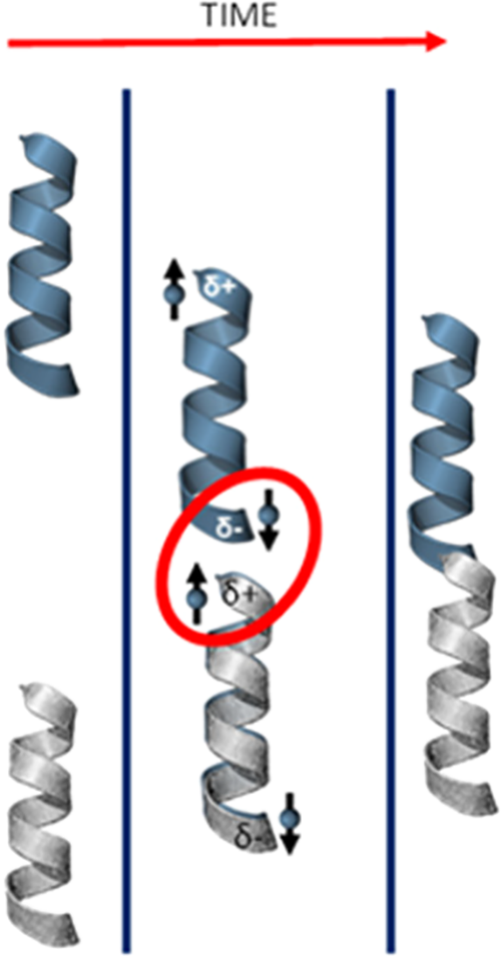
The image shows the effect of spin-dependent charge reorganization interactions between two chiral molecules. From left to right: The two chiral reactant molecules are represented by helices and are noninteracting at a very large distance. As the chiral molecules approach each other dispersion forces generate induced dipoles on each molecule, which in turn are accompanied by a spin polarization. The two chiral molecules react to give a product with an energy that depends on whether the spin polarizations on the molecules are aligned antiparallel or parallel.

To illustrate the phenomenon, consider the interaction between two helical molecules. When two chiral molecules of the same handedness interact, the charge polarization is accompanied by a spin polarization acting in the same direction, e.g., pointing outward along the helix axis (see Figure 6), and the exchange interaction between the molecules’ excess spin densities in the overlap region is characterized by two spin polarizations aligned antiparallel. In contrast, the spin polarizations of two interacting molecules of opposite chirality would be aligned parallel. The difference in these spin arrangements generates a change in exchange energies.^20^ Note that the spin polarization manifests even when the two molecules are each closed shell; while they remain singlet states globally, their electron clouds can locally display spin imbalances.

#### 2.3.4 Kelvin Probe Force Microscopy

**Figure 7:**
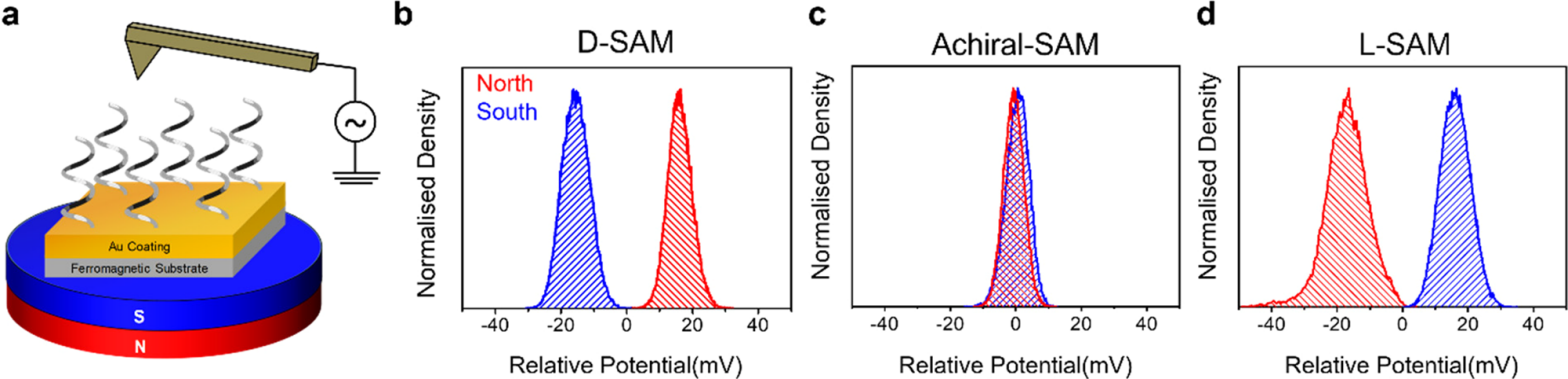
Panel a shows a schematic diagram for the Kelvin probe measurement. Panels b–d show changes in the measured contact potential difference with North (red) and South (blue) magnetizations for d-oligopeptide SAMs, achiral SAMs, and l-oligopeptide SAMs, respectively on ferromagnetic electrodes. The figure is adapted from ref (19) with permission. Copyright 2020 American Chemical Society.

Probing the surface potential that is induced by spin transfer can be achieved using Kelvin probe force microscopy (KPFM). The basic Kelvin probe measurement consists of a metallic probe electrode that is placed near the sample surface to form a capacitor.^79^ Then, the distance between the probe electrode and the sample surface is changed periodically to generate a frequency dependent capacitance. Thus, an AC voltage is created across the gap, and it is proportional to the voltage difference between the probe electrode and the sample. Rather than record the AC voltage directly, it is common to apply a DC voltage, referred to as the contact potential difference (CPD), to null the response. To measure the CISS-induced spin wave function changes, the CPD can be measured when altering the adsorbate’s enantiomeric form and the surface magnetization (see Figure 7). Kelvin probe measurements on ferromagnetic thin film electrodes coated with self-assembled monolayers of chiral molecules reveal that the electron penetration from the metal electrode into the chiral molecules depends on the ferromagnet’s magnetization direction and the molecules’ chirality. Figure 7b–d shows the changes in the measured CPD with North (red) and South (blue) magnetizations for d-oligopeptide SAMs, achiral SAMs, and l-oligopeptide SAMs, respectively.^19^ Electrostatic potential differences as large as 100 mV are observed and arise from the applied oscillating electric field, which drives spin-dependent charge penetration from the ferromagnetic substrate to the chiral molecules. The large potential changes (>*kT* at room temperature) imply that this phenomenon is important for spin transport in chiral spintronic devices and for magneto-electrochemistry of chiral molecules.

### 2.4 Other Techniques

While studies of the CISS effect in chiral materials are most prevalent using the techniques discussed above, a number of other strategies have been demonstrated and are being developed. Here, the manifestation of a CISS-response is akin to that described previously, in that it arises from a spin-dependent response in the material that depends on the chirality.

#### 2.4.1 Fluorescence

Fluorescence spectroscopy can provide detailed information about relaxation and/or charge and energy transfer processes that take place following light absorption. Thus, if energy transduction is affected by spin selectivity in a chiral system, then the photoluminescence of a chromophore can report on the spin-dependence for the transduction. For instance, researchers showed that the photoluminescence of nanoparticles tethered to magnetized ferromagnetic substrates through chiral oligopeptides changes with the orientation of an external magnetic field.^55^ Here, hole transfer, and hence photoluminescence quenching, from the nanoparticle to the substrate depended sensitively on the match, or mismatch, between the spin selectivity of the oligopeptide and the magnetization orientation of the substrate. Similar measurements have been made in which spin-dependent electron transfer^80^ and energy transfer^81^ processes are responsible for controlling the chromophore’s photoluminescence intensity with substrate magnetization.

In addition to steady-state fluorescence, time-resolved measurements can provide information about the importance of spin on charge transfer kinetics. Such behavior was demonstrated by studies of donor-bridge-acceptor nanoparticle systems, in which the acceptor was made chiral.^82^ Here, excitation of the donor nanoparticle with clockwise and counterclockwise circularly polarized light, thus yielding spin-polarized excitation of the donor, resulted in large differences in charge transfer rates to the acceptor because of the CISS effect. Note, however, that the efficacy of these measurements relied on several factors: (*i*) the system required a principal excitation axis to define the electron spin orientation relative to that of the transport trajectory, and (*ii*) the time scale for electron transfer had to be shorter than the decoherence of the spin.

#### 2.4.2 Resonance Spectroscopies

To date, only a handful of spectroscopy methods have been applied to the study of CISS; however, they are likely to prove very important in future studies, because they can provide incisive information about CISS when the chiral system is weakly coupled to its surroundings.

##### 2.4.2.1 Cross-Polarization NMR

Although “conventional wisdom” holds that nuclear magnetic resonance (NMR) spectroscopy is not sensitive to a molecule’s chirality unless it is perturbed by a chiral bias of some sort, this assumption is overly simplistic. For example, Buckingham has shown that NMR methods that sense odd parity magnetoelectric coupling terms should be able to directly probe chirality.^83,84^ In other works, Ugalde and co-workers used CP-NMR to measure the solid-state NMR spectra of ^15^N nuclei for different enantiomers of amino acids, and found a systematic and significant increase in the signal levels for the d-isomer over that for the l-isomer, even though the chemical shifts are identical.^85,86^ The CP-NMR experiment transfers polarization from the majority nuclear spins (protons in this case) to the dilute minority spins (^15^N nuclei in this case) and the efficiency of this process is enantiospecific, giving rise to higher signal intensities for the d-isomer in their spectrometer. This finding implies that the coupling, which leads to the polarization transfer, is enantiospecific and they propose a mechanism based on CISS to rationalize their findings. Experiments of this sort are important for studying fundamental features of CISS, because they do not have the complications associated with molecule–substrate couplings; rather, they probe the interaction between nuclei in the amino acid molecules, via the molecule’s CISS-based electronic response.

##### 2.4.2.2 Time-Resolved EPR Studies

Electron paramagnetic resonance (EPR), or electron spin resonance (ESR), spectroscopy can provide a direct probe of spin polarization. Its use in CISS studies was first reported in 2020 by Ansermet and co-workers to probe the spin polarization of paramagnetic radicals that are produced by electrochemical reduction at a chiral electrode (i.e., Au coated with an oligopeptide).^87^ Since that initial report, a number of other research groups have actively pursued experimental and theoretical studies into identifying CISS signatures in EPR spectra and proposed photochemical mechanisms to enhance them.^26,88−90^ Recently, Wasielewski and co-workers have demonstrated that photoinduced electron transfer in a donor-chiral bridge-acceptor molecule gives rise to electron spin polarization in the biradical product, ca. 50%.^91^

#### 2.4.3 Magnetometry and Magneto-optical Methods

The accumulation of spin polarization at interfaces, or even self-contained in chiral materials, has led to the use of magnetic-based detection schemes for monitoring the CISS effect. Indeed, such behavior is responsible for the response in Hall devices (Section 2.3.1), magnetic force microscopy (Section 2.3.2), and spin-exchange microscopy (Section 2.3.3) measurements; however, other methods for detection have also been employed. For instance, superconducting quantum interference device (SQUID) magnetometry has been used to measure the effect of chiral molecules on the magnetic properties of materials; studies show that superparamagnetic iron oxide nanoparticles adsorbed on chiral self-assembled monolayers become ferromagnetic^92^ and conventional superconductors may exhibit topological superconductivity when interfaced with chiral molecules.^93^

Another technique, which has been used to probe the CISS-effect in chiral materials, is magnetic circular dichroism. Here, a magnetic field is oriented parallel or antiparallel to the direction of light and the differential adsorption of left and right circularly polarized light is measured.^94^ In a typical experiment, the orientation of the external magnetic field determines the sign of the optical activity. Conversely, when the CISS effect is stronger than the effect imposed by the external magnetic field, a change in magnetic field orientation does not change the sign of the optical activity. Such behavior has now been observed for chiral mesostructured BiOBr and α-Fe_2_O_3_.^95,96^

**Figure 8:**
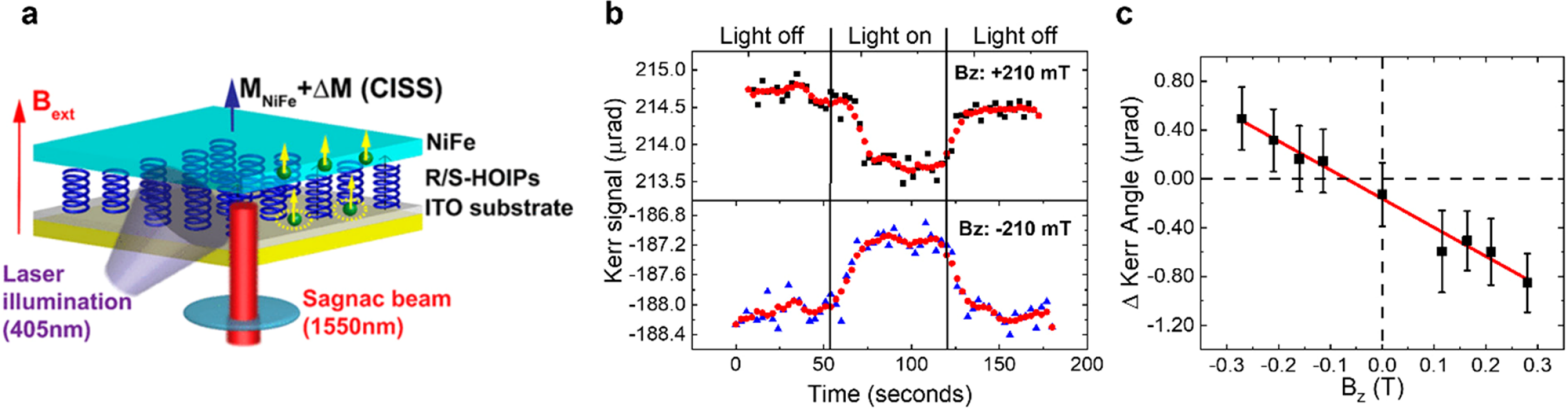
Panel a shows an experimental schematic for magneto-optic Kerr effect measurements on chiral perovskite thin films. Panel b shows magneto-optic Kerr rotation measurements on S-hybrid organic–inorganic perovskites, under positive (top) and negative (bottom) out-of-plane external magnetic fields. The red line is an adjacent average smoothing of the data. Panel c shows the change in photoinduced Kerr response as a function of the external magnetic field strength. The red line is a linear fit to the data. The figure is adapted from ref (97) with permission. Copyright 2020 American Chemical Society.

Spin transport from chiral materials to adjacent ferromagnetic layers has been probed by magneto-optic Kerr effect (MOKE) measurements in which a change in the magnetization of a magnetic material is monitored through changes in the reflection of polarized light off its surface. Figure 8a shows a schematic for MOKE measurements. Here, linearly polarized excitation of a chiral perovskite film creates a photoinduced spin current that magnetizes an adjacent ferromagnetic layer.^97^ The Kerr angle, reflecting the change in magnetization of the ferromagnetic layer, depends on the perovskite’s enantiomorph, whereas achiral perovskites show no response. Figure 8b shows the change in MOKE response upon photoexcitation of the perovskites, and Figure 8c plots the change in Kerr angle with applied magnetic field. Experiments have shown that a change in Kerr response can occur upon photoexcitation or thermal activation of chiral layers because of CISS-mediated transport.^97,98^

Note that other spectroscopic methods, such as optically detected magnetic resonance via nitrogen vacancies in diamond, are also being used to measure magnetization effects for CISS studies, e.g., reorientation of ferromagnetic layers upon adsorption of chiral molecules.^99^

#### 2.4.4 Spin Seebeck Effect

In magnetic materials, spin currents can arise from temperature gradients by the conventional spin Seebeck effect.^100^ Recently, Sun and co-workers used a temperature gradient to generate a spin selectivity effect in chiral materials without any ferromagnetic layer, which they call the CPASS (chiral-phonon-activated spin Seebeck) effect.^98^ In this case the chiral phonon–electron coupling generates a spin current because of the conservation of angular momentum, i.e., the chiral phonons transfer angular momentum to the electron spin angular momentum. CPASS provides a unique and incisive probe for examining the importance of electron–phonon coupling for the CISS effect. CPASS could also be used to distinguish between coherent and incoherent chiral pumping of spin waves in thin magnetic films.^101^

## 3 Materials and Molecules Exhibiting CISS

Early experiments on CISS have examined spin-dependent electron transport and electron polarization with organic molecules, for which the structure and organization of their assemblies can be manipulated. More recent studies have shown that CISS manifests for a wide array of molecular, supramolecular, and materials types. CISS effects have been reported for insulating, semiconducting, and metallic chiral solids; chiral quantum dots, chiral 2D layered materials, and chiral polymers, including biopolymers, and their assemblies. Here we overview these studies and identify key aspects about CISS, which they have revealed. Note that, a comparison across material types is discussed in Section 4.

### 3.1 Molecules and Macromolecules

#### 3.1.1 DNA and Oligopeptides

DNA (see refs (7, 10, 12, 31, 52, 59−61, 80, 102−111)) and α-helical oligopeptides (see refs (11, 15, 19−21, 30, 43, 44, 50, 52, 54−57, 67, 69, 72, 74, 77, 92, 99, 104, 112−126)) have been widely used to explore the CISS effect and its connection with molecular properties. Having been investigated by spin-dependent photoemission, transport, electrochemical, and spin-dependent polarization experiments, they comprise testbed systems for comparisons between methods. These studies have provided a number of key insights into CISS properties.

##### Polarization, P, as a Metric

For both families of molecules spin polarization exceeding 60% was found, when the polarization, *P*, is defined as  with *j*_+_ and *j*_–_ referring to the charge current measured when the magnetic North pole is pointing toward the adsorbed molecules or away from them, respectively; and these magnitudes compare well to those observed by spin-polarized photoemission studies.^15^

##### Length Dependence

Over the size ranges studied (<20 nm for DNA and shorter for oligopeptides), the *P* increases linearly with the length. It is found that the α-helices of oligopeptides are about a factor of 5× better spin filters than DNA, on a per nanometer basis.^104^ The length dependence of the spin polarization results from the conduction of the favored spin decaying more slowly as a function of length than does the unfavored spin.

##### Voltage Dependence

In the conduction studies performed with the mc-AFM method, it was established that spin-dependent conduction displays a power law dependence on the voltage, with the power *d* being greater than one, and that a different voltage threshold for conduction exists for each of the spin polarizations. The different thresholds imply that spin flipping during the conduction is not significant, i.e., a mixed spin distribution would not generate different voltage thresholds.^104,127^

##### Point Chirality versus Helical Chirality

Helical chirality appears to give rise to stronger spin filtering than the point chirality of individual stereocenters. Electrochemistry-based measurements show that folding DNA duplexes, comprising the same nucleobase sequences, into right-handed or left-handed helices controls the sign of the spin-filtering, implying that the helical twist of the duplex DNA dominates over the point chirality of the sugars.^102^ This observation is consistent with Mott polarimetry photoelectron studies using disordered films of single-stranded DNA and oligopeptides that display poor spin-filtering.^12^

##### Dipole Orientation Effect

Both photoemission^8,44^ and mc-AFM measurements^43^ show that the sign of the spin-polarized current changes with the orientation of the molecule on the electrode. That is, placing an oligopeptide on a metal substrate by its carbon end gives a different sign for the spin polarization than binding it to the electrode by its nitrogen end.

##### Circular-Dichroism (CD) is a Predictor

Studies on oligopeptides of the same length but different CD strengths (lowest energy) show that the spin filtering increases as the CD strength increases.^43^ This claim is corroborated by studies on chiral quantum dots^82^ (see Section 3.2.2) and supramolecular structures^128,129^ (see Section 3.2.1).

#### 3.1.2 Helicenes

Although helicenes do not contain carbon stereocenters, they possess axial chirality. CISS manifests in enantiospecific adsorption of helicenes to magnetized surfaces, in spin-filtered electron transmission via photoelectron spectroscopy, and in conduction experiments through monolayer films of helicenes.^28,47,48,130−134^ Although several classes of helicenes have been investigated, no reports have drawn a clear correlation between the structure of a helicene and its spin-filtering power. Photoemission studies indicate some effect of the substrate on the spin polarization; however, no simple correlation was found between the spin–orbit coupling of the substrate and the size of the spin polarization in the CISS effect.^28^ As a caveat, one must appreciate that different binding groups are used for attaching molecules to the different substrates and this can lead to different charge distributions at the interface, hence work functions. Thus, the exact role of the substrate remains an open issue and may require careful studies to reach firm conclusions.

#### 3.1.3 Proteins

Experiments find that both redox proteins, as well as other proteins, display spin-polarized electron transport; including photosystem I, cytochromes, azurin, and multiheme electron transfer conduits, among others.^22,58,115,135−142^ These observations are consistent with electron transfer via peptidic pathways in proteins. Beyond these pioneering demonstrations, systematic studies have examined the temperature dependence of spin filtering, the importance of homochirality in electron transfer, and the role of CISS in allostery.

##### Temperature Dependence

Temperature-dependent conductance measurements show that the spin-filtering decreases with decreasing temperature, even in cases where the overall conductance remains approximately constant. These studies imply that spin-dependent transport is activated, suggesting the importance of phonons for CISS to manifest.^142^ See Section 4.7 for more discussion of temperature-dependence studies.

##### Homochirality in Redox Chemistry

Because the linear momentum of the electron and its spin are locked, backscattering in homochiral assemblies is suppressed, which makes electron transfer more efficient. Measurements with the redox protein cytochrome *c* on oligopeptide films of differing enantiomeric forms show that the electron transfer rates in the heterochiral assemblies are suppressed, as compared to the electron transfer rates in homochiral assemblies.^58^

##### CISS in Molecular Recognition

Spin dependent charge polarization in proteins affects allostery, enhancing or reducing reactivity at sites far from the binding position of the substrate. A recent study on the association of an antibody with its target protein antigen can be modulated by a ferromagnet, even when the protein is bound to the ferromagnetic substrate at a site remote from the binding site.^22,143^ The charge reorganization is modulated by the magnetization because the charge displacement currents in the protein are spin polarized.

#### 3.1.4 Polymers

Spin-filtered electron transport and spin polarization manifests in chiral polymers and chiral polymer fibers.^129,144−149^ In a number of cases these films are grown by CISS-mediated processes (see Section 6.3.3). A major outcome of these studies is the demonstration that spin-filtered electron transport can proceed over hundreds of nanometers to microns in length. For example, Yan and co-workers showed that polyaniline fibers spin-filter electron currents over length scales of a few microns, along the chiral axis of the supercoiled fibers.^144^ The spin filtering is not restricted to transport along the polymer chains, even current through thick (up to 120 nm) films of chiral polymers display spin-filtered currents, see Figure 11. Similar to the case of biopolymers, the spin-filtering is temperature activated^150^ and a correlation exists between the spin polarization and the strength of the CD signal.^147^

### 3.2 Inorganic and Hybrid Organic–Inorganic Materials

Reviews of chiral inorganic materials have recently become available.^151−154^ Here we focus on CISS studies associated with different classes of chiral inorganic and hybrid inorganic–organic materials. Given the promise of CISS for interesting applications in spintronics, optoelectronics, and catalysis, the number of CISS studies with inorganic materials is expanding.

#### 3.2.1 Chiral Supramolecular Constructs

Inorganic materials and organometallic supramolecular assemblies exhibit CISS properties and can be combined with other functional elements of supramolecular constructs for bespoke spin-selective functions. For example, Therian and co-workers used mc-AFM and spin-Hall measurements to show that chiral conjugated zinc-porphyrin molecular wires polarize spin currents up to 32%.^155^ Incubating the as-assembled chiral molecular wires in binucleating ligands of the opposite handedness causes a flip in the circular dichroism response and corresponding spin-filtering properties of the assembly. In other work, Cardona-Serra and co-workers used cyclic voltammetry, electrochemical impedance spectroscopy, and transport studies to show that incorporation of paramagnetic Tb^3+^ lanthanides into helical peptides leads to higher spin polarizations compared to metallizing with diamagnetic Yb^3+^.^122^ The spin-filtering properties of the paramagnetic helical metallopeptides were later used to construct a memristor.^156^ In other studies, Sang et al. showed that helical nanofibers composed of achiral benzene-1,3,5-tricarboxamide molecules with an aminopyridine group that could coordinate to Ag(I) display spin polarizations of ∼45%,^157^ and Mtangi et al. showed that chiral Zn-porphyrin stacks display polarizations of ∼35%.^158^ Even much simpler organometallic complexes, which possess stereocenters as opposed to chiral secondary structures, exhibit CISS properties (see Wang et al.^159^ and Miwa et al.)^160,161^

##### Synergy of CISS and Spin Blockade

Spin filtering in chiral molecules containing paramagnetic ions is enhanced over that in molecules without paramagnetic ions, suggesting that CISS can be combined with more traditional spin blockade ideas to enhance spin filtering.^122^

##### Chiral Supramolecular Constructs

Yamamoto and co-workers showed that the assembly of achiral cobalt phthalocyanines into helical supramolecular assemblies on ferromagnetic substrates can be controlled by the magnetization state of the surface.^162^ This guided self-assembly is similar to the enantioseparation of amino acids from racemic solutions by crystallization onto magnetized surfaces.

#### 3.2.2 Chiral Inorganic Nanoparticles

**Figure 9:**
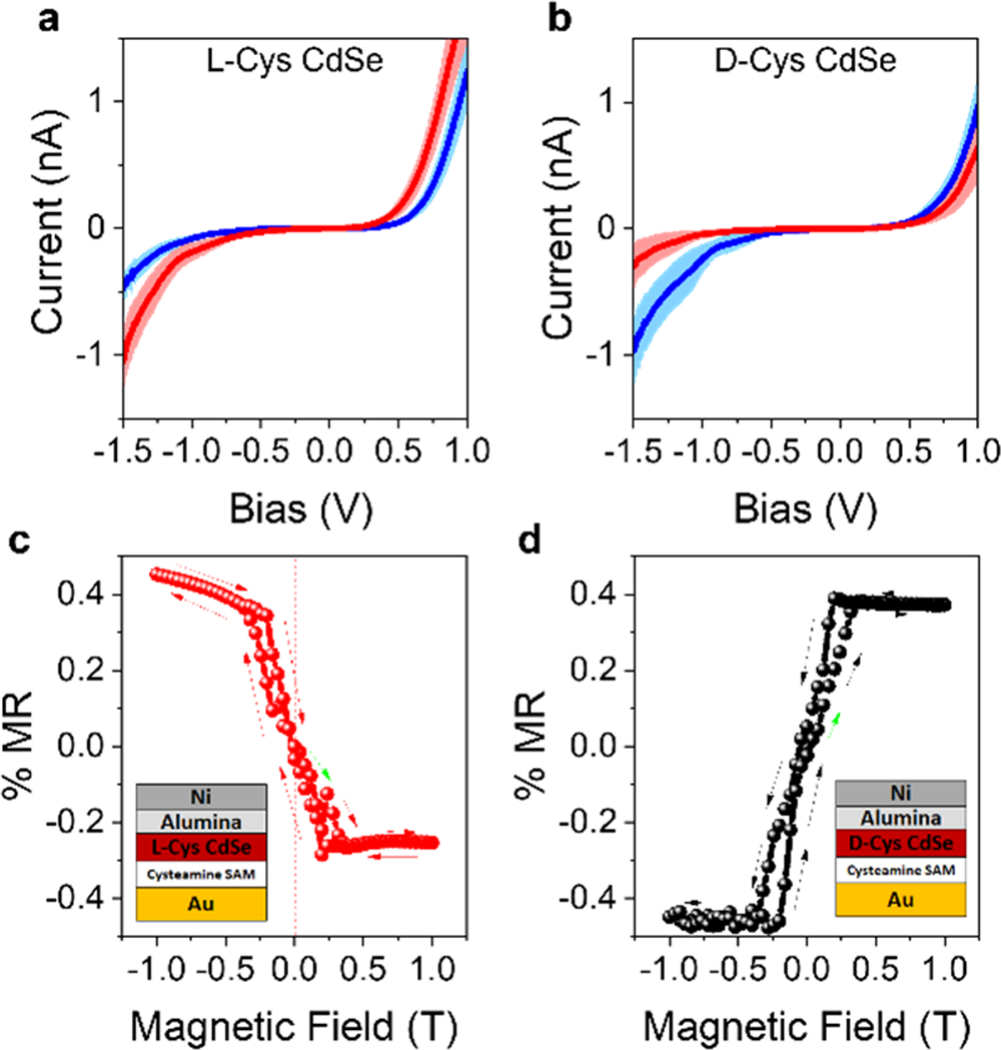
Spin transport measurements on CdSe quantum dots passivated with l-cysteine (a,c) and d-cysteine (b,d) ligands. Panels a and b show mc-AFM measurements in which the red curve corresponds to the electron spin antiparallel to its momentum and the blue curve corresponds to the electron spin parallel to its momentum. The shaded regions represent 95% confidence intervals. Panels c and d show corresponding magnetoresistance (MR) measurements on spin-valve devices. The data are replotted from ref (163) with permission. Copyright 2016 American Chemical Society.

The first report on a semiconductor’s CISS response was in 2016, in which chirality was imprinted on CdSe NPs by surface ligands.^163^Figure 9a,b shows mc-AFM measurements for studies on 2.2 nm CdSe nanoparticles passivated with cysteine molecules where an ∼33% polarization at negative bias and ∼15% polarization under positive bias was observed, in spite of the nanoparticles showing only a modest chiroptical response (∼0.5 mDeg).^163^ Moreover, a spin-valve device was constructed using the chiral nanoparticles and the data showed an asymmetric magneto-response in a manner consistent with the favorable spin alignment found for mc-AFM

In other works, assemblies comprising CdSe-polyalanine multilayers, using a layer-by-layer approach,^164,165^ display a large excitation polarization dependent change in fluorescence lifetime (∼3.5× longer for CW excitation than CCW) that was attributed to symmetry breaking-induced changes in nanoparticle coupling and spin delocalization.^164^ The enhanced delocalization between nanoparticles separated by long helical polyalanine was superior to that found in other experiments using short-chain achiral molecules,^166^ and thus, could prove useful for design strategies in parallel computing applications.

Chiral quantum dots have also found a number of applications examining fundamental CISS issues and exploring device concepts.

##### Electron Transfer Rates and CD Correlation

Electron donor-bridge-acceptor dyads, comprising an achiral CdTe NP donor and a chiral CdSe NP acceptor, were used to demonstrate how spin-filtering in chiral assemblies affects electron transfer rates.^82^ The electron transfer rate asymmetry, (*k*_*et*__,CW_ – *k*_*et*__,CCW_)/(*k*_*et*__,CW_ + *k*_*et*__,CCW_), was found to correlate with the strength of the circular dichroism spectrum for the acceptor NPs first exciton transition, and the maximum asymmetry was 88%. These studies showed that the rates can be described by a Marcus electron transfer picture in which the electronic coupling is affected by CISS.

##### Optospintronic Memory Architectures

Spin selective electron transfer between chiral NP constructs and a substrate have been used to write local magnetizations corresponding to logical memory.^118^ The spin selectivity of electron transfer with chiral CdSe NPs has been exploited to demonstrate a 9-state volatile-like spin-memory device.^167^

#### 3.2.3 Hybrid Organic Inorganic Perovskites and Metal Halides

The initial work explicitly demonstrating CISS in R-/S-methylbenzylammonium (R-/S-MBA) lead iodide perovskite 2-D layered thin films was shown by Vardeny and co-workers and displayed spin polarizations as high as 92%.^168^ Similar findings have since been reported in hybrid organic–inorganic perovskites and metal halides with other compositions as well (see Table 1). In related work, researchers have incorporated achiral additives into the chiral matrix in order to improve the film crystallinity, yet retain their spin-filtering power.^169,170^ For instance, Lee et al. showed that the addition of urea to (R-/S-MBA)_2_PbI_4_ perovskites causes structural changes to the perovskite host, which can enhance the chiroptical response and ensuing spin polarization measured by mc-AFM.^169^ The spin-filtered currents in these materials can persist over thicknesses of hundreds of nanometers, and it is hypothesized to arise from multiple tunneling processes through the chiral organic molecules occupying the space between the layered octahedral perovskite sheets.^168^

**Table 1:** mc-AFM Determined Spin Polarizations of Different Hybrid Organic–Inorganic Perovskites and Metal Halides

Composition^a^	Polarization (%)	Ref.
(R-/S-MBA)_2_PbI_4_	92	(168)
(R-/S-MBA)_2_PbI_4_/CsPbBr_3_	80	(171)
(R-/S-MBA)PbBr_3_	90	(172)
(R-/S-MBA)_2_SnI_4_	94	(173)
(R-/S-MBA)_4_Bi_2_Br_10_	84	(174)
(R-/S-MBA)_2_CuBr_4_	92	(175)
(R-/S-MBA)_2_CuCl_4_	92	(175)
(R-/S-NEA)_2_CoCl_4_	90	(170)

a R-/S-MBA is R-/S-methylbenzylammonium; R-/S-NEA is R-/S-1-(1-naphthyl)ethylamine.

The spin selectivity of perovskites in photoinduced transport has been leveraged for spin-polarized charge injection from perovskites into transition-metal dichalcogenides to manipulate valley pseudospins,^176−178^ to realize spin-mediated photogalvanic and photovoltaic devices,^179^ and to create circularly polarized light detectors^169,174,180,181^ (see Section 6).

The perovskite film studies reveal a number of important aspects for CISS-based materials as well.

##### Length Dependence and Mechanism

The studies on films show that spin-filtered electron currents can propagate over hundreds of nanometers,^168^ rather than the few nanometer limits observed for molecules. Measurements as a function of film thickness support a mechanism in which the chiral organic molecule layers spin-filter the electron currents and compensate for loss of spin purity as the propagation proceeds.

##### The Role of Chiral Phonons

Kim et al. showed that a spin-polarized current, which depended on the perovskite’s handedness and an externally applied magnetic field, manifests when a chiral perovskite is subjected to a thermal gradient.^98^ The spin polarization was attributed to a chiral-phonon-activated spin Seebeck (CPASS) effect.

#### 3.2.4 Transition Metal Dichalcogenides (TMDs)

A collection of works on chiral TMDs has recently been published and further expands the landscape of materials known to exhibit CISS properties. Duan and co-workers showed that intercalating chiral molecules into TaS_2_ and TiS_2_ TMD layers provide structurally robust materials with large spin polarizations, ca. 60%.^182^ Interestingly, spin tunnel junction devices made from the materials show magnetoresistance exceeding 300%, over an order of magnitude larger than that observed in previous CISS-based systems. Other methods for preparing chiral TiS_2_ have also been demonstrated.^183^ In related studies, Bian et al. report spin-polarized electron currents as high as 75% through 5 μm thick films of MoS_2_^184^ and greater than 90% in >100 μm TiS_2_ crystals.^185^

The spin polarizations generated in chiral molecules and materials may also prove fruitful when interfaced with TMDs to break valley state degeneracy. Research on single monolayer MoS_2_ and WSe_2_ interfaced with chiral perovskites showed changes in the degree of valley polarization and the effect was attributed to the spin-selective charge injection from the chiral perovskite, i.e., CISS.^177^ A similar enhancement in valley polarization was also observed when d-histidine was interfaced with a monolayer of MoS_2_.^186^ Here, the spin-dependent charge redistribution properties of the histidine, and strong hybridization between histidine and the MoS_2_, led to the degree of polarization at the +*K* valley being 7.73% and the −*K* valley being 1.6%. Note that spin-dependent charge redistributions in chiral molecules can lead to spontaneous magnetization,^20^ and application of external magnetic fields to TMDs can cause a Zeeman energy splitting that increases valley contrast.^187,188^

#### 3.2.5 Metal Oxides

A wide array of chiral magnetic oxides are becoming available and offer promise for a range of applications.^189^ Interest in chiral metal oxides for CISS-applications stems from initial research showing that electrodes coated with chiral molecules reduces the reaction overpotential for the oxygen evolution reaction compared to analogous electrodes coated with achiral molecules (see Section 6.3.1).^41^ By adapting the electrodeposition methods developed by Switzer and co-workers,^190,191^ chiral CuO coated electrodes were studied by Mott polarimetry and shown to exhibit spin polarizations of ∼10%.^39^ In related experiments, the spin polarization through cobalt oxide surfaces was shown, using mc-AFM and Hall device measurements.^192,193^ More recently, Ghosh et al. showed that doping cobalt oxide thin films with 5% Mn afforded an ∼2-fold enhancement in the spin polarization (55–60%) compared to the undoped chiral thin film (25%),^192^ whereas Bai et al. showed that helical stacking of NiO_*x*_ nanoflakes leads to 50–80% spin polarizations.^194^

Studies of transition-metal oxides manifest the interplay between chiral symmetry and magnetic ions, or materials. By comparing magnetic circular dichroism (MCD) spectra for helically stacked nanoflakes, Bai et al. showed that chiral α-Fe_2_O_3_, which is antiferromagnetic, exhibits a chirality-dependent/magnetic field-independent MCD response whereas the ferrimagnetic Fe_3_O_4_ and γ-Fe_2_O_3_ nanoflakes exhibit a chirality-independent/magnetic field-dependent MCD response.^95^ They conclude that magnetic field effects in the ferrimagnetic metal oxides are stronger than the magnetic field generated by CISS, whereas the CISS effect dominates for antiferromagnetic materials. Other measurements on metal oxides indicate that chiral symmetry can influence the magnetic ordering of a material. For instance, asymmetric adsorption of chiral molecules on a 10 nm superparamagnetic iron oxide nanoparticle caused the material to become ferromagnetic.^92^ In analogous experiments, vibrating sample magnetometer measurements showed that achiral CuO films were diamagnetic, whereas chiral CuO films were mostly paramagnetic with a weak ferromagnetic hysteresis.^39^ The emergent properties were hypothesized to arise from canted spins associated with a chirality-induced asymmetric lattice; however, more experiments are necessary to confirm such behavior.

##### Spin-Dependent Electrocatalysis

A combination of measurements for spin-filtered currents and for water electrolysis demonstrate the usefulness of CISS for directing chemical heterogeneous chemical reactions through spin control (see Section 6.3.1).

##### Nonmagnetic Oxide Spin Filters

**Figure 10:**
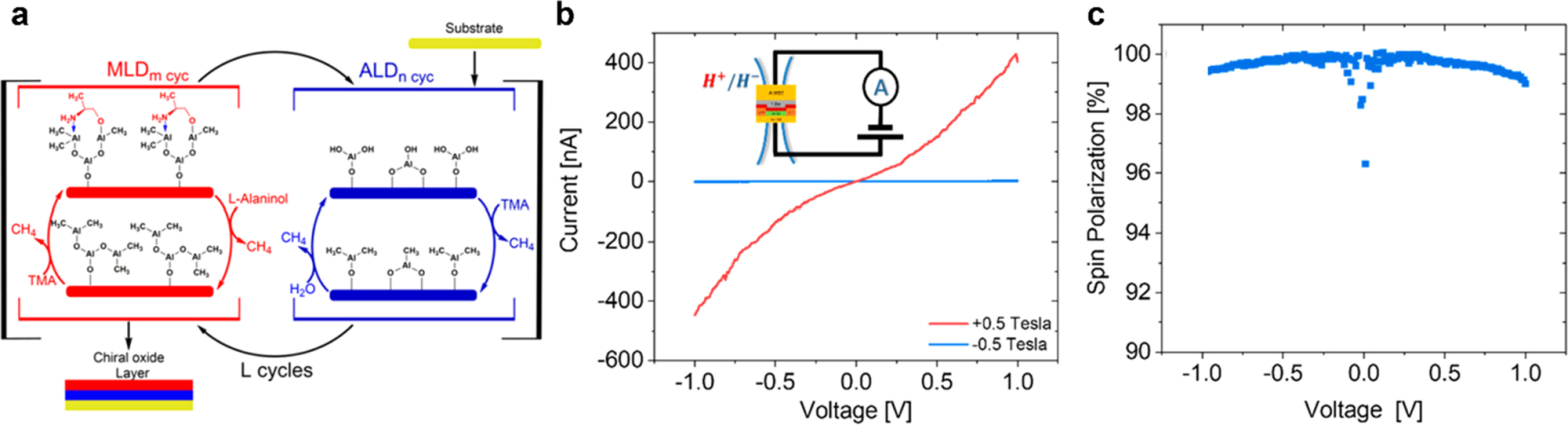
Panel a shows a schematic which illustrates the atomic molecular deposition super cycle repeated a total number of “*L*” times until a desired thickness is achieved. The deposition is composed of two subcycles; atomic layer deposition (blue) of alumina using trimethylaluminum and water repeated “*n*” times followed by dosing of the film (red) with d- or l-alaninol repeated “*m*” times. Panel b shows *j*–*V* characteristics of a device with a film fabricated using l-alaninol precursors for two different magnetic field directions; the inset illustrates the measurement circuit design. Panel c plots the resulting spin polarization as a function of bias potential. This figure is adapted from ref (195) with permission. Copyright 2022 American Chemical Society.

Recent work by Al-Bustami et al. shows the promise of chiral metal oxides as spin filters in spintronic applications. By using atomic and molecular deposition techniques (see Figure 10), they created chiral Al_2_O_3_/organic hybrid films with a near 100% spin polarization.^195^ This represents the highest spin-polarized electron current, obtained via CISS, for a device structure.

#### 3.2.6 Bulk Crystals and Organometallic Constructs

In the past five years, workers have shown that CISS phenomena are not restricted to ultrathin films and molecules but can also manifest in bulk solids.

##### 3.2.6.1 Inorganic Chiral Crystals

Recent experiments on bulk crystals, ranging from insulators to semiconductors and metals, demonstrate that CISS-based, spin-polarized charge currents persist over micrometer to centimeter distances. Inui et al. first illustrated this fact for chiral CrNb_3_S_6_ bulk crystals by detecting the spin-polarized charge current by an inverse spin Hall signal (voltage drop).^53^ The Hall signal, and hence spin polarization, was found to depend sensitively on the current direction as well as the handedness of the chiral crystal. The spin polarization in these crystals persisted over micron length scales, much longer than that of conventional achiral materials with lower spin–orbit interactions,^196^ and were hypothesized to arise from antisymmetric spin–orbit interactions, i.e., the chiral materials did not exhibit normal spin-flipping processes. In follow-up experiments by the same group, the CrNb_3_S_6_ crystals were shown to manifest bulk magnetization when an electric current was applied along the principal chiral axis of the crystal.^197^ The presence of CISS in inorganic crystals is not limited to CrNb_3_S_6_ systems; recent studies have expanded the library of crystals to include chiral Te, NbSi_2_, and TaSi_2_;^198−201^ and theoretical works on SnIP double helices are argued to give rise to spin-dependent velocity asymmetries in electron transport as well.^202^

##### Spin Transport up to Centimeters

Studies on chiral crystalline rods of NbSi_2_ and TaSi_2_ demonstrate that chirality-based spin polarization can persist for centimeters in length.^203^

##### Inverse CISS Manifests

Studies using CrNb_3_S_6_ in device structures demonstrated the existence of an inverse CISS effect, i.e., a pure spin current induces a charge current.^53^

##### 3.2.6.2 Chiral Metal–Organic Frameworks and Crystals

In a landmark paper by San Sebastian and co-workers, a paramagnetic metal–organic framework, composed of Dy(III)-tartrate, showed near-ideal spin-filtering capabilities (∼100%) and spin polarization in the charge transport persisting over 1 μm length scales.^204^ The remarkable performance was attributed to the large spin–orbit coupling of the Dy(III) lanthanides in tandem with the helicity of the metal–organic framework along multiple crystallographic directions, leading to multichannel spin-selective electron transmission. A similar behavior has also been observed in 300 nm thick Cu(II)phenyl alanine crystals, with mc-AFM measured polarizations up to ∼68%.^205^ Notably, these crystals display a transition from antiferromagnetic to ferromagnetic at 50 K that was explained by the emergence of a low-lying thermally populated ferromagnetic state, which arises from interactions among Cu(II) species mediated by the chiral lattice. Newer work on Co(II)-phenylalanine crystals reports polarizations of 35–45%.^206^

##### Circular Dichroism (CD) as a Predictor

Comparisons between Cu(II)phenylalanine and Cu(II)pentafluoro-phenylalanine crystals show that the circular dichroism response of the crystal is a good predictor for the sign of the spin polarization, rather than the structural enantiomorph.^205^ This finding corroborates such correlations reported in nanomaterials, molecules, and supramolecular assemblies.

### 3.3 Summary

The diversity of chiral molecules, molecular assemblies, and materials support the notion that CISS arises from an underlying relationship between electron spin and chiral matter that manifests because of the chiral symmetry. The knowledge amassed from the numerous experiments is defining the criteria necessary for maximizing the CISS-response in a given system and researchers are already using them to realize spin polarization magnitudes in excess of 99%.^195,204^ The knowledge gained from experiments like those described in Sections 3 and 4 is necessary for understanding CISS and developing a comprehensive theory for CISS-based phenomena.

## 4 General Trends and Structure–Property Relationships

Although a quantitatively accurate mechanism for describing the CISS effect has yet to be identified, experimental work has begun to identify structure–property relationships for chiral molecules and materials that must be accounted for by a comprehensive theory. In this section we overview the different trends observed in experiments, comment on their pervasiveness, and identify important questions that must be addressed for continued progress in the field.

### 4.1 Length Dependence

For CISS, the most well-studied trend is the relationship between the length through which an electron traverses and the resulting spin polarization of its charge current. Systematic studies of DNA and oligopeptides, using a range of different techniques (photoemission spectroscopy, mc-AFM, electrochemistry, and Hall device measurements), show that spin-filtering of the charge currents increases monotonically with the length of the molecules.^10,12,15,74,104,207^ Most studies in molecular films and assemblies have been limited to a few tens of nanometers or less, however. For example, Mishra et al. examined the correlation between the length dependence of the optical activity and the spin-filtering performance of oligopeptides and DNA for film thicknesses <15 nm.^104^ Recently, Clever et al. analyzed CISS data on DNA and oligopeptides and found that the trend of increasing polarization with increasing length was consistent among independent studies, even though the absolute magnitude of the reported polarizations varied.^43^ The increase in spin-filtering performance per nucleobase in the case of DNA and per amino acid in the case of peptides are different, however.

**Figure 11:**
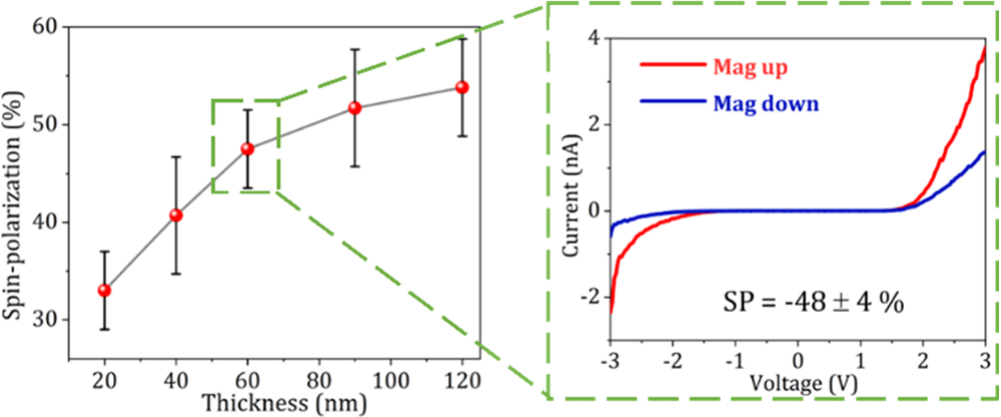
The plot shows the dependence of the spin polarization on the thickness for the polymer synthesized on a ferromagnetic electrode with application of an oriented external magnetic field. The inset shows the average current versus voltage (*j*–*V*) curves recorded for 60 ± 3 nm thickness polymers with the magnet North pole pointing up (red) and down (blue). The figure is adapted from ref (150) with permission. Copyright 2022 American Association for the Advancement of Science.

In addition to these studies on molecular systems, studies on chiral organic–inorganic perovskite films^168^ and studies through different thicknesses of chiral polymer films^150^ display an increase in spin polarization for thin films and then plateaus at large film thickness. Although it seems likely that phonons and structural imperfections in molecular assemblies would reduce the spin polarization above a certain length, recent experiments on the spin filtering of electron currents through chiral polymer films indicate that the spin polarization does not decrease strongly with increasing film thickness. Figure 11 shows electropolymerized chiral films of poly(2-vinylpyridine) in which the spin polarization increases monotonically up to a thickness of ∼120 nm, even though the chiral polymer strands in the film are disordered with respect to each other.

### 4.2 Effect of Chirality Type

Molecules manifest chirality through stereoisomerism, which includes planar chirality, axial chirality and/or helical chirality, and point chirality, which arises through dissymmetry in bond connectivity about an atomic center (typically carbon). Although CISS manifests for all of the chiral types, responses for axial or helical chirality appear to dominate over others in organic systems. For instance, mc-AFM measurements on single-stranded DNA yields no discernible spin polarization, whereas polarizations as high as 57% were found for double-stranded DNA.^10^ Similarly, Stremer et al. introduced a Hg chelating unit to single-stranded DNA, which creates a chiral secondary structure, and observed the emergence of a spin polarization.^31^ The effect of helical structure on spin polarization was also observed for peptide-nucleic acids (PNAs). PNA with modified backbones, in which the monomer units become chiral, create helices with a shorter pitch length and result in higher spin polarizations than their unmodified counterparts.^40^ These studies also imply that point and structural chirality of a material may be synergistic. Possible evidence of this synergy is shown by measurements on B-DNA, for which the helix and the stereocenters along the backbone are both right-handed. B-DNA exhibits larger spin polarizations than do measurements on Z-DNA, for which the helix is left-handed and the stereocenters along the backbone are still right-handed.^102^ Unfortunately, the helicity of B-DNA and Z-DNA is different and so a clear distinction on the underlying mechanism cannot yet be made. Also the effect of helicity on spin polarization was shown for a series of peptides that were systematically altered through amino acid substitution at fixed oligopeptide length.^43^ Here, the spin polarization increased sequentially with the increased helical content of the peptide. Because of the similar composition between peptides, this system is ideal for exploring the relationship between helicity and differences in length dependence on spin polarization.

### 4.3 CISS Manifests for Individual Molecules

Although most studies of CISS have been performed on organized assemblies or ensembles, a few experiments show that CISS manifests for single molecules. Xie et al. used mc-AFM measurements of an assembly comprising ferromagnetic electrode/DNA duplex/Au nanoparticle molecular junctions to probe the spin-filtered current transmitted by the DNA duplexes.^10^ While these studies do not unequivocally show that the spin-filtering is a single molecule process, they do show that spin-filtering can occur through a few molecules, at most. In other work, Diez-Perez and co-workers used STM-break junction experiments to study spin-filtered electron currents through peptides and found that the sign of the spin polarization changes with the chirality of the molecules.^11,208^ While these studies demonstrate that the spin-filtered currents manifest for single molecules, the interpretation of the data require that one include some spin-filtering from the ferromagnet-molecule interface.

In a recent *tour-de-force* study, Guo and co-workers used single molecule junctions and their CISS response to monitor chiral symmetry breaking in real time for a chemical reaction.^209^ Here, Ni/Al_2_O_3_/graphene/single molecule/graphene/Cr/Au molecular junctions were used to monitor the spin-filtered steady-state electron currents. They measured the spin-dependent electron current through the molecular junction while it was exposed to reaction conditions for the addition of a 1,3-dicarbonyl to the maleimide functionality. The spin-polarized electron current reported on the chirality of the molecule as it underwent reaction. These studies demonstrate the single molecule nature of CISS and a new approach for probing chiral symmetry breaking during chemical reactions.

### 4.4 Organization Effects on CISS

Control over the structural organization of chiral materials relative to the propagation direction of the electron is pivotal for maximizing the electron spin filtering. This principle is evident in many studies and was even apparent in early work that showed strong spin-filtering in organized molecular films but weak-to-no spin-filtering in disordered and/or impure films. In addition to this general observation, the sense of the spin filtering has been shown to change with the orientation of chiral helices at surfaces and with the alignment of the helical axis to the electron propagation direction. These effects are also evident for investigations into the enantiospecific interaction of chiral molecules with ferromagnetic surfaces (see Section 6.2).

#### 4.4.1 Orientation

Carmeli et al. were the first to show that the sense of the spin selectivity changes with the orientation of chiral molecules on a surface. Using poly-d-alanine, they showed that the photoelectron intensity was higher (lower) for right (left) circularly polarized excitation when it was attached to the surface through the C-terminus; however, the opposite was true when attached via the N-terminus.^8,44^ Corresponding contact potential difference measurements showed that the dipole direction of polyalanine assemblies depend on the terminus containing the cysteine linker group, implying a relationship between spin polarization and the molecular dipole direction. In a different study a similar phenomenon was observed; mc-AFM measurements on peptides assembled through the N-terminus and C-terminus gave opposite polarizations.^43^

#### 4.4.2 Alignment

The alignment of the electron spin in relation to the chiral axis of a molecule is another important variable which should be optimized to maximize the CISS response. Using STM measurements, Nguyen et al. reported weaker spin polarizations (∼60%) for chiral polyalanine clusters than for self-assembled polyalanine layers (∼75%).^113^ This phenomenon was clearly demonstrated in Kelvin probe measurements of Ala-Aib oligopeptides assembled on tapered Co/Au substrates, where the coercivity and eas*y*-axis of the magnetic cobalt layer is thickness dependent.^19^ The magnitude of contact potential difference measurements, associated with spin-dependent changes in electron delocalization into/out of the chiral molecules, correlated with the Co film’s easy-axis.

Note that the sensitivity of spin-polarized electron transport on the orientation and alignment complicates comparisons of spin-filtering for different molecule types. For example, do the differences in spin-filtered currents between oligopeptides and DNA arise from intrinsic molecular differences or from differences in their tilt-angles relative to the electron current direction? Experiments show that heterogeneity or structural disorder can decrease the CISS response, or even result in a null response, as shown in ref (23), and must be considered when interpreting experiments. Indeed, some of the largest reported spin polarizations are for comparatively rigid well-defined constructs not susceptible to the same types of disorder found in organic molecule self-assemblies, e.g., metal–organic frameworks (>99%),^204^ bulk crystals (>70%),^205^ metal halides (>90%),^170,175^ and perovskites (>90%).^168,172,173^ Further evidence corroborating this idea is shown for metal oxides in which films fabricated through electrodeposition techniques, possessing ill-defined crystallinity, exhibit worse polarizations (ca. 10–25%)^39,41,192^ than that of metal oxides formed through atomic and molecular layer codeposition techniques (>99%).^195^

### 4.5 Conduction Mechanism and CISS

Spin-filtering of electron currents through chiral molecules and chiral materials manifests despite large apparent differences in electron conduction mechanisms. For example, the electron transport in photoemission experiments proceeds largely by free particle motion (a few eV or less above the vacuum level) through chiral films, albeit with some scattering possible, whereas electron tunneling measurements on insulating films of the same chiral molecules display similar polarizations for the spin-filtered currents.^15^ Moreover, researchers report spin-filtered electron currents through insulating, metallic, and semiconductor materials which possess widely disparate transport mechanisms. This diversity suggests that different detailed mechanisms may be required to describe the spin-filtered electron currents in each case, but that they originate from attributes associated with the chiral symmetry.

### 4.6 Spin–Orbit Coupling and Interface Effects

Spin–orbit coupling (SOC) has been used to explain the emergence of spin selectivity in chiral materials^5,13^ and forms the basis of early theoretical approaches to CISS;^210−213^ however, experiments give conflicting results. For instance, Rosenberg et al. showed that the electron spin polarization decreases at higher kinetic energies,^103^ in agreement with theoretical predictions.^212^ Conversely, photoemission-based transmission experiments through ssDNA, with Hg^2+^ incorporation to form a chiral secondary structure, did not show a correlation between the amount of Hg^2+^ and the magnitude of asymmetry in spin-dependent scattering through the layer.^31^ While it is possible that a correlation with Hg^2+^ loading was below the sensitivity of the measurement technique employed, it is also possible that chelation of Hg^2+^ did not form an inherently chiral complex and therefore the global secondary structure of DNA alone determined the asymmetry in spin-dependent scattering. Changing the SOC of the substrate does not appear to be a viable strategy for probing the role of SOC in photoemission, as Mott polarimetry experiments on helicene coated Cu(332), Ag(111), and Au(110) gave slightly different spin polarizations, but differences in binding to the different substrates may have clouded any discernible trends with SOC.^28^ To further understand the role of SOC in CISS, more detailed experiments are required that systematically tailor the SOC without introducing other features known to contribute to the spin selectivity. Theoretical studies have shown how orbital-overlap and hydrogen bonding networks can alter SOC^214^ so studies should exclude structural dependent changes when assessing the role of SOC. Moreover, experiments on metallopeptides showed that incorporation of Tb^3+^ resulted in higher spin polarizations than analogous measurements incorporating the heavier lanthanide, Yb^3+^.^122^ The lack of correlation with SOC were attributed to differences in magnetic properties of the lanthanides, paramagnetic vs diamagnetic, superseding the effect of SOC. Differentiating SOC effects from that of other features that can change the CISS-response is challenging.

### 4.7 Temperature Dependence

A distinguishing feature of CISS, in contrast to other modalities for generating spin-filtered currents, is its robustness at ambient temperatures, and a broad understanding of the CISS temperature dependence, or lack thereof, may prove important for understanding its mechanism. In several early magnetoresistance measurements, the CISS-response for devices composed of both organic and inorganic chiral materials appeared to be invariant with temperature.^121,130,163^ The behavior was surprising because it contrasts with traditional giant magnetoresistance-type devices which show a general trend of increasing magnetoresistance with decreasing temperature.^215,216^ In other works, chiral dipeptide-coated carbon nanotube networks exhibited a decrease in magnetoresistance asymmetry with increasing temperature and a null response at temperatures >50 K.^217,218^ The magnetoresistance response for these studies, however, was convoluted. At low temperatures both spin-dependent, e.g., CISS, and spin-independent processes occur. At elevated temperatures the electrons begin to conduct through thermionic emission, a non-spin-selective process. In 2017, a similar series of temperature-dependent magnetoresistance measurements were made for assemblies comprising bacteriorhodopsin, and the magnetoresistance was found to increase with increasing temperature.^137^ the findings on bacteriorhodopsin measurements were corroborated in later works on azurin, oligopeptides, Pb-phthalocyanine complexes, and DNA.^52,142,161^ While temperature-induced conformational changes of materials are known to affect the CISS-response,^67^ the cause for discrepancies among the magnetoresistance measurements is currently unknown. It is important to note that some of the prevailing theories on CISS implicate vibronic contributions to the spin selectivity and therefore suggest that an increase in temperature should increase the spin polarization.^52,142,219,220^

A recent report by Qian et al. on the spin-polarized conductance through chiral molecular intercalation superlattices, chiral TMDs, may offer some explanation for why different behavior is observed.^182^ In their study the average conductance, *G*(*T*), through chiral TMDs was attributed to both spin-independent, *G*_*SI*_(*T*), and spin-dependent, *G*_*SI*_(*T*), contributions such that
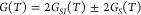
3where *G*_*SI*_(*T*) was consistent with a thermally activated hopping process, proportional to e^1/*T*^_,_ and *G*_*S*_(*T*) was modeled to be proportional to *G*_*T*_*P*_1_*P*_2_, where *G*_*T*_ is the elastic direct tunnelling process and is only weakly temperature dependent, *P*_1_ is the polarization of the ferromagnet and is proportional to (1 – α*T*^3/2^) in which α is a spin-wave parameter of the material that is temperature-independent, and *P*_2_ is the spin selectivity of the chiral layers and attributed to electron–phonon interactions. At low temperatures *P*_*2*_ was found to dominate and the *G*_*S*_ increased with increasing temperature. Conversely, at high temperatures where *P*_2_ no longer changes with temperature, *P*_*1*_ dominates and an inverse power law on *G*_*S*_ with temperature was observed. The complex nature of the system in the above example illustrates how features other than CISS, such as the polarization of the ferromagnetic “analyzer” and inherent spin-independent conductance of the material, can affect the temperature dependence observed for a given system. Moreover, these results suggest that experiments probing the temperature dependence over a narrow regime may paint an incomplete picture of temperature effects.

### 4.8 Circular Dichroism as a Predictor of CISS

Several research groups have used the circular dichroism properties of chiral materials as a figure-of-merit for the CISS response. This was initially demonstrated for donor-bridge-acceptor nanoparticle dyads in which the acceptor nanoparticle’s ligand shell was systematically varied to control the chiral imprinting on the nanoparticle’s density-of-states.^82^ The magnitude of the electron transport asymmetry, with clockwise and counterclockwise excitation, was found to scale proportionally with the circular dichroism intensity of the nanoparticle’s first excitonic transition. Similar behavior has been observed in experiments on polymers,^129^ oligopeptides,^43^ and naphthalene derivatives^207^ where changes in the helicity of the system were reflected in the circular dichroism strength and the spin polarization. Intuitively, a correlation between a material’s chiroptical properties and the material’s propensity to act as a spin filter seems sensible—the larger the dissymmetry factor, the larger the expected spin polarization.

Structural and organizational features strongly influence the sign and magnitude of the CISS response, and they need to be considered. For instance, studies on the adsorption kinetics of l-cysteine on magnetized ferromagnetic surfaces (North vs South) show a range of asymmetries in the adsorption rate with the magnetization direction (see Figure 14).^23,221^ Whether the asymmetry is positive, negative, or nil can depend strongly on the pH of the solution, despite the Cotton effects remaining mostly unchanged *in situ*. For this case, cysteine’s adsorption is known to change its tilt angle and dipole direction with pH and this must be taken into account for interpreting the data quantitatively. Such an assessment was recently used for explaining Hall measurements on some amino acids and so far appears to hold.^43^ Moreover, other studies show that using the CD for the relevant transitions, i.e., those associated with the interacting moiety, provides robust qualitative relationships between the sign of the CD and the resulting polarization.^222^

### 4.9 Relationship between CISS and Magnetic Properties

Spin exchange interactions in chiral materials give rise to new magnetic properties. For example, materials have been found in which the ferromagnetism increases with temperature for a given temperature range, and current-induced ferromagnetism has been observed in chiral crystals that contain paramagnetic atoms.^205^ Another interesting finding is the conversion of superparamagnetic nanoparticles to ferromagnetic ones at room temperature, by adsorbing them on a monolayer film of chiral molecules.^92^ These findings indicate that interesting new multiferroic properties may emerge when combining chirality with ferroic materials.

A range of works show that transient charge redistribution in chiral molecules produces a spin polarization that acts as a magnetization. Such a behavior forms the basis of the Hall response (Section 2.3.1),^20,118^ magneto-optic Kerr signals^97,112,160^ in chiral composites (Section 2.4.3), and imprinting of magnetization on ferromagnetic substrates (Section 2.3.2).^50,92,223^ The handedness of the chiral molecules and their orientation on the surface control the magnetization direction.^57,112^ The magnetic properties of the individual components that comprise larger architectures are also thought to influence the CISS-response; itinerant electron spins in chiral inorganic crystals are hypothesized to give rise to the long-range transport of spin polarization.^198,199^ In other works, ferrimagnetism was found in chiral organic donor–acceptor crystals and was attributed to the chirality-dependent spin polarization.^224^ The inherent magnetic properties of metal oxides, can also supplant or suppress the spin polarization as was shown for chiral ferrimagnetic Fe_3_O_4_ and γ-Fe_2_O_3_ nanoflakes,^95^ as well as NiFe_2_O_4_ mesostructured films.^225^

How does CISS influence the magnetic properties of a material? Because chiral materials create spin-polarized electron populations, either during transport or through displacement currents arising from electron density changes, adjacent materials and/or orbitals can accumulate spin density. Identifying what states, however, is a complicated task. Millo and co-workers showed that the adsorption of helical molecules on NbSe_2_–Au junctions resulted in new low-energy spin-polarized bound states, similar to Yu-Shiba-Rusinov states, that change in density, but not bias potential, with applied magnetic fields.^119^ Proximity effects associated with these magnetic defect-like states in relation to superconducting properties is explored in several works;^226−229^ and a similar mechanism, chirality-induced formation of new states, has been used to describe current-induced magnetization in chiral Cu(II)phenyl alanine crystals.^205^ How prevalent these states are among chiral materials and their assemblies has not yet been determined.

The role of exchange interactions on the magnetic response, and subsequent spin polarization, have also been explored.^230^ While CISS-associated proximity effects are expected to be a short-range phenomenon,^228^ exchange interactions can occur across larger length scales and are thought to form the foundation for enantioselective processes between chiral molecules and magnetized ferromagnetic surfaces (see Section 6.2).^21^ The robust nature of exchange interactions in CISS was recently shown by Ziv et al. in which the surface spin polarization of a chiral molecule coated AFM tip was used as a substitute for magnetic tips in magnetic force microscopy.^78^ In what way the resulting spin polarization is affected, however, is hard to define. In other works, studies point to exchange interactions affecting the tilt angle between chiral molecule SAMs and an applied magnetization axis,^99,114^ the organization of cellulose crystals,^231^ and the stability of proteins.^232^

## 5 Theoretical Understanding

Since its discovery, many researchers have considered CISS to be a “theoretical mystery”. While CISS has a firm basis from symmetry considerations,^233,234^ the magnitude of the effect and some of its novel manifestations challenge conventional wisdom.^51^ For example, CISS manifests in closed shell organic and biologically relevant molecules with low atomic number nuclei, whereas spin properties are commonly believed to only be important for systems with unpaired electrons and/or systems with large spin–orbit coupling (SOC). In addition, the effect is order(s) of magnitude larger in the experiment than what one calculates with simple single electron models. The temperature dependence is also surprising as Zeeman energy splittings are typically small, one expects that spin-related properties will decrease with increasing temperature, whereas CISS appears to be activated, at least in some cases. In addition, spin-dependent transport properties are observed commonly with two contact electrical configurations, which appears to violate Onsager’s reciprocity.^14^ Moreover, recent experiments show that charge polarization-induced spin polarization, which is a dynamical response, can be used to create metastable magnetic states.^99^ That is, the interaction of chiral molecules with a magnetic substrate is enantiospecific and can align the spins in the substrate, e.g., induce ferromagnetism in a paramagnet.

### 5.1 Early Models for CISS

The initial theoretical approaches can be divided into two main classes. In the first, the Hamiltonian possesses chiral symmetry and the spin–orbit coupling is treated as a parameter. These calculations show spin-dependent transport; however, the magnitude of the spin polarization is small even when the SOC is much larger than that known for hydrocarbons. The second class of approaches assume that spin selectivity arises primarily from the chiral molecule/substrate interface, or “spinterface”.

#### 5.1.1 SOC and Orbital Models

Simplified models that account for spin–orbit coupling (SOC) in describing the electron motion have recently been reviewed, see refs (14) and (235). Although scattering models produce spin filtering, the spin polarization magnitudes are too low.^236^ By re-examining the origins of SOC from the Dirac equation, workers have identified a geometric SOC, which scales with the first power of the electron mass rather than the second power, and gives rise to significantly higher magnitudes for nanoscale helices than what one might expect by considering the traditional atomic SOC.^237^ This geometric SOC has been used to model chirality-induced spin transport in chiral hybrid organic–inorganic perovskites.^238^

Scattering models can help build an intuition for CISS. Consider a thought experiment in which an electron is incident on a gold surface at some angle, a Mott scattering experiment. After striking the Au surface, the electron’s scattering angle depends on the direction of its spin angular momentum relative to the surface. The event is analogous to the classical picture of a spinning disc, Frisbee, scattering from a wall for which the scattering angle depends on whether the Frisbee is rotating clockwise or anticlockwise. Hence, if a detector for electrons is placed at a given angle relative to the surface normal, this detector will detect preferentially electrons with one spin. The ability to detect spin is a result of breaking the space inversion symmetry, by having the gold surface located either to the right or to the left of the electron source. The other necessary condition is the SOC, which couples the orbital angular momentum of the electron (relative to the gold surface normal) and its spin. Together, these two properties behave as an effective magnetic field.^239^ A chiral system breaks space inversion symmetry and if it has SOC, then it behaves like a Mott scatterer. Hence, counter to some claims,^240^ it is possible to measure the spin selectivity of a chiral system by a two point contact method.^14^

#### 5.1.2 Spinterface Models

Motivated by the need for a large SOC and a realization that the early CISS experiments were performed with supramolecular assemblies of chiral molecules on metal electrodes, researchers have considered mechanisms in which the substrate electrode enhances the SOC.^241−244^ First proposed by Gersten et al.,^245^ this approach has recently been developed by Liu et al,.^242^ Dubi,^243^ and others.^246−249^ In these models the substrate’s SOC, which can be much higher than that of a typical organic molecule, converts orbital angular momentum, arising from the electron motion through a chiral molecule, into spin angular momentum. In this picture the molecule is an orbital filter and the transmission through the interface generates the spin selection. Given that photoemission experiments for helicene monolayers on Cu(332), Ag(111), and Au(110) substrates do not display large differences in spin polarizations, despite their quite different SOCs,^28^ this mechanism is unlikely to apply to electron transmission above the barrier. For conduction measurements, however, this phenomenon may be a contributor to the overall spin filtering. A recent report by Xiong and co-workers examined chiral molecule spin valves with two different metals (Au and Al) and two different molecules. They report significant differences in the polarization of the conductance between these systems, both between the two metals with the same molecule and between the two molecules for the same metal.^250^ Together, these findings indicate that both the molecule and the spinterface are important to consider.

Although the spin is not necessarily a “good” quantum number for an electron moving through a chiral potential, especially if SOC exists, it is still valid to ask whether the chiral molecule selects for spin or orbital angular momentum. Clear evidence for spin selection is provided by EPR studies,^72^ in particular recent published work that identifies spin polarization.^91^ In addition to the photoemission studies described above, other experiments point to spin being selected. The anomalous Hall effect studies indicate that spins are injected from the adsorbed molecule into the Hall device. The same is true in the case of the Hanle effect studies (see Section 2.2.5). The interaction of chiral molecules with ferromagnetic materials and the ability to induce magnetization by adsorbing chiral molecules, all support the “spin selection” concept.

Despite their limitations, simplified models can provide useful insights into CISS and molecular properties.^251,252^ By way of example, recent work by Mujica uses an “electron on a helix” model to explore the relationship between circular dichroism and CISS. They find a clear correlation between the spin-polarized electronic response and the circular dichroism of the helix, suggesting a deep connection of CISS-based phenomena and chiro-optical phenomena through the electronic properties of chiral matter.^252^

### 5.2 Essential Features of a CISS Theory

A comprehensive theory for CISS may require that we abandon the Born–Oppenheimer (BO) approximation, which is commonly used in models and calculations. When an electron propagates through a molecule in a path that is not linear, it must exchange angular momentum with the molecular system. If the system is metallic and a band structure exists, the momentum exchange can occur with the delocalized electrons in the system. For molecules, however, the electrons are typically localized so that changing their momentum requires high energy or mixing through excited states. In contrast, a transiting electron can exchange momentum with low frequency vibrations (phonons) without the need for high order perturbations. In chiral systems, an electron transiting through a helical electrostatic potential, can exchange angular momentum with the molecule through interaction with low frequency vibrations that carry angular momentum, i.e., chiral vibrations. This process can manifest in an accumulation of Berry phase for the passing electron. This breakdown of the BO approximation has been accounted for in recent models to calculate the SOC.^253−258^ Clearly the SOC terms under the non-BO conditions are much larger, and interestingly they contain the spin-exchange interaction that has a very large value, on the order of 1 eV, for molecules. In general, it seems that any quantitatively accurate CISS model should include non-Born–Oppenheimer effects.

Recent models, which treat the CISS effect beyond the “single electron model”, provide new insights and correspond better with experimental observations.^52,259^ These models imply that the role of electron-vibrational coupling (or polarons)^52,220,260−268^ and electron–electron interactions^219^ will be essential for explaining CISS. At present it seems that the solution for the large effective SOC should arise from electron–phonon and electron–electron interactions. Recent measurements on the temperature dependence of spin filtering have motivated a number of different theory groups to explore the role of electron–phonon coupling in CISS. As the features needed to describe CISS adequately coalesce, the development of *ab initio* calculation methods is becoming possible, albeit daunting. Even qualitative models should include the chiral molecule’s magnetoelectric polarizability and its dependence on electron-vibration interactions, as well as electron–electron interactions when delocalized electrons, like in aromatic or highly degenerate systems, are involved.

### 5.3 Chiral Molecule Interactions and Ferromagnets

Numerous experimental observations show that chiral molecules interact enantiospecifically with magnetized surfaces and that chiral molecules can imprint magnetization onto ferromagnets. These experiments have spurred a new effort in theoretical developments aimed at identifying the nature of electronic spin exchange between chiral molecules and metal surfaces.^230,269−274^ Cuniberti and co-workers used spin-polarized DFT to examine the spin-dependent DOS at molecule-ferromagnet interfaces and showed that the interfacial states are locally spin-polarized but remain singlet states globally, i.e., broken spin symmetry manifests at chiral molecule-ferromagnet substrate interfaces.^230^ These findings are corroborated by other model-based studies of chiral molecule adsorption at interfaces.^270−272^

The imprinting of magnetization on a substrate by chiral molecules and charge polarization-induced spin polarization implies that intermolecular forces between chiral molecules ought to be spin dependent. Although recent experimental studies support this inference (see Sections 2.3.3, 2.4.2, and 6.4.3), more experimental work is needed to elucidate this phenomenon more fully. Theoretical interest in this aspect of CISS is growing. In early work, Kumar et al. performed DFT calculations of the interaction energy between methyl groups of interacting chiral molecules and found a chirality and spin-dependent interaction energy of about 5 kJ/mol at a 2.5 Å carbon–carbon distance.^20^ Geyer et al. have extended these ideas by constructing a model for London dispersion forces that includes intermolecular spin–orbit coupling, i.e., charge fluctuations on one molecule induce spin fluctuations in a nearby molecule.^273^ Recently, however, Hedegård has critically evaluated spin-dependent charge reorganization for molecules interacting through dipole–dipole fluctuations, and finds that the spin-polarization they manifest is quite small.^274^ This latter work implies that coupling to a bath and/or vibrational degrees of freedom are needed to explain the magnitude of the effect reported experimentally. This area represents an important frontier in CISS research and could have important implications for enantioselective chemical and biochemical processes.

### 5.4 Open Issues

Many questions, beyond quantitative agreement with experiment and the origin of the large effective SOC, can be posed.

1. Does CISS select electron spin or, more generally, an electron’s total angular momentum?
2. What is the relation between optical activity and CISS? Are there other “predictors” for CISS, e.g., a molecule’s frequency-dependent magnetoelectric polarizability?
3. Does the CISS mechanism change as the mode of conductance (insulators, metals, etc.) in a material changes? If so, how and why?
4. What is the temperature dependence of CISS in different systems?
5. What can theory teach us about the role of entanglement and coherence in CISS?

## 6 Prevalence of CISS and CISS Implications

The electron spin often plays a supporting role to the electron charge in science and technology. Even the first electronic computers used the electron charge for memory storage. While the electron spin plays an essential role in chemistry, it is often more of a “book-keeping device” (application of Pauli exclusion) because magnetic interaction strengths are commonly much smaller than Coulomb energies. The discovery of CISS, which is robust at ambient temperatures and manifests for a wide range of systems and environments, opens new possibilities for ascertaining the benefits of spin control in technologies and requires that we reexamine our assumptions about the electron spin’s importance in chemistry and biology. In this section, we discuss applications and implications of CISS for spintronics, chemical separations, chemical reactions, and molecular biology.

### 6.1 Spintronic Applications

The CISS effect offers a new approach for fabricating simple and efficient spintronic devices.^275,276^ The ultimate goal of “spintronics”, short for “spin electronics,” is to develop new technologies based on the transport of electron spin, with the hope that they will have advantages over conventional charge-based electronics, such as lower power dissipation.^277^ To date, spintronics developments have impacted computer memory technology, both in terms of hard-disk capacity and of nonvolatile magnetic random-access memory.^278,279^ Although spintronic devices are attractive for data storage, data transfer, and memory,^279,280^ they rely on transferring spin-polarized electrons from one ferromagnetic layer to another ferromagnetic layer through a nonmagnetic barrier.^281−285^ This fact is limiting because the minimum size of ferromagnetic domains is constrained by the ferromagnetic-superparamagnetic transition occurring in materials, typically on length scales of tens of nanometers or larger. Chiral materials, or chiral molecules, in which spin polarizations can approach 100%,^195,204^ offer the opportunity to make ultrasmall (molecule scale) and efficient spin filters and spin injectors.^46,276^ Below, we discuss some CISS-based analogues to existing spintronic device structures and phenomena.

#### 6.1.1 Spintronic CISS Devices

**Figure 12:**
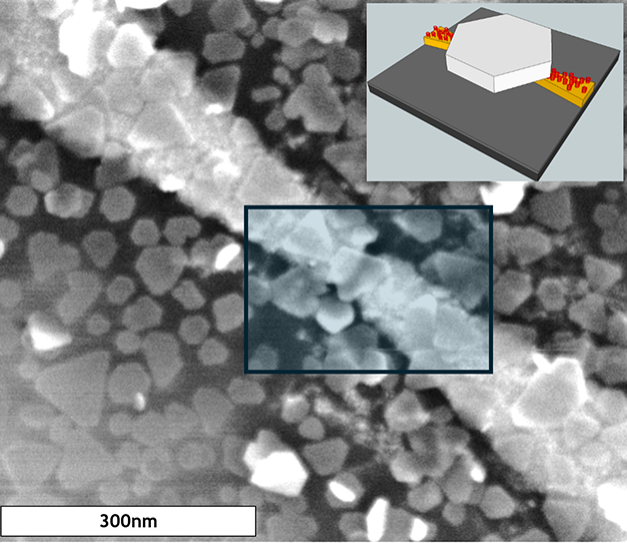
SEM image of a single magnetic nanoparticle spintronic device. Because of its CISS properties, the active memory device, which is about 30 nm in size, presents a memristor-like nonlinear logic operation at low voltages under ambient conditions. Inset: The active memory is the 30 nm magnetic quantum dot covered with chiral molecules that is located between the two gold electrodees. Unpublished work.

Two common spintronic technologies, which are used today, are magnetic tunnel junctions^286,287^ and spin transfer torque memory.^288,289^ In both, the spin polarization is performed by inorganic spin filters. In the magnetic tunnel junction/spin–valve memory, a fixed magnetized layer is separated from a second free magnetic layer by a thin isolating layer. The free layer can be magnetized either parallel or antiparallel to the magnetization of the fixed layer and the measured resistance depends on the relative orientations of the magnetization in the two ferromagnetic layers. Despite their attractiveness, these devices suffer from a number of drawbacks,^290−292^ including the minimum ferromagnetic domain size and impedance mismatch between metals and high-resistivity materials.^293^ Also, spin transfer torque memory requires high write currents, ca. 10^6^ A/cm^2^.^294^ Although new methods, such as spin–orbit torque magnetic random-access memory,^295^ are being explored, they make production more complicated. In contrast, CISS-based devices do not require a ferromagnetic layer to produce spin current, because the chiral layer produces a very high spin polarization.^46,168,276,296^ The CISS effect has been used to generate 10–30 nm ferromagnetic domains (see Figure 12).^297^ These devices operate many orders of magnitude more efficiently than standard spin torque transfer memories and are straightforward to fabricate, replacing one of the magnetic layers by a chiral film.

##### 6.1.1.1 Spin Valves

The simplest CISS-based spintronic devices are spin valves that are controlled by an external magnetic field.^298^ While they are simple to fabricate and present a two-level resistance behavior, they are hard to control locally because switching between states is achieved by the field.^50,121^ In organic spin valve devices, spin polarizations of more than 95% have been measured for conductive and paramagnetic crystalline 3-D metal–organic frameworks (MOFs), based on Dy(III) and l-tartrate chiral ligands.^204^ A major breakthrough was the finding that thin metal oxide films (oxides of aluminum,^195^ cobalt,^192,299^ and copper^41^) can be made chiral and they display CISS-based spin filtering. These materials can be integrated with conventional microelectronic technologies and become part of CISS based spintronics devices. Using 5 nm thick chiral oxides CISS-based spin-valve devices were developed that are compatible with integrated circuits technology and display high spin polarization (close to 100%).^195^

Because charge polarization in a chiral potential is accompanied by spin polarization, films of chiral molecules are intrinsically multiferroic and promise the ability to locally control their magnetization with a locally applied voltage. In fact, self-assembled peptide films have been shown to generate a local magnetization at room-temperature that depends on the sign of the applied voltage,^20,77^ creating localized magnetic fields on the order of 100 Oe at room temperature solely by applying a small gate voltage (∼0.1 V). The magnetization is believed to arise from spin-polarized charge density that is created at the substrate-chiral molecule boundary because of charge polarization of the organic molecules. Such devices offer the promise of electric field control over the magnetization locally. Although the switching rate of existing devices is low, in the megahertz range, their time response could be improved by miniaturization and device designs that make the charge displacement currents coherent.

##### 6.1.1.2 Spin Memristors

A primary challenge for reducing the dimensions of existing memristor spintronic devices is the requirement for high spin currents; however, the CISS effect provides a more efficient approach. A single nanoparticle, along with Au contacts and chiral molecules, is sufficient to function as a memory device. A single ferromagnetic nanoplatelet has been used as a fixed hard magnet, and the Au contacts act as soft magnets that result from chiral imprinting by the chiral molecules.^297^ The active memory device can be miniaturized from a micrometer scale to 30 nm in size.

A larger memristor device can be achieved using chiral metallo-bioorganic crystals, in which the memristor behavior depends on trapping of both charges and spins. In this case the crystal displays standard memristor behavior while the chiral symmetry and CISS provide additional control parameters.^300^ The spin transistor exhibits nonlinear drain-source currents, with multilevel controlled states generated by the magnetization of the source. Varying the source magnetization enables a six-level readout for the two-terminal device.

#### 6.1.2 Spin-Optoelectronics

Optoelectronics refers to devices and systems in which light affects the electronic properties of materials and *vice versa*.^301,302^ The basic idea of spin-optoelectronics is to control an electrical or photoresponse by the spin degree of freedom, in addition to the charge.^303−306^ CISS enables new design elements for use in optical memory, electroluminescence, and detection of circularly polarized light.

##### 6.1.2.1 Optical Switching

Optical gating opens the possibility to realize simple magnet-free spin valves operating under ambient conditions and controlled by photon absorption. Because of CISS, the spin-polarized photocurrent can drive spin accumulation and the emergence of photocontrolled magnetization. Such behavior has been shown to manifest in perovskites^97^ and II-VI quantum dots.^118^ For instance, a nine-state readout was achieved by using a double quantum dot architecture, i.e., two different sized quantum dots, on the active area of a Ni-based Hall sensor.^167^ In addition, light driven changes in molecular configuration have been demonstrated for changing the spin polarization. Both photoinduced and thermally induced geometric changes have been used to drive chirality inversion of a molecular motor, which leads to corresponding changes in spin polarization and acts as a molecular spin switch.^307−309^

##### 6.1.2.2 Circularly Polarized Electroluminescence

One approach to the improvement of a circularly polarized light emitting diode (CP-LED) efficiency is control over the spin degree of freedom of charge carriers, i.e., radiative recombination of spin-polarized carriers can produce circularly polarized light.^304,305^ CISS presents an exciting opportunity for circularly polarized electroluminescence because the typical components and complex architectures used for generating the spin-polarized carriers can be replaced with intrinsically chiral materials that act as spin filters. The concept of using CISS to generate the spin-polarized carriers for CP-LEDs was first demonstrated by Beard and co-workers, in which they leveraged the spin-filtering properties of 2D-perovskites to promote spin-polarized recombination in achiral perovskite nanocrystals with 2.6% circularly polarized electroluminescence (*P*_*CP-EL*_) at room temperature.^171^ Note that, researchers have used a similar device geometry, i.e., a perovskite spin transport layer, with ZnS(CdSe) quantum dots as the recombination sites to create CP-LEDs.^310^ In subsequent studies, Ye et al. showed that core–shell perovskite nanoparticles, in which the core was achiral and the shell was chiral, achieves circularly polarized electroluminescence as well, with *P*_*CP-EL*_ = 0.6% at room temperature.^311^ For this system the chiral shell acts as the spin-filter to inject spin-polarized carriers into the achiral core. Note that, CISS-mediated CP-LEDs are not limited to perovskite materials. In other work, Mustaqeem et al. used chiral metal–organic frameworks as the spin injection layer and an impressive *P*_*CP-EL*_ was observed at ZnS(CdSe) core–shell recombination sites; ca. 12.4% at room temperature.^312^ The large *P*_*CP-EL*_ was attributed to enhanced spin-coherence lifetime of the charge carriers.

##### 6.1.2.3 Circularly Polarized Light Detectors

A “simple” detection scheme for circularly polarized light (CPL), which does not depend on complex optical components, is of special interest for quantum optics^313,314^ and communications applications,^315^ among other technologies.^316−318^ While different device geometries for CPL detectors exist,^319−321^ the general working principles are the same; namely, a photoactive layer that responds differently under incident left and right circularly polarized light is used to convert an optical signal into an electrical response. The anisotropy factor in circular dichroism, *g*_CD_, is thus considered a good proxy for determining the effectiveness of a photoactive material within a device. Quite surprisingly, however, the responsivity of hybrid organic–inorganic perovskite CPL detectors was found to greatly exceed that of *g*_CD_.^322,323^ The origin of the enhancement was initially attributed to the CISS effect,^322,324^ and then later confirmed upon determining the spin-filtering properties of chiral perovskites.

The CISS-promoted CPL detection proceeds as follows: (*i*) excitation of the photoactive material with circularly polarized light, clockwise or counterclockwise, creates a spin-polarized electron–hole pair, “Up” or “Down”, because of conservation of angular momentum^325,326^ (*ii*) electron transport from the photoexcited material to the electrode exhibits differences in the resistivity for spin “Up” and spin “Down” carriers, owing to the CISS effect,^327,328^ and (*iii*) the resistivity differences give rise to electrical responsivity differences in the device. A growing number of researchers, studying various photoactive materials, are now attributing enhanced CPL detection to CISS-mediated spin-polarized transport.^169,329−333^ While the generalized mechanism holds for photodiode configurations, in which the charge carrier transport is out-of-plane, it is less clear what extent CISS contributes when charge transport is in-plane, e.g., a photoconductor configuration. Vardeny and co-workers argue that for 2D hybrid organic inorganic perovskites in-plane transport is dominated by Rashba splitting of the electronic bands,^179^ because the CISS effect is maximized when transport occurs along the primary chirality axis of the material. Conversely, Wang and co-workers contend that CISS-generated spin transport still contributes to the response.^334^ Note that the electrical transport in a phototransistor is also in-plane; however, researchers have shown that a device architecture, which uses a heterojunction (a chiral hybrid organic–inorganic metal halide interfaced with single wall carbon nanotubes), displays CISS.^181^

#### 6.1.3 Superconducting Spintronics

The idea of “superconducting spintronics”^335−337^ has emerged out of the discovery that the spin-singlet Cooper pairs of a conventional superconductor can be converted into spin-triplet pairs in the presence of ferromagnetic materials with specific forms of magnetic inhomogeneity.^338−341^ Whereas spin-singlet Cooper pair correlations oscillate rapidly in phase and decay over a very short length scale (of a few nm) in ferromagnetic materials,^342−346^ spin-triplet pair correlations can propagate over much longer distances. These spin-triplet pairs carry a net spin polarization, hence the name “superconducting spintronics”. These facts imply that superconductor/ferromagnetic/superconductor Josephson junctions constructed from conventional spin-singlet superconductors can support spin-polarized supercurrents through several tens of nanometers in a strong ferromagnetic material^347,348^ and over many hundreds of nanometers through a “half-metallic ferromagnet”, because of the absence of spin-flip scattering processes.^349,350^ The field of superconducting spintronics, in which two apparently competing notions of singlet-pairing superconductivity and spin-polarized currents are merged, is still in its infancy. To date, most devices consist of multiple sputtered or epitaxially grown ferromagnetic layers in contact with singlet, s-wave superconducting electrodes.

The CISS effect offers a new paradigm for superconducting spintronics, in which chiral molecules induce the required triplet-pair superconductivity, or assist in its formation when integrated within superconductor-ferromagnetic heterostructures. Chiral imprinting has been demonstrated in both semiconductors^351−356^ and metals.^357−360^ The adsorption of chiral molecules on conventional singlet-pairing s-wave superconductors cause a change in the order parameter of the superconductor with signatures of triplet superconductivity, with either even-frequency p-wave or odd-frequency s-wave symmetries.^119,228,229,361,362^ In superconducting spintronics CISS can be utilized to manipulate the conventional s-wave Bardeen–Cooper–Schrieffer superconductors with total spin zero of the Cooper pairs to become an unconventional s or p-wave spin-triplet triplet superconductors with nonzero total spin. Theoretical studies on ferromagnetic-superconductor and chiral molecule:superconductor hybrid systems have been reported also.^363^

#### 6.1.4 Quantum Spintronics

Chiral materials are continuing to garner attention for their importance to fundamental research in quantum matter. The high spin selectivity for nanometric chiral films should enable coherent transport, and the strong spin-exchange interactions of chiral molecules with magnetic substrates could lead to new ways of controlling spin polarization by electrical or optical gates.^364^ Experimental studies into the quantum nature and spin phase of CISS materials and CISS-based device structures can be probed by EPR methods, and maybe NMR experiments (see Section 2.4.2).^89^ EPR experiments, which exploit a qubit as a highly sensitive and coherent magnetic sensor, may provide coherent signatures of an acceptor moiety’s polarization.^88^ For example, subnanosecond photoinduced electron transfer in donor–acceptor DNA hairpin systems produced an entangled spin qubit (radical pair) at 85 K.^365^ These results demonstrate that pulsed-EPR methods can be used to manipulate coherent spin states, which is essential for quantum gate operations. In other studies, optical excitation of chiral QD systems generates coherent delocalization and charge separation of the exciton on a short time scale.^120,366,367^ While a number of important basic science questions and technological obstacles remain, the above examples illustrate that chiral materials and structures are capable of generating quantum effects in realistic solid-state devices.

#### 6.1.5 Future Directions

Hybrid chiral molecule–magnetic systems encapsulate the notion of molecular technology for the realization of spintronic and chiral spin–orbitronic device concepts,^368^ such as local control over magnetic properties, as well as chiral spin structures and dynamics.^369^ This connection could bring together two separate fields, both exhibiting chiral symmetries, and lead to novel functionality, including control of chiral spin textures by chiral molecule adsorption, particularly in 2D magnets, where interfacial effects are maximized.^370,371^

##### Chiral Magnetic Structures: Skyrmions

The combination of chiral structure with chiral magnetism,^296^ which enables ultimately stable spin structures such as skyrmions, is potentially a key enabler for applications. On a fundamental science level, the interactions between chiral molecules and chiral magnetic systems are not well understood. Beyond the basic science, control over these interactions with electrical gates may lay the foundation for future applications, such as chiral magnetic devices.

##### Antiferromagnets

The use of antiferromagnets is an emerging memory technology, but is notoriously hard to control and read.^372^ To enhance information densities, it may be possible to combine the CISS effect with antiferromagnets so that magnetic bits do not repel each other and therefore can be densely packed.^373^ It may be possible to control chiral antiferromagnets using current through chiral oxide structures that filter the desired spin states. The readout of the device may be achieved in a standard two terminal device.

##### Local Magnetic Gradients

By combining electrical gating and imprinting of local magnetization at the domain size scale, one can create local magnetization profiles with large gradients. Realizing large magnetic-field gradients is important in magnetic resonance imaging and quantum control,^374^ where the information is encoded via the magnetic-field gradient.

Integrating such skyrmion-based spintronic/orbitronic elements with chiral-induced triplet superconductors forming coherent interconnects may reduce heat dissipation in devices and thus help solve one of the major problems in data processing.

##### Photovoltaics

Efficient charge separation is a fundamental cornerstone of many photovoltaic and photochemical processes, such as artificial photosynthesis, photoelectrochemical water splitting, and solar fuel production. The CISS effect breaks the symmetry for electron and hole transport with a certain spin and can therefore be leveraged for improving charge separation. For instance, Peer et al. employed quantum dots (QDs) and helical monolayers of chiral L-polyalanine to develop a device that achieves efficient charge separation at sub-5 nm length scales without the need for doping.^120^ A related effect was measured in chiral diodes emitting circularly polarized light.^375^ It is important to note that drawing on these advances, efficient photovoltaics could be achieved if the challenge of extending chirality-driven charge separation would be extended to larger scales. The large distance achieved in photoinduced charge transfer processes mediated by chiral molecules^376^ point to the potential of this approach.

##### Magnetoelectric Multiferroics

The development of magnetoelectric multiferroic materials is of great technological importance for advanced electronics applications.^377,378^ The field of “multiferroics” embodies materials that simultaneously exhibit two or more ferroic orderings, e.g., ferromagnetism, ferroelectricity, ferroelasticity, and ferrotoroidicity.^379^ The term “magnetoelectric” refers to the coupling between ferroelectric and magnetic order parameters;^380,381^ more specifically, the tuning and switching of an electrical polarization in a material by an applied magnetic field is called a direct magnetoelectric effect, and the inverse behavior, tuning and switching of a magnetization with an applied electric field, is called the converse magnetoelectric effect.^382^ Multiple reports have shown emergent ferromagnetism^57,92^ as well as ferroelectric properties^383−385^ in chiral materials, with the latter proposed to occur through either a spin-polarized current mechanism^386^ or inverse Dzyaloshinskii–Moriya interactions (DMI).^387,388^ Note that, while the relationship between DMI and CISS has not yet been fully explored, researchers suggest that the two phenomena could coexist constructively to increase spin polarizations in chiral materials.^39,389^ Because continued progress in magnetoelectric multiferroics necessitates new approaches for creating materials with tailored electronic and magnetic properties, a CISS-based approach could prove fruitful.

### 6.2 Enantioseparations/Enantiomeric Resolution

A significant contribution to the relation between magnetism and chirality was discovered by demonstrating an enantioselective interaction of chiral molecules with a magnetized substrate.^21^ The enantio-discrimination is mediated by a spin-specific interaction, not by the magnetic field itself. The spin-dependent charge reorganization observed in chiral molecules implies that the interaction between a chiral molecule and a magnetized surface should be enantiospecific. Consider a ferromagnetic metal that is magnetized along its surface normal so that the spin sub-bands of the conduction electrons are split in energy—presenting more filled orbitals of one spin direction and more empty orbitals of the other spin direction. Because of the metal’s spin-dependent orbital population, the chemisorption or physisorption of a molecule depends on whether the molecule’s orbitals have a preferred spin direction with respect to the metal. For example, the interaction energy for a molecule forming a chemisorption bond with a metal spin–orbital will depend on whether the spins are aligned antiparallel or parallel. For achiral molecules, the charge redistribution in the molecule as it approaches and binds to the surface is not spin-dependent and no apparent spin specificity is expected. Conversely, a chiral molecule approaching the surface undergoes a spin-dependent charge redistribution, which is enantiospecific, i.e., if the spin makeup in the orbital prefers antiparallel spins, one enantiomer will interact favorably; however, spin–spin repulsion will occur with the other enantiomer.

**Figure 13:**
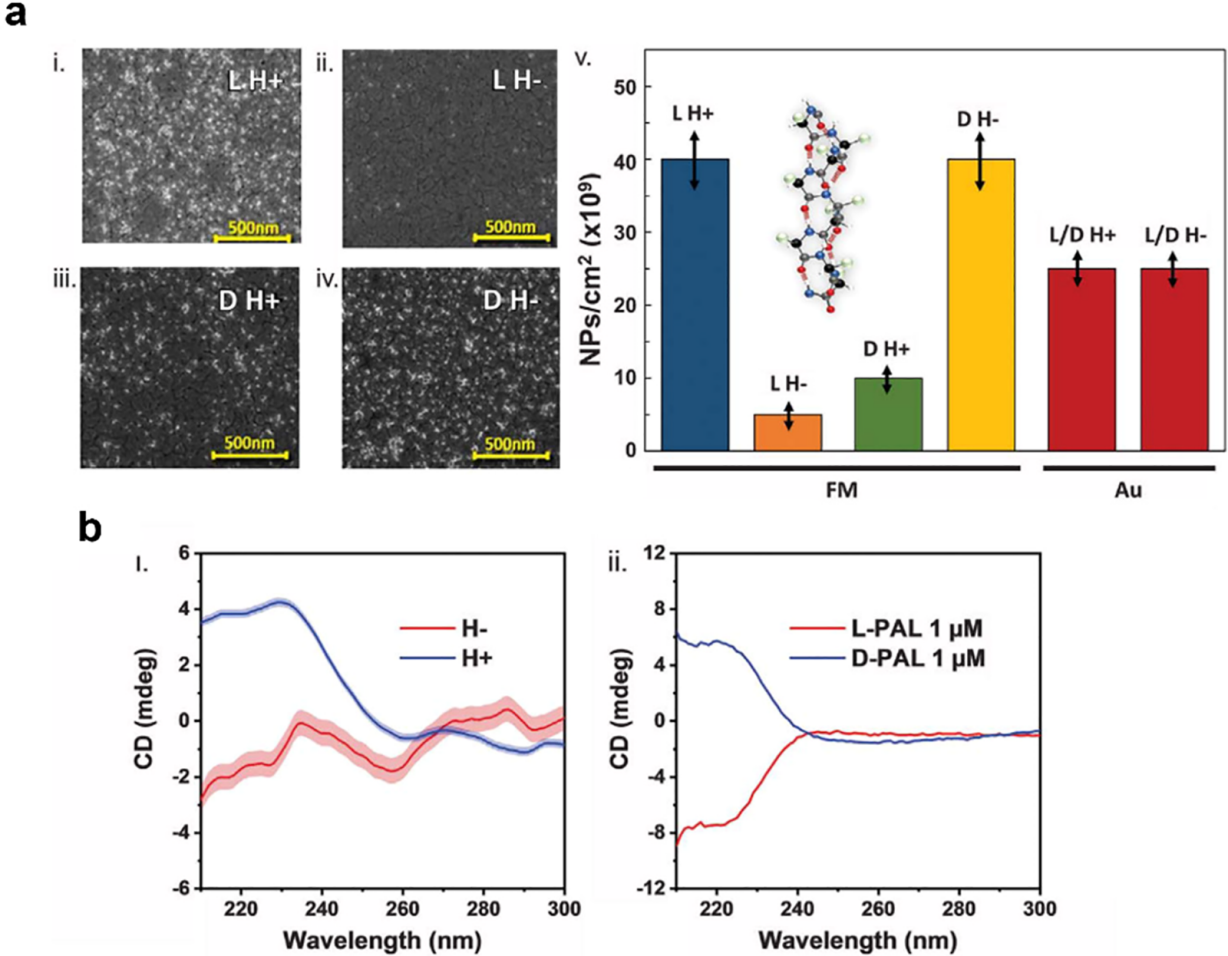
Enantiospecific adsorption of polyalanine (PAL). (a) The micrographs show the adsorption of the PAL oligopeptide [shown in inset of panel v] on ferromagnetic substrates magnetized with the magnetic dipole pointing Up (H+) or Down (H−) relative to the substrate surface. To visualize the adsorption, SiO_2_ nanoparticles were attached to the adsorbed oligopeptides. Panels i and ii show L-PAL and panels iii and iv show D-PAL adsorbed for 2 s on a substrate magnetized Up or Down. Panel v summarizes the nanoparticle adsorption densities shown in panels i–iv, compared with the adsorption density on Au with the same applied external magnetic field (red bars). Double-headed arrows represent error bars, the standard deviation among 10 measurements conducted on each of the 10 samples, hence a total of 100 measurements. b Panel i shows the CD spectra of a racemic solution of PAL, obtained following exposure to a ferromagnetic substrate with magnetization pointing Down (red) or Up (blue). Following the adsorption onto the ferromagnetic surface, it is evident that the solution becomes enantioenriched. The line width reflects the uncertainty of the results. Panel ii shows the CD spectra of the pure enantiomers for comparison. The figure is adapted from ref (21) with permission. Copyright 2018 American Association for the Advancement of Science.

It is commonly assumed that recognition and discrimination of chirality, both in nature and in artificial systems, depends solely on charge and spatial effects, i.e., shape. However, the CISS effect correlates charge redistribution in chiral molecules with an enantiospecific electron spin orientation, so that magnetic surfaces should be enantiospecific when spin polarized. Ghosh et al. first showed the enantioselective interaction of chiral molecules with a ferromagnet that was magnetized perpendicular to its surface.^21^ Here, one enantiomer adsorbed preferentially when the magnetic dipole was pointing Up, whereas the other enantiomer adsorbed faster for the opposite magnetization orientation (see Figure 13). The interaction was not controlled by the magnetic field per se, but rather by the electron spin orientations. These studies illustrate the prospects for a new approach to enantiomeric separations.

#### 6.2.1 Enantiospecific Adsorption

**Figure 14:**
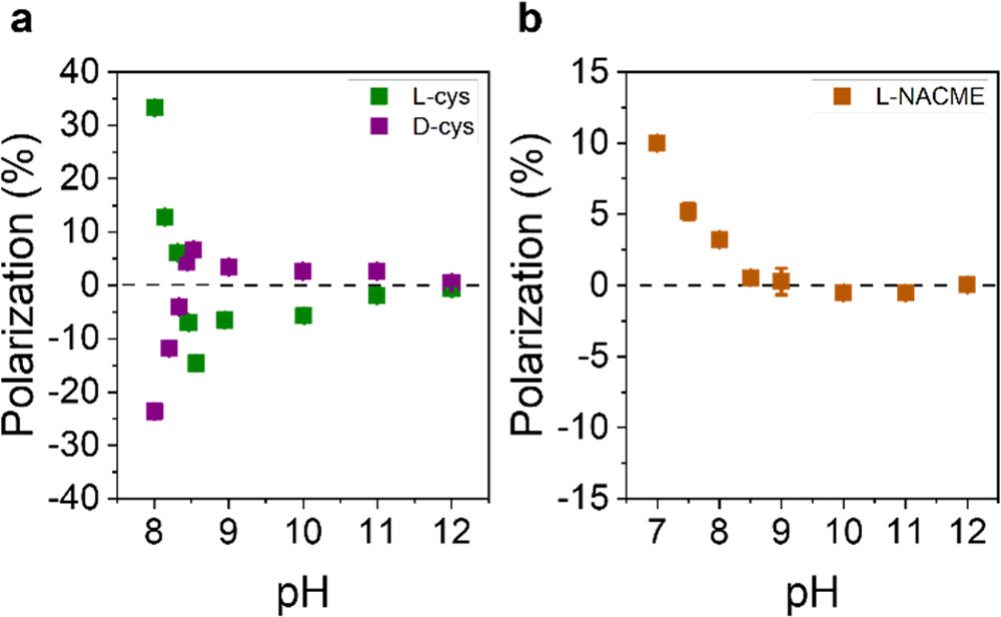
Studies into the effect of solution pH on the asymmetry in effective adsorption rate constant of cysteine onto a magnetized ferromagnetic substrate with a North and South applied magnetic field. Panel A shows the results of l-cysteine (green) and d-cysteine (purple) adsorbates; panel B shows the results for n-acetyl l-cysteine methyl ester. The figure is adapted from ref (23) with permission. Copyright 2021 American Chemical Society.

Quartz crystal microbalance measurements by Lu et al. show that the asymmetry in adsorption kinetics on North and South magnetized ferromagnetic electrodes is sensitive to the binding orientation.^23^Figure 14a shows data in which the pH was systematically changed and the polarization in adsorption kinetics, *P* = (*k*_*ads*,N_^′^ – *k*_*ads*,S_^′^)/(*k*_*ads*,N_^′^ + *k*_*ads*,S_^′^), for l-cysteine (green) and d-cysteine (purple) was measured. Consider the case for l-cysteine: at pH 8, a large positive polarization (33%) was observed; however, increasing the pH to 8.56 caused a dramatic decrease in the polarization (−15%) before asymptotically approaching a zero polarization at even higher pH. The transition from high to low polarization occurs approximately at the p*K*_a_ of the sulfur moiety on cysteine^390^ and previous experiments have shown that this coincides with a change in molecular binding geometry.^391^ Large binding mode heterogeneity at high pH could explain why the polarization decreases to zero. Control experiments on n-acetyl l-cysteine methyl ester, in which the carboxylate and amine are protected and presumably do not interact with the substrate, do not show this same inversion in polarization with pH (see Figure 14b). In addition, recent DFT calculations indicate that adsorbate–solvent interactions may be important for defining the enantiospecificity in adsorption.^221^ The idea of geometric properties controlling spin selectivity could also be responsible for the differences observed in magneto-driven enantioselective crystallization of racemates (see section 6.2.2). Additional studies that determine the adsorbate geometry and preferential facet for crystal growth may contribute to a better understanding of enantioselective crystallization.

#### 6.2.2 Crystallization

The spin-exchange interactions that define the preference of magnetized ferromagnetic surfaces for one enantiomer over the other can also be leveraged in crystallization processes for chiral resolution. It is possible to use magnetic surfaces to provide a chiral bias for enantiomer specific amino acid crystallization. Interestingly, studies show that l-glutamic acid, l-threonine, and d-asparagine preferentially crystallize on North magnetized ferromagnetic substrates despite the asparagine being the opposite enantiomeric form of glutamic acid and threonine.^392^ Racemic mixtures of asparagine and glutamic acid hydrochloride could thus be sorted into enantioenriched conglomerates through crystallization in a bath comprising North and South magnetized ferromagnetic substrates.^392^ Conversely, racemic mixtures of threonine could not be resolved under the initial solution conditions because twinning of the enantiomorphs occurs upon crystallization. These results highlight the limitations of the CISS-mediated crystallization approach to materials that form enantiopure crystallites. Despite the nonideal crystallization properties of threonine, improvements to the apparatus design and solution conditions led to an enantiomeric excess of ∼60% in subsequent studies.^393^ Note that the improved design has the added benefit of being applicable for bulk crystal separation through the continuous separation of chiral conglomerate crystals.^393^ In addition to asparagine, glutamic acid, and threonine, chiral resolutions of imeglimin and ribo-aminooxazoline have also been performed.^393,394^

#### 6.2.3 Future Directions

Because of the CISS effect, magnetic materials offer viable new strategies for enantioseparation; however, the commercialization of CISS-based separation systems will require large improvements in the enantioresolution. To this end a 2-fold approach must be taken. First, efforts must be made to understand and predict the spin-exchange interactions that dictate the enantiospecificity. As shown in EQCM measurements,^23,221^ the exchange interactions will inevitably rely on molecule-dependent structural features that can change with solution and pH. Second, we must define design parameters to optimize and control, much like what has been done in traditional separation platforms, for efficient separations. Initial efforts have been undertaken in this regard for crystallization systems^393^ and the groundwork for CISS-column chromatography is currently underway.^395^

Note that CISS separations need not be a standalone technology. For instance, the flow cell geometry used during the initial discovery of the effect,^21^ can be coupled with existing flow cell apparatuses that rely on differences between homo and heterochiral materials for enantioseparation. Indeed, studies show that CISS operates in these conditions and can be as strong, if not stronger than, the stereoisomeric interactions.^116,222^

### 6.3 Chemical Reactions

Electron spin plays a critical role in chemical bonding, and the manipulation of spin in reactive processes by the CISS effect offers a new strategy for controlling reaction pathways. This promise has been demonstrated for water electrolysis with chiral electrocatalysts (see Section 6.3.1), and it offers a general strategy for improving selectivity in reaction mechanisms that involve intermediates of different spin multiplicity. More than this, CISS implies that the electron spin is coupled to the molecular frame of a chiral molecule (or material) and affords an ability to translate control over the electron spin into control over enantioselectivity. We discuss the initial steps along this pathway in Section 6.3.2. Realization of CISS in chemical reactions is driving a paradigm shift in how we view chemical synthesis.

#### 6.3.1 CISS Enhances Efficiency of O_2_ Reactions

CISS can improve the efficiency of the oxygen evolution reaction (OER) and oxygen reduction reaction (ORR), which remain important bottlenecks for numerous electrocatalytic and electrochemical processes, including water electrolysis,^396^ exchange membranes for batteries and fuel cells,^397−401^ and the electrochemical reduction of CO_2_,^402,403^ among others.^404^

##### 6.3.1.1 Oxygen Evolution Reaction (OER)

The first work to improve the efficacy of the OER with CISS used photoanodes comprising quantum dots assembled on chiral molecules to lower the overpotential for the OER and to generate larger quantities of hydrogen at the cathode than analogous photoanodes coated with shorter chain achiral molecules.^405^ The effect was attributed to the spin polarization on the photoanode favoring the ground state formation of triplet oxygen O_2_ (^3^∑_*g*_^–^) and these findings were later corroborated with photoanodes comprising conductive polymers.^145^ In subsequent studies with helically aggregated dye molecules^158^ additional features of spin polarization on the characteristics of water splitting became apparent; not only was spin polarization responsible for decreasing the reaction overpotential for the OER, it was shown to inhibit the formation of hydrogen peroxide, as well as other super oxides.^406^ These effects have since been shown for other chiral catalysts,^407−413^ including chiral metal oxides^117,193,133,414−416^ and metal sulfides.^183,184^ As an aside, we note that CISS may account, in part, for the high activity of photosystem II.^417^

**Figure 15:**
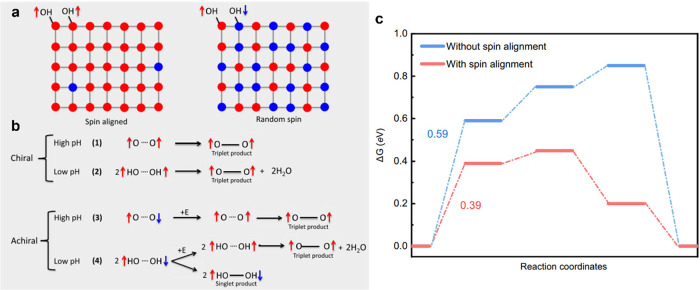
Proposed mechanistic scheme to explain the role of CISS during water splitting. Panel a shows a model lattice where the color of the ball indicates the spin of a radical intermediate adsorbate on the catalyst (shown here as a hydroxyl); blue indicates a spin down site whereas red indicates a spin up site. For chiral catalysts (left) the electrons at adjacent sites are spin aligned, because of the spin polarization, and thus formation of triplet oxygen is favored. For achiral catalysts (right) spin disorder exists and often necessitates either a change in spin state or a singlet-mediated pathway for the reaction to proceed. The figure is adapted from ref (299). Copyright 2020 American Chemical Society. Panel b shows the influence of the solution pH conditions on this process. For achiral catalysts a larger potential (+E) is needed to overcome the spin disorder limitations, compared to chiral catalysts, and additional singlet reaction pathways become more prominent. Panel c shows a theoretical treatment to determine the free energy of oxygen evolution at each reaction step on a CoFe_2_O_4_(111) surface toward triplet oxygen with (red) and without (blue) spin alignment on the catalyst surface. This figure is adapted from ref (421) with permission (http://creativecommons.org/licenses/by/4.0/).

Although chiral ligands improve OER efficiency, they can reduce the density of active sites on the catalyst surface. To circumvent these issues chiral CuO thin films, which do not contain chiral molecules, were fabricated and show a similar improvement in the OER compared to achiral controls.^41^ Here, the chirooptically active CuO gives rise to spin polarization, as measured by Mott polarimetry;^39,41^ however, all other measured properties of the achiral and chiral catalysts were the same, e.g., XPS spectra, absorbance, etc. Later works with chiral CuO/Ni-foam,^418^ cobalt oxide,^299^ molybdenum sulfide,^184^ and iron–nickel composites^419^ have shown similar improvements in the electrolysis over the achiral analogues. Figure 15 shows a proposed mechanism to explain how spin polarization affects the overpotential and Faradaic efficiency of the reaction as a function of pH.^299^ Experiments show that the Faradaic efficiency for OER with achiral (reaction 3 in Figure 15b) and chiral catalysts (reaction 1 in Figure 5b) are similar at high pH values, which is consistent with unfavorable formation of hydrogen peroxide, p*K*_a__1_ of 11.7 for the hydroxyl radical.^420^ A large difference in reaction overpotential was still observed, however, and was associated with a spin flip being required to form the triplet oxygen. As the pH decreases the difference in overpotential persists, but now the reaction intermediates comprise both hydroxyl radicals and oxy radicals. For achiral catalysts (reaction 4 in Figure 15b), in which spin constraints are not present, this leads to a lower Faradaic efficiency because of the competition with hydrogen peroxide formation. Conversely for chiral catalysts (reaction 2 in Figure 15b), the formation of the singlet-mediated byproduct is spin forbidden and therefore the Faradaic efficiency remains high. These studies indicate that the effect of spin polarization on the OER becomes increasingly important as the pH decreases to neutral and acidic conditions. Figure 15c shows an alternative mechanism in which the free energy associated with each step of the catalytic cycle, toward the production of triplet oxygen, is proposed to change if the catalyst is spin polarized (red) or not (blue).^421^

A common approach to predicting the performance of a heterogeneous catalyst uses the Sabatier principle in the form of a “volcano” plot. For the OER, workers often plot the negative of the overpotential versus the difference in the Gibbs energy of the adsorbed oxy and hydroxy radical intermediates on different electrocatalysts, so that the apex gives the optimum condition for the reaction.^422,423^ Recent work on NiO_*x*_ and Fe_(3–*x*)_Co_*x*_O_4_, two catalysts near the apex of the volcano for the OER, suggest that chirality manifests as an independent design variable that can be used to reduce overpotential.^133,416^ The difference in overpotential between chiral and achiral catalysts has been explained in theoretical works as spin polarization modulating the transition state energies of the reaction intermediates compared to catalysts that are not spin polarized.^424−426^ The change in energies associated with spin polarization can be so extreme that even the OER rate-determining step can change. Ren et al. showed such behavior for magnetized achiral ferromagnetic catalysts,^421^ and Vadakkayil et al. showed that CISS-mediated spin polarization from chiral Fe_(3–*x*)_Co_*x*_O_4_ catalysts can accomplish the same.^416^ Concomitant with the change in rate-determining step, the chiral Fe_(3–*x*)_Co_*x*_O_4_ catalysts displayed extraordinary mass activity, on par with some of the largest reported in the literature and >400-fold higher than benchmark IrO_2_ catalysts under the same electrolytic conditions.^427^ Improvement in OER characteristics for chiral metal oxide catalysts over analogous achiral catalysts is now considered indirect evidence for spin polarization arising from the CISS effect.^117,299^

##### 6.3.1.2 Oxygen Reduction Reaction (ORR)

**Figure 16:**
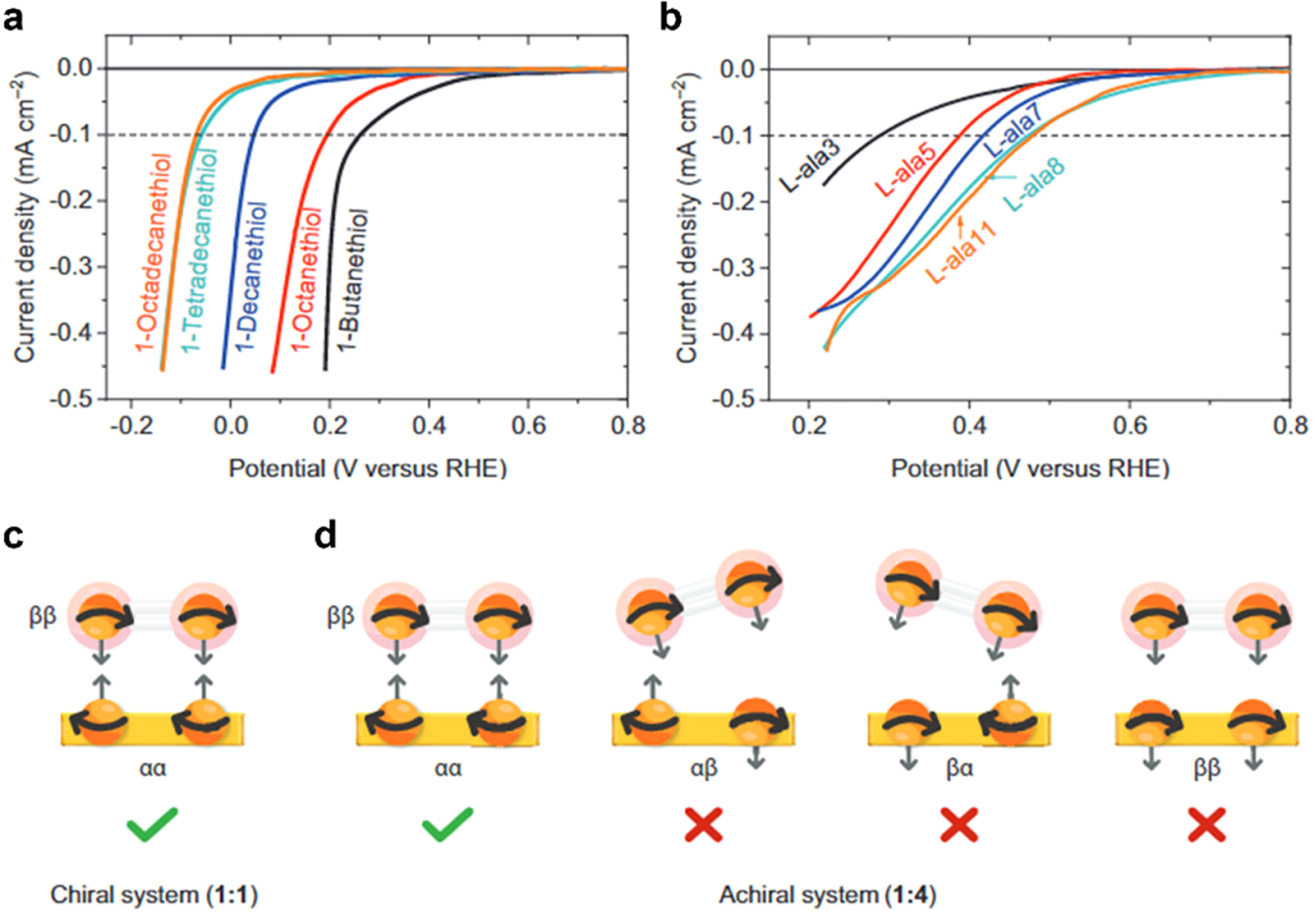
Linear sweep voltammograms for oxygen reduction in O_2_-saturated 0.1 M KOH solutions using electrodes coated with achiral (a) and chiral (b) SAMs. The possible spin-mediated O_2_–substrate interactions available in the case of a chiral catalyst (c) and an achiral catalyst (d). The figure is adapted from ref (428) with permission.

Chiral-induced spin selectivity has also been shown to improve the efficiency for the oxygen reduction reaction. Sang et al. showed that gold electrodes coated with alkanethiol SAMs exhibit progressively higher overpotentials with increasing alkane chain length, whereas the opposite trend was observed with chiral SAMs comprising oligopeptides: the overpotential decreased for increasing peptide length (see Figure 16).^428^ The effect was attributed to spin alignment of the chiral electrode surface lowering the transition state energy for reduction of the triplet oxygen for chiral catalysts (Figure 16c) compared to achiral catalysts in which the spin alignment at adjacent sites on the catalyst is less favorable (Figure 16d). To test the viability of CISS for more relevant catalyst systems, the authors extended the study to include chiral platinum nanoparticles and compared the results to commercially available platinum on carbon black, a common benchmark material. The chiral catalysts showed marked improvement in both mass activity and specific activity over the platinum carbide catalyst.^428^ The effect of spin polarization on ORR efficiency has since been shown in other works.^429,430^

#### 6.3.2 Organic Electrosynthesis

CISS is promising in facilitating organic reactions and promoting enantioselectivity through spin control. As discussed in Section 6.2, the charge polarization of a chiral molecule is accompanied by a spin polarization^20^ and this process can lead to enantiospecific interactions between chiral molecules and ferromagnetic substrates.^21^ The same enantiospecific interactions have been leveraged in electrochemical reactions to facilitate the reduction (or oxidation) of one enantiomer while inhibiting that of the other. Enantiomeric enrichment of a racemic solution through CISS-mediated electrochemistry was first demonstrated for the reduction of camphorsulfonic acid to 10-mercaptoborneol.^24^ Magnetizing a ferromagnetic electrode with a North (South) magnetic field, applied normal to the electrode surface-plane, causes the irreversible reduction of S-camphor sulfonic acid to proceed more (less) readily than R-camphorsulfonic acid. This asymmetry leads to a time-dependent increase in the enantiomeric excess of camphorsulfonic acid, in which the enantiomer that appears in excess is determined by the applied magnetic field. Subsequent measurements on the same redox reaction show that the enantioselectivity of the ferromagnetic electrode decays, by ∼33%, upon prolonged reaction conditions.^431^ X-ray photoelectron spectroscopy analysis attributed the behavior to electrode fouling and degradation, arising from sulfur formation at the electrode surface, and highlights the importance of establishing methods to stabilize the electrode, and hence its polarization, for enantioselective electrochemistry. Chiral metal surfaces, such as nickel, are also being used to resolve racemic mixtures.^432^

Other work shows that spin-mediated catalysis, and CISS-based phenomena, can be used to electrochemically transform achiral materials into enantioenriched chiral products. For instance, magnetized ferromagnetic electrodes coated with Fe_2_O_3_ catalysts convert methylphenylsulfide, under oxidative conditions, to chiral methylphenylsulfoxide.^433^ Here, application of a North magnetic field gave rise to an 8.5% enantiomeric excess, whereas a South magnetic field produced a 16% enantiomeric excess of the other enantiomer. The authors argue that spin alignment of the reactant at the magnetized electrode surface places symmetry constraints on the reaction, such that the transition state energy for the formation of the two different enantiomers of the product is no longer degenerate. Using the same electrode configuration, the authors further demonstrate CISS-based enantioenrichment for a Diels–Alder cycloaddition reaction of 2,3-dimetylbutadiene with acetaldehyde to form 2,3,5-trimethyl-2,6-dihydro-2H-pryan.^433^ Although the enantiomeric excess in these studies are small, they demonstrate the remarkable capabilities of CISS in chemical synthesis.

#### 6.3.3 Polymerization

**Figure 17:**
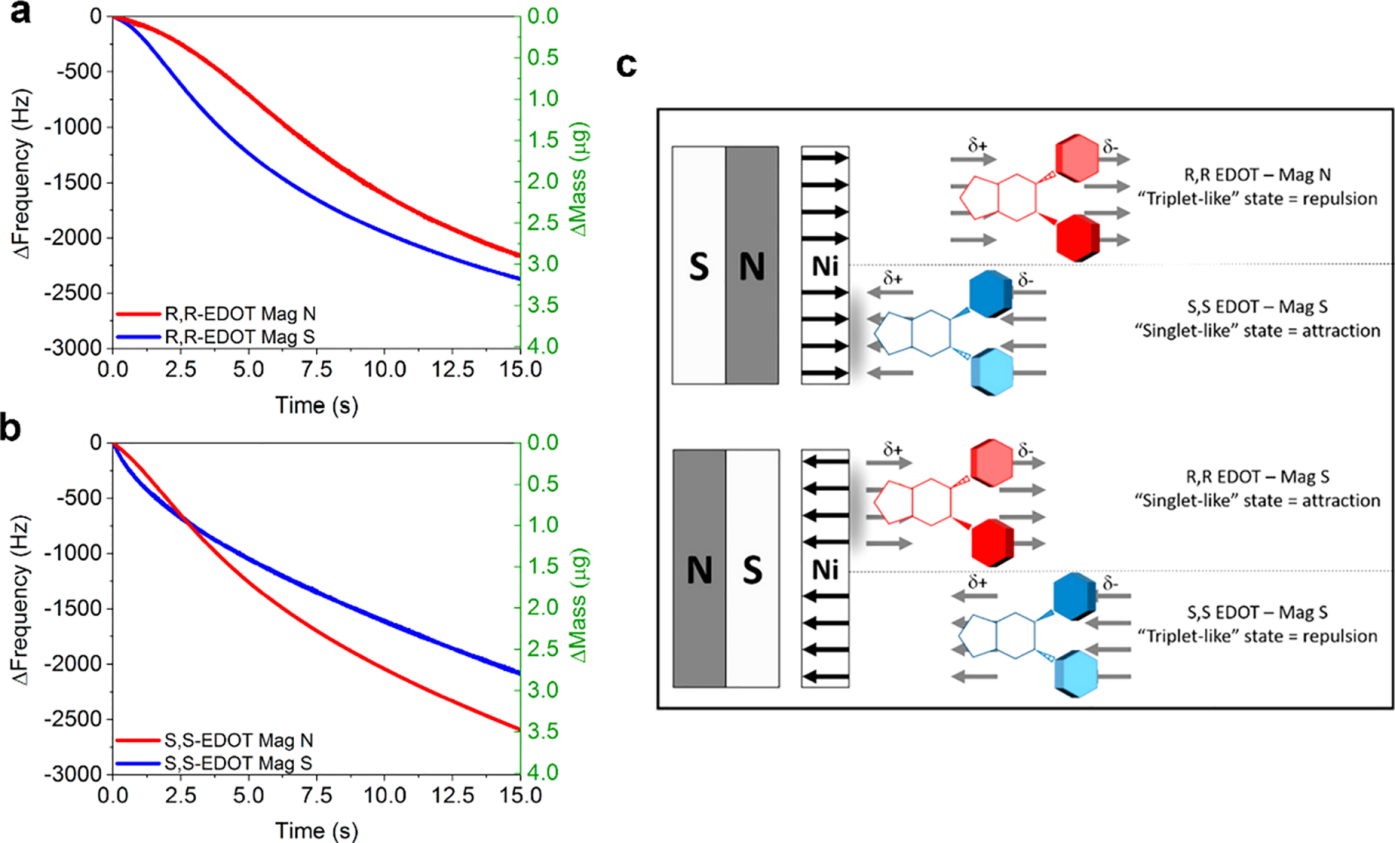
Electrochemical quartz crystal microbalance measurements of the electropolymerization of R,R-EDOT (a) and S,S-EDOT (b) onto a ferromagnetic electrode with a North (red) or South (blue) applied magnetic field. Panel c shows an experimental scheme illustrating differences in the spin-exchange interactions between the chiral monomers and the magnetization state of the electrode giving rise to differences in the nucleation step of the reaction. The figure is adapted from ref (434) with permission. Copyright 2020 American Chemical Society.

The CISS effect has also been shown to operate during electropolymerization reactions on magnetized ferromagnetic electrodes. For instance, profilometry measurements following the electropolymerization of 2,3-diphenyl-3,4-ethylenedioxythiophene (EDOT) monomers for a fixed amount of charge showed that the thickness of R,R-EDOT films under an applied South magnetic field (116 ± 5 nm) was greater than that of a North magnetic field (80 ± 5 nm). Conversely, the opposite was true for the S,S-EDOT monomers; North applied magnetic field (120 ± 5 nm) was greater than South magnetic fields (90 ± 5 nm).^434^ Note, under both magnetizations the films were thicker than the case when a magnetic field was not applied, indicating increased mass transport associated with magnetohydrodynamic effects;^435^ however, the change in thickness with field orientation is a manifestation of CISS. The results were further corroborated by EQCM measurements, which showed that the electropolymerization of the R,R-EDOT monomer (Figure 17a) was faster when the ferromagnetic electrode was magnetized South (blue) and slower when a North (red) magnetic field was applied, and for the S,S-EDOT monomer (Figure 17b) was faster when a North (red) magnetic field was applied compared to a South (blue) magnetic field. Figure 17c shows an experimental scheme rationalizing the differences in thickness to changes in the spin-exchange interactions between the chiral monomers and magnetized ferromagnetic electrodes during the nucleation step of the polymerization.

In addition to affecting the polymerization of chiral monomeric units, the CISS effect can aid in controlling the handedness of polymers composed of achiral monomers, so long as the polymer can adopt a helical geometric structure. Initial studies demonstrated this phenomenon for the electropolymerization of achiral 1-pyrenecarboxylic acid monomers onto ferromagnetic substrates, in which the application of a North and South magnetic field gave rise to opposite circular dichroism spectra for the resulting polymer.^24^ In subsequent studies a similar phenomenon was observed using carbazole, 3,4-ethylenedioxythiophene, and 2-vinylpyridine monomers.^150,431^ Electropolymerization with 2-vinylpyridine monomers is particularly interesting, as the spin polarization emanating from the ferromagnetic electrode created an enantiopreference for the generation of carbon stereocenters in the polymer chain.^150^ Although a mechanism for how chirality emerges in systems with achiral monomeric units has not yet been realized, one possible explanation relies on the CISS effect and spin-dependent charge polarization.^431^ Because the monomers are achiral, at early reaction time, electropolymerization likely results in a mixture of right-handed and left-handed short chain oligomers. However, as the reaction proceeds, delocalization of the spin-polarized electrons from the substrate into the oligomers would depend on the enantiomeric form, and hence a spin selectivity preference of the oligomer should emerge. Such behavior would cause differences in the reactivity of the two enantiomers and thus favor the formation of one enantiomer over the other in a manner that depends on the orientation of the applied magnetic field.

#### 6.3.4 Future Directions

Despite substantial progress, much work remains to control and exploit CISS in chemical reactions. In related work on the effect of spin constraints on reaction pathways, spin alignment through the application of an external magnetic field has been shown to guide the carbon dioxide reduction reaction toward more value-added products,^436−438^ as well as improve nitrogen fixation,^439,440^ hydrogenation of ethylene,^441^ and Fenton chemistry,^442^ among others.^404,443,444^ Such systems thus represent a viable testbed of reactions for which CISS studies can be performed. It is important to stress that there are added benefits to replicating the magnetic field effect studies using chiral catalysts beyond just reiterating that spin-constraints affect reaction activity: (*i*) the spin polarizations can be much higher in chiral materials, >99%, than that found in magnetized ferromagnets, (*ii*) CISS affords additional flexibility on catalyst design, allowing one to impart spin effects to state-of-the-art catalysts without being limited by the catalyst’s magnetic properties; and (*iii*) additional details regarding the mechanism can be learned by comparing magneto- vs CISS-catalysis. In addition to spin mediated effects, application of an external magnetic field during electrolysis can also affect mass transport^435,445^ and have been credited by some for the improved activity during the carbon dioxide reduction reaction.^446,447^ A comparison of the activity between chiral and achiral catalysts, in which mass transport effects do not occur, could help better elucidate the role of spin in the reaction mechanism. Ultimately the efficacy of CISS in bulk electrolysis will depend on many factors such as the stability of the catalyst during electrolysis, the scalability of the approach, as well as the ease at which the materials can be incorporated into existing constructs.

With the exception of Diels–Alder cycloaddition studies,^433^ the effect of CISS on organic transformations has been limited to one electron reduction, or oxidation, reaction steps and shows low enantiomeric excess. While chiral resolution and enantioselective synthesis remains an attractive application of CISS-based ideas, experiments necessitate improvements in the enantiomeric excess to garner interest for applications. To achieve high product enantiopurity, studies will likely rely on a combination of traditional methods for asymmetric catalysis, such as a chiral medium or chiral electrode,^448,449^ in tandem with the CISS effect. The CISS effect may also find use for other classes of reactions. For instance, radical and radical-pair mechanisms are argued to be influenced by CISS—either directly, through coherence or polarization effects involving chiral molecules,^88,450^ or through indirect processes as a result of fields generated from a chiral catalyst surface. It has long been shown that magnetic fields can affect reactivity and reversibility of reactions involving correlated spins^451^ and therefore CISS ought to imbue analogous behavior. While experimental evidence for CISS-mediated organic catalysis involving a radical-pair mechanism is minimal, previous theoretical and experimental studies imply the existence of said reactions in nature.^89,139,452,453^ Moreover, a recent study suggests that the coherent relation between electrons is important in redox reactions involving transfer of multiple-electrons.^430^ This subject is in its infancy and work must be performed for understanding and establishing the effect for successful utilization in chemistry.

### 6.4 Role of CISS in Biology

Biomolecules and biopolymers in living organisms are largely homochiral, i.e., they appear almost exclusively in one enantiomeric form. Many workers have considered the origin of homochirality and its function; however, it remains an open question. Given its recent discovery, CISS has not been part of this scientific conversation; however, new experiments, which are described below, argue that it should be.

#### 6.4.1 Biological Redox Processes

The study of bioenergetics is the study of protein and substrate redox chemistry. Although proteins may at first seem an odd choice for redox chemistry, their amino acid properties and organization, which can significantly impact local cofactor environments, provide a way to control the energetics of redox reactions. Yet, these benefits do not require homochirality. The benefits arising from CISS do provide a fundamental rationale for nature to choose homochiral biopolymers, i.e., proteins for electron transfer. Numerous *in vitro* studies show that electron transfer in proteins, and their complexes, are spin polarized (see Section 3.1.2). Recent protein voltammetry studies of cytochrome *c*, immobilized on chiral tripeptide monolayer films, reveal the importance of the electron spin and the film’s homochirality on electron transfer kinetics.^58^ This study shows rate constant asymmetries as large as 60% and reveal marked differences in the average electron transfer rate constant for homochiral assemblies, in which the peptide and protein possess the same enantiomeric form, compared to heterochiral assemblies, where the handedness of the peptide layer is opposite to that of the protein, or is heterochiral itself. Because of the CISS effect and its resultant coupling of the linear momentum of the electron to its spin, the backscattering of electrons (which would require both electron-vibration coupling and a spin flip) is inhibited so that the fidelity of electron transfer over long ranges is facilitated.

#### 6.4.2 Role of Electron Spin on Protein Stability

A recent study has examined the importance of spin-exchange interactions on protein stability. After luciferase enzymes were adsorbed onto paramagnetic and ferromagnetic nanoparticles in solution, their denaturation upon addition of urea was examined.^232^ The enzymes structural stability was assessed using two methods: bioluminescence measurements, which monitored the activity of the Luciferase enzyme, and fast spectroscopy, which detected the distance between two chromophores implanted at the termini of a barnase core. For both measurements, interactions with magnetic materials altered the structural and functional resiliency of the natively folded proteins, showing greater stability on ferromagnetic surfaces than on paramagnetic surfaces, under mild denaturing conditions. The phenomenon was attributed to differences in the spin-exchange interactions involved in the magnetic imprinting properties of each type of nanoparticle and was supported by additional measurements on proteins at macroscopic magnetic surfaces. The results imply a link between internal spin-exchange interactions in a folded protein and its structural and functional integrity on magnetic surfaces; or more broadly, spin-exchange interactions should be considered as additional factors governing protein structure.

#### 6.4.3 Biomolecular Interactions and Recognition

Molecular interactions are essential in biology; however, understanding the strength of the interactions, and/or their specificity, typically relies upon a more general knowledge of the thermodynamics, dynamics, and structural components of the interacting species.^454,455^ Commonly, however, the bioaffinity that is measured in recognition processes is higher than those calculated by available methods.^456−459^ Using spin-exchange microscopy methods, a direct measurement of the interaction force between two chiral peptides showed a difference in force of ∼70 pN between homochiral and heterochiral peptide–peptide interactions.^116^ These findings were further supported by calculations using a simple theoretical model, which found that the spin-mediated interactions among peptides in close proximity was stronger than that of hydrogen bonding. Note that a model with better quantitative agreement to the data was achieved by incorporating dispersion interactions and spin-exchange interactions.^460^ EQCM methods have also been used to probe the effects of electron spin on biomolecular exchange interactions, showing that both the thermodynamic driving force as well as the dynamics for adsorption are affected.^222^ Interestingly, the enantiospecificity of the adsorption did not correlate with the handedness of the interacting substituents, but instead with the sign of the Cotton effect in the circular dichroism spectra associated with the interacting moiety. Collectively, these studies imply that the CISS effect can be just as important in biomolecular interactions and recognition events as traditional stereoisomeric and thermodynamic considerations.

#### 6.4.4 Allosteric Interactions

The transfer of information through biomolecules to induce binding or initiate reactions at remote sites, is a defining principle in biological chemistry and chemical biology.^461^ Yet, the fundamental mechanism(s) underlying information transfer through the several nanometers typical for cell membranes and proteins remains an open question. Protein function may be modulated by the binding of a small ligand or another protein, a familiar phenomenon termed allostery. Studies show that allostery can be mediated by a conformational change or by a change in a protein’s dynamics.^462−465^

Because modulation of a protein’s polarizability can affect its function, spin effects can become important. It was recently demonstrated that charge redistribution, and hence spin polarizations, affect allostery.^22^ In subsequent work, it was shown that phototriggered charge injection from a site-specific ruthenium photosensitizer into the protein phosphoglycerate kinase (PGK) increases its binding with an antibody by 2-fold and suppresses the enzymatic activity of PGK by a factor as large as three.^466^ Moreover, these responses are elicited by excitation with left (but not right) circularly polarized light, i.e., injected electrons spin matters, presumably because of spin-filtering by the protein’s chiral structure. This work reveals the possibility of controlling a protein’s binding and enzymatic activity through circular polarized light and/or by attaching chiral entities at a point remote to the protein’s active site.

#### 6.4.5 Origin of Life

The origin of symmetry breaking and the rise of homochiral organisms from a “primordial chemical soup” has long intrigued scientists. Because of the prevalence of amino acids and DNA in early CISS studies, the idea that the electron spin could affect biological processes is long-standing.^467^ The discovery that the electron spin can act as a chiral bias to enantiospecifically facilitate chemical reactions,^24,431^ however, has brought forth new deterministic hypotheses for the emergence and persistence of homochirality in Nature. For instance, Ozturk and Sasselov proposed that CISS could act as a symmetry breaking agent in cyanosulfidic prebiotic chemistry, which is hypothesized to give rise to some of life’s most important molecular building blocks.^468^ Primordial reactions such as these are conjectured to occur in shallow lake basins known to contain magnetite and have previously been identified as favorable geological locations to facilitate the origin of life.^469,470^

It is important to note that a large enantiomeric excess in initial reactions is not necessary to eventually achieve homochirality, multiple autocatalytic^471−473^ and nonlinear processes^474,475^ could occur that increase enantiopurity over time. In addition, CISS-mediated processes could also act to reinforce and propagate homochirality in biology. Studies have shown that spin-exchange interactions between chiral RNA precursors and magnetized magnetite can direct enantiospecific crystallization; achieving homochirality, i.e., 100% enantiomeric excess, from a racemic mixture in two steps.^394^ Moreover, studies have shown that spin alignment in homochiral assemblies affords more efficient energy transduction than that in heterochiral analogs, providing another rationale for Nature’s preference to be homochiral.^58^

#### 6.4.6 Future Directions

The emergence of life on earth is reported to date back as far as ∼3.7 billion years,^476^ and Nature has undergone considerable evolutionary change. Biology, as we know today, manifests highly organized structures on multiple length scales that gives rise to elegant functions. As such, leaning on life’s years of evolutionary optimization in physical and chemical processes to provide innovation and solutions for modern applications can be advantageous and is referred to as biomimicry.^477^ A greater understanding of the intricacies that define biological processes is thus paramount, and the discovery of CISS may help to further elucidate subtle features in biology, as well as improve existing biotechnologies. For instance, incorporation of spin polarization in bioelectronic mimetics may increase the specificity and sensitivity of biorecognition elements in sensors. Indeed, CISS-based sensing platforms are beginning to be developed.^59,61,105,478,479^ Moreover, the living cell can be viewed as a miniaturized information processor or computer; cells input information through intermolecular interactions, use proteins for intracellular communication, and store information as DNA. The cell performs these tasks more effectively than conventional computers.^480^ Does the spin information afforded through CISS contribute to this efficiency?

## 7 Critical Assessment of the Field

The field of CISS has grown rapidly in the past decade, advancing in many directions. The basic CISS observation, the presence of different electron currents through chiral structures when one of the contacts is magnetic and the magnetization is switched, has been observed by a variety of research groups worldwide. In addition, the CISS effect is observed over a large range of temperatures and manifests for single molecules, for monolayers, for thin films, and in bulk crystals. Theoretically, several viable mechanisms have been proposed to explain these phenomena, and an important emphasis in the short term will be to validate a mechanism experimentally—be it one of those proposed or some combination thereof.

A clear discrepancy exists between the experimental state of the field and the theoretical one. Experimentalists have a fair ability to predict, qualitatively, what to expect from their experimental setups and are using this ability to focus their studies on addressing questions that can support the theory developments, exploring the implications of CISS to other fields of study, or developing CISS-related applications. Basic questions like the effect of the substrate and of a chiral molecule’s SOC on the spin selectivity, the temperature dependence of CISS, the role of spin currents and angular momentum currents, and quantum CISS effects are now being studied. Other experimental efforts are examining the role of CISS in other research domains, including spin-controlled chemistry, biological processes, spintronics, and quantum information.

The situation is very different when one considers the theoretical efforts to explore the mechanism of CISS. It is now clear that all attempts to obtain quantitative agreement between calculations and experiments by using one electron models have failed. Theoretical approaches that go beyond the single electron Hamiltonian are being developed and show promise. They can reproduce major portions of the experiments and solve some of the issues raised in relation to time reversal symmetry and the Onsager principle. A major challenge for theory is to develop *ab initio* methods that include these “beyond single electron” concepts in order to open the way toward predicting experimental results in advance. Concepts that are important to include in theoretical models are the electrons’ spin and charge polarization, phonons/vibrations, and wave function entanglement.

## 8 Concluding Remarks and Future Outlook

Because the linkage of electron spin and the chiral symmetry of matter was not appreciated during the 20th century, much of our knowledge is built on information about matter that did not account for CISS and is often mute on its role. Investigations into CISS promise new insights for various fields, from chemistry and biology to physics. Many of the fundamental studies are providing a better understanding of CISS; however, these findings continue to open new avenues of study in other areas, e.g., emergent magnetism (Section 2.3.2), superconductivity spintronics (Section 6.1.3), and the origin of homochirality in biology (Section 6.4.5). At this stage of research, the ramifications of CISS cannot be fully charted. For instance, the ability to use the electron’s spin as a “chiral reagent” was demonstrated, but we are far from being able to achieve the goal of replacing methods for asymmetric catalysis by spin-polarized electron sources. This subject has enormous scientific and industrial potential, and effort should be devoted to its exploration. In addition, the role of CISS in molecular biology and its promise for developing new methods for controlling protein activity could be profound. Preliminary studies of CISS in biology have been conducted when the systems are anchored to ferromagnetic substrates, and it will be important to extend this work to more realistic conditions and to studies *invivo*. Molecules are “quantum devices”, and although it is natural to explore the possible role of chiral molecules in quantum information science (QIS), this field is nascent. The ability to create materials that show quantum properties at room temperature is very appealing for QIS, and CISS offers a new approach to this end. Discovered in 1999, CISS remains a scientific adolescent whose promise is high, but remains to be realized.

## References

[ref1] MacDermott A.J.; Barron L.D.; Brack A.; Buhse T.; Drake A.F.; Emery R.; Gottarelli G.; Greenberg J.M.; Haberle R.; Hegstrom R.A.; Hobbs K.; Kondepudi D.K.; McKay C.; Moorbath S.; Raulin F.; Sandford M.; Schwartzman D.W.; Thiemann W.H.-P.; Tranter G.E.; Zarnecki J.C. Homochirality as the Signature of Life: the SETH Cigar. Planet. Space Sci. 1996, 44, 1441–1446. 10.1016/S0032-0633(96)00057-8. 11541123

[ref2] Meierhenrich U. Amino Acids and the Asymmetry of Life: Caught in the Act of Formation; Springer. 2008.

[ref3] Xiao W.; Ernst K.-H.; Palotas K.; Zhang Y.; Bruyer E.; Peng L.; Greber T.; Hofer W. A.; Scott L. T.; Fasel R. Microscopic Origin of Chiral Shape Induction in Achiral Crystals. Nat. Chem. 2016, 8, 326–330. 10.1038/nchem.2449. 27001727

[ref4] Lee T. D.; Yang C. N. Question of Parity Conservation in Weak Interactions. Phys. Rev. 1956, 104, 254–258. 10.1103/PhysRev.104.254.

[ref5] Ray K.; Ananthavel S. P.; Waldeck D. H.; Naaman R. Asymmetric Scattering of Polarized Electrons by Organized Organic Films of Chiral Molecules. Science 1999, 283, 814–6. 10.1126/science.283.5403.814. 9933157

[ref6] Carmeli I.; Gefen Z.; Vager Z.; Naaman R. Alternation Between Modes of Electron Transmission through Organized Organic Layers. Phys. Rev. B 2003, 68, 115418 10.1103/PhysRevB.68.115418.

[ref7] Ray S. G.; Daube S. S.; Leitus G.; Vager Z.; Naaman R. Chirality-induced Spin-selective Properties of Self-assembled Monolayers of DNA on Gold. Phys. Rev. Lett. 2006, 96, 036101 10.1103/PhysRevLett.96.036101. 16486734

[ref8] Carmeli I.; Skakalova V.; Naaman R.; Vager Z. Magnetization of Chiral Monolayers of Polypeptide: a Possible Source of Magnetism in Some Biological Membranes. Angew. Chem., Int. Ed. Engl. 2002, 41, 761–4. 10.1002/1521-3773(20020301)41:5<761::AID-ANIE761>3.0.CO;2-Z. 12491328

[ref9] Wei J. J.; Schafmeister C.; Bird G.; Paul A.; Naaman R.; Waldeck D. H. Molecular Chirality and Charge Transfer Through Self-assembled Scaffold Monolayers. J. Phys. Chem. B 2006, 110, 1301–8. 10.1021/jp055145c. 16471678

[ref10] Xie Z. T.; Markus T. Z.; Cohen S. R.; Vager Z.; Gutierrez R.; Naaman R. Spin Specific Electron Conduction through DNA Oligomers. Nano Lett. 2011, 11, 4652–4655. 10.1021/nl2021637. 21961931

[ref11] Aragones A. C.; Medina E.; Ferrer-Huerta M.; Gimeno N.; Teixido M.; Palma J. L.; Tao N.; Ugalde J. M.; Giralt E.; Diez-Perez I.; Mujica V. Measuring the Spin-Polarization Power of a Single Chiral Molecule. Small 2017, 13, 1602519 10.1002/smll.201602519. 27753200

[ref12] Göhler B.; Hamelbeck V.; Markus T. Z.; Kettner M.; Hanne G. F.; Vager Z.; Naaman R.; Zacharias H. Spin Selectivity in Electron Transmission through Self-assembled Monolayers of Double-stranded DNA. Science 2011, 331, 894–7. 10.1126/science.1199339. 21330541

[ref13] Naaman R.; Waldeck D. H. Chiral-Induced Spin Selectivity Effect. J. Phys. Chem. Lett. 2012, 3 (16), 2178–2187. 10.1021/jz300793y. 26295768

[ref14] Evers F.; Aharony A.; Bar-Gill N.; Entin-Wohlman O.; Hedegard P.; Hod O.; Jelinek P.; Kamieniarz G.; Lemeshko M.; Michaeli K.; Mujica V.; Naaman R.; Paltiel Y.; Refaely-Abramson S.; Tal O.; Thijssen J.; Thoss M.; van Ruitenbeek J. M.; Venkataraman L.; Waldeck D. H.; Yan B.; Kronik L. Theory of Chirality Induced Spin Selectivity: Progress and Challenges. Adv. Mater. 2022, 34, e2106629 10.1002/adma.202106629. 35064943

[ref15] Kettner M.; Göhler B.; Zacharias H.; Mishra D.; Kiran V.; Naaman R.; Fontanesi C.; Waldeck D. H.; Sek S.; Pawlowski J.; Juhaniewicz J. Spin Filtering in Electron Transport Through Chiral Oligopeptides. J. Phys. Chem. C 2015, 119, 14542–14547. 10.1021/jp509974z.

[ref16] Mazin I. I. How to Define and Calculate the Degree of Spin Polarization in Ferromagnets. Phys. Rev. Lett. 1999, 83, 1427–1430. 10.1103/PhysRevLett.83.1427.

[ref17] Liu T.; Weiss P. S. Spin Polarization in Transport Studies of Chirality-Induced Spin Selectivity. ACS Nano 2023, 17, 19502–19507. 10.1021/acsnano.3c06133. 37793070

[ref18] Naaman R.; Paltiel Y.; Waldeck D. H. Chiral Induced Spin Selectivity Gives a New Twist on Spin-Control in Chemistry. Acc. Chem. Res. 2020, 53, 2659–2667. 10.1021/acs.accounts.0c00485. 33044813 PMC7676290

[ref19] Ghosh S.; Mishra S.; Avigad E.; Bloom B. P.; Baczewski L. T.; Yochelis S.; Paltiel Y.; Naaman R.; Waldeck D. H. Effect of Chiral Molecules on the Electron’s Spin Wavefunction at Interfaces. J. Phys. Chem. Lett. 2020, 11, 1550–1557. 10.1021/acs.jpclett.9b03487. 32013436 PMC7307953

[ref20] Kumar A.; Capua E.; Kesharwani M. K.; Martin J. M.; Sitbon E.; Waldeck D. H.; Naaman R. Chirality-induced Spin Polarization Places Symmetry Constraints on Biomolecular Interactions. Proc. Natl. Acad. Sci. U.S.A. 2017, 114, 2474–2478. 10.1073/pnas.1611467114. 28228525 PMC5347616

[ref21] Banerjee-Ghosh K.; Ben Dor O.; Tassinari F.; Capua E.; Yochelis S.; Capua A.; Yang S. H.; Parkin S. S. P.; Sarkar S.; Kronik L.; Baczewski L. T.; Naaman R.; Paltiel Y. Separation of Enantiomers by their Enantiospecific Interaction with Achiral Magnetic Substrates. Science 2018, 360, 1331–1334. 10.1126/science.aar4265. 29748324

[ref22] Banerjee-Ghosh K.; Ghosh S.; Mazal H.; Riven I.; Haran G.; Naaman R. Long-Range Charge Reorganization as an Allosteric Control Signal in Proteins. J. Am. Chem. Soc. 2020, 142, 20456–20462. 10.1021/jacs.0c10105. 33211484 PMC7735699

[ref23] Lu Y.; Bloom B. P.; Qian S.; Waldeck D. H. Enantiospecificity of Cysteine Adsorption on a Ferromagnetic Surface: Is It Kinetically or Thermodynamically Controlled?. J. Phys. Chem. Lett. 2021, 12, 7854–7858. 10.1021/acs.jpclett.1c02087. 34380316

[ref24] Metzger T. S.; Mishra S.; Bloom B. P.; Goren N.; Neubauer A.; Shmul G.; Wei J.; Yochelis S.; Tassinari F.; Fontanesi C.; Waldeck D. H.; Paltiel Y.; Naaman R. The Electron Spin as a Chiral Reagent. Angew. Chem., Int. Ed. Engl. 2020, 59, 1653–1658. 10.1002/anie.201911400. 31621990

[ref25] Radetic M.; Gellman A. J. Enantiomer Adsorption in an Applied Magnetic Field: D- and L-Aspartic Acid on Ni(100). Isr. J. Chem. 2022, 62, e202200028 10.1002/ijch.202200028.

[ref26] Privitera A.; Macaluso E.; Chiesa A.; Gabbani A.; Faccio D.; Giuri D.; Briganti M.; Giaconi N.; Santanni F.; Jarmouni N.; Poggini L.; Mannini M.; Chiesa M.; Tomasini C.; Pineider F.; Salvadori E.; Carretta S.; Sessoli R. Direct Detection of Spin Polarization in Photoinduced Charge Transfer through a Chiral Bridge. Chem. Sci. 2022, 13, 12208–12218. 10.1039/D2SC03712B. 36349110 PMC9601404

[ref27] Badala Viswanatha C.; Stockl J.; Arnoldi B.; Becker S.; Aeschlimann M.; Stadtmuller B. Vectorial Electron Spin Filtering by an All-Chiral Metal-Molecule Heterostructure. J. Phys. Chem. Lett. 2022, 13, 6244–6249. 10.1021/acs.jpclett.2c00983. 35771050 PMC9272820

[ref28] Kettner M.; Maslyuk V. V.; Nurenberg D.; Seibel J.; Gutierrez R.; Cuniberti G.; Ernst K. H.; Zacharias H. Chirality-Dependent Electron Spin Filtering by Molecular Monolayers of Helicenes. J. Phys. Chem. Lett. 2018, 9, 2025–2030. 10.1021/acs.jpclett.8b00208. 29618210

[ref29] Möllers P. V.; Göhler B.; Zacharias H. Chirality Induced Spin Selectivity-the Photoelectron View. Isr. J. Chem. 2022, 62, e202200062 10.1002/ijch.202200062.

[ref30] Abendroth J. M.; Cheung K. M.; Stemer D. M.; El Hadri M. S.; Zhao C.; Fullerton E. E.; Weiss P. S. Spin-Dependent Ionization of Chiral Molecular Films. J. Am. Chem. Soc. 2019, 141, 3863–3874. 10.1021/jacs.8b08421. 30734553 PMC6703823

[ref31] Stemer D. M.; Abendroth J. M.; Cheung K. M.; Ye M.; El Hadri M. S.; Fullerton E. E.; Weiss P. S. Differential Charging in Photoemission from Mercurated DNA Monolayers on Ferromagnetic Films. Nano Lett. 2020, 20, 1218–1225. 10.1021/acs.nanolett.9b04622. 31960675 PMC7058983

[ref32] Rosenberg R. A.; Mishra D.; Naaman R. Chiral Selective Chemistry Induced by Natural Selection of Spin-Polarized Electrons. Angew. Chem., Int. Ed. Engl. 2015, 54, 7295–8. 10.1002/anie.201501678. 25950284

[ref33] Robins J. L.; Celotta R. J.; Unguris J.; Pierce D. T.; Jonker B. T.; Prinz G. A. Domain Images of Ultrathin Fe Films on Ag(100). Appl. Phys. Lett. 1988, 52, 1918–1920. 10.1063/1.99616.

[ref34] Kurzawa R.; Kämper K. P.; Schmitt W.; Güntherodt G. Spin-resolved Photoemission Study of in situ Grown Epitaxial Fe Layers on W(110). Solid State Commun. 1986, 60, 777–780. 10.1016/0038-1098(86)90594-6.

[ref35] Abraham D. L.; Hopster H. Spin-polarized Electron-energy-loss Spectroscopy on Ni. Phys. Rev. Lett. 1989, 62, 1157–1160. 10.1103/PhysRevLett.62.1157. 10039591

[ref36] Pierce D. T.; Celotta R.; Wang G. C.; Unertl W.; Galejs A.; Kuyatt C.; Mielczarek S. The GaAs Spin Polarized Electron Source. Rev. Sci. Instrum. 1980, 51, 478–499. 10.1063/1.1136250.

[ref37] Baum G.; Fink M.; Raith W.; Steidl H.; Taborski J. Polarized Electron-impact Ionization of Metastable Helium. Phys. Rev. A 1989, 40, 6734–6736. 10.1103/PhysRevA.40.6734. 9902077

[ref38] Gay T. J.; Dunning F. Mott Electron Polarimetry. Rev. Sci. Instrum. 1992, 63, 1635–1651. 10.1063/1.1143371.

[ref39] Möllers P. V.; Wei J.; Salamon S.; Bartsch M.; Wende H.; Waldeck D. H.; Zacharias H. Spin-Polarized Photoemission from Chiral CuO Catalyst Thin Films. ACS Nano 2022, 16, 12145–12155. 10.1021/acsnano.2c02709. 35943911 PMC9413420

[ref40] Möllers P. V.; Ulku S.; Jayarathna D.; Tassinari F.; Nurenberg D.; Naaman R.; Achim C.; Zacharias H. Spin-selective Electron Tansmission through Self-assembled Monolayers of Double-stranded Peptide Nucleic Acid. Chirality 2021, 33, 93–102. 10.1002/chir.23290. 33400337

[ref41] Ghosh K. B.; Zhang W. Y.; Tassinari F.; Mastai Y.; Lidor-Shalev O.; Naaman R.; Möllers P.; Nurenberg D.; Zacharias H.; Wei J.; Wierzbinski E.; Waldeck D. H. Controlling Chemical Selectivity in Electrocatalysis with Chiral CuO-Coated Electrodes. J. Phys. Chem. C 2019, 123, 3024–3031. 10.1021/acs.jpcc.8b12027.

[ref42] Ko C.-H.; Zhu Q.; Bullard G.; Tassinari F.; Morisue M.; Naaman R.; Therien M. J. Electron Spin Polarization and Rectification Driven by Chiral Perylene Diimide-Based Nanodonuts. J. Phys. Chem. Lett. 2023, 14, 10271–10277. 10.1021/acs.jpclett.3c02722. 37939254

[ref43] Clever C.; Wierzbinski E.; Bloom B. P.; Lu Y. Y.; Grimm H. M.; Rao S. R.; Horne W. S.; Waldeck D. H. Benchmarking Chiral Induced Spin Selectivity Measurements - Towards Meaningful Comparisons of Chiral Biomolecule Spin Polarizations. Isr. J. Chem. 2022, 62, e202200045 10.1002/ijch.202200045.

[ref44] Carmeli I.; Leitus G.; Naaman R.; Reich S.; Vager Z. New Electronic and Magnetic Properties of Monolayers of Thiols on Gold. Isr. J. Chem. 2003, 43, 399–405. 10.1560/NTTG-64MP-VQWJ-M34U.

[ref45] Waldeck D. H.; Naaman R.; Paltiel Y. The Spin Selectivity Effect in Chiral Materials. APL Mater. 2021, 9, 040902 10.1063/5.0049150.

[ref46] Naaman R.; Paltiel Y.; Waldeck D. H. Chiral Molecules and the Spin Selectivity Effect. J. Phys. Chem. Lett. 2020, 11, 3660–3666. 10.1021/acs.jpclett.0c00474. 32298118 PMC7304900

[ref47] Safari M. R.; Matthes F.; Schneider C. M.; Ernst K.-H.; Bürgler D. E. Spin-Selective Electron Transport Through Single Chiral Molecules. Small 2023, 2308233 10.1002/smll.202308233. 38050945

[ref48] Safari M. R.; Matthes F.; Ernst K. H.; Burgler D. E.; Schneider C. M. Deposition of Chiral Heptahelicene Molecules on Ferromagnetic Co and Fe Thin-Film Substrates. Nanomater. 2022, 12, 3281 10.3390/nano12193281. PMC9565510 36234411

[ref49] Ortuño A. M.; Reiné P.; Álvarez de Cienfuegos L.; Márquez I. R.; Dednam W.; Lombardi E. B.; Palacios J. J.; Leary E.; Longhi G.; Mujica V.; Millán A.; González M. T.; Zotti L. A.; Miguel D.; Cuerva J. M. Chiral Single-Molecule Potentiometers Based on Stapled Ortho- oligo(phenylene)ethynylenes. Angew. Chem., Int. Ed. 2023, 62, e202218640 10.1002/anie.202218640. 36806838

[ref50] Ben Dor O.; Yochelis S.; Mathew S. P.; Naaman R.; Paltiel Y. A Chiral-based Magnetic Memory Device Without a Permanent Magnet. Nat. Commun. 2013, 4, 2256 10.1038/ncomms3256. 23922081 PMC3741643

[ref51] Naaman R.; Paltiel Y.; Waldeck D. H. Chiral Molecules and the Electron Spin. Nat. Rev. Chem. 2019, 3, 250–260. 10.1038/s41570-019-0087-1.

[ref52] Das T. K.; Tassinari F.; Naaman R.; Fransson J. Temperature-Dependent Chiral-Induced Spin Selectivity Effect: Experiments and Theory. J. Phys. Chem. C 2022, 126, 3257–3264. 10.1021/acs.jpcc.1c10550.

[ref53] Inui A.; Aoki R.; Nishiue Y.; Shiota K.; Kousaka Y.; Shishido H.; Hirobe D.; Suda M.; Ohe J. I.; Kishine J. I.; Yamamoto H. M.; Togawa Y. Chirality-Induced Spin-Polarized State of a Chiral Crystal CrNb_3_S_6_. Phys. Rev. Lett. 2020, 124, 166602 10.1103/PhysRevLett.124.166602. 32383920

[ref54] Liu T.; Wang X.; Wang H.; Shi G.; Gao F.; Feng H.; Deng H.; Hu L.; Lochner E.; Schlottmann P.; von Molnár S.; Li Y.; Zhao J.; Xiong P. Linear and Nonlinear Two-Terminal Spin-Valve Effect from Chirality-Induced Spin Selectivity. ACS Nano 2020, 14, 15983–15991. 10.1021/acsnano.0c07438. 33136367

[ref55] Mondal P. C.; Roy P.; Kim D.; Fullerton E. E.; Cohen H.; Naaman R. Photospintronics: Magnetic Field-Controlled Photoemission and Light-Controlled Spin Transport in Hybrid Chiral Oligopeptide-Nanoparticle Structures. Nano Lett. 2016, 16, 2806–11. 10.1021/acs.nanolett.6b00582. 27027885 PMC4834632

[ref56] Tassinari F.; Jayarathna D. R.; Kantor-Uriel N.; Davis K. L.; Varade V.; Achim C.; Naaman R. Chirality Dependent Charge Transfer Rate in Oligopeptides. Adv. Mater. 2018, 30, e1706423 10.1002/adma.201706423. 29611223

[ref57] Ben Dor O.; Yochelis S.; Radko A.; Vankayala K.; Capua E.; Capua A.; Yang S. H.; Baczewski L. T.; Parkin S. S.; Naaman R.; Paltiel Y. Magnetization Switching in Ferromagnets by Adsorbed Chiral Molecules without Current or External Magnetic Field. Nat. Commun. 2017, 8, 14567 10.1038/ncomms14567. 28230054 PMC5331337

[ref58] Wei J.; Bloom B. P.; Dunlap-Shohl W. A.; Clever C. B.; Rivas J. E.; Waldeck D. H. Examining the Effects of Homochirality for Electron Transfer in Protein Assemblies. J. Phys. Chem. B 2023, 127, 6462–6469. 10.1021/acs.jpcb.3c02913. 37463031 PMC10388353

[ref59] Bangruwa N.; Srivastava M.; Mishra D. CISS-Based Label-Free Novel Electrochemical Impedimetric Detection of UVC-Induced DNA Damage. ACS Omega 2022, 7, 37705–37713. 10.1021/acsomega.2c04659. 36312421 PMC9609074

[ref60] Bangruwa N.; Suryansh; Peralta M.; Gutierrez R.; Cuniberti G.; Mishra D. Sequence-controlled Chiral Induced Spin Selectivity Effect in ds-DNA. J. Chem. Phys. 2023, 159, 044702 10.1063/5.0157931. 37486052

[ref61] Bhartiya P. K.; Suryansh; Bangruwa N.; Srivastava M.; Mishra D. Light-Amplified CISS-Based Hybrid QD-DNA Impedimetric Device for DNA Hybridization Detection. Anal. Chem. 2023, 95, 3656–3665. 10.1021/acs.analchem.2c04608. 36749750

[ref62] Jedema F. J.; Heersche H. B.; Filip A. T.; Baselmans J. J.; van Wees B. J. Electrical Detection of Spin Precession in a Metallic Mesoscopic Spin Valve. Nature 2002, 416, 713–716. 10.1038/416713a. 11961548

[ref63] Lou X. H.; Adelmann C.; Crooker S. A.; Garlid E. S.; Zhang J.; Reddy K. S. M.; Flexner S. D.; Palmstrom C. J.; Crowell P. A. Electrical Detection of Spin Transport in Lateral Ferromagnet-Semiconductor Devices. Nat. Phys. 2007, 3, 197–202. 10.1038/nphys543.

[ref64] van ’t Erve O. M.; Friedman A. L.; Li C. H.; Robinson J. T.; Connell J.; Lauhon L. J.; Jonker B. T. Spin Transport and Hanle Effect in Silicon Nanowires using Graphene Tunnel Barriers. Nat. Commun. 2015, 6, 7541 10.1038/ncomms8541. 26089110

[ref65] Kim J.-I.; Liu T.; Kountouriotis K.; Lu J.; Yu X.; Adhikari Y.; von Molnár S.; Zhao J.; Xiong P. Direct Comparison of Three-terminal and Four-terminal Hanle Effects in the Persistent Photoconductor Al_0.3_Ga_0.7_As:Si. Phys. Rev. Mater. 2022, 6, 024603 10.1103/PhysRevMaterials.6.024603.

[ref66] Liu T.; Adhikari Y.; Wang H.; Hua Z.; Liu H.; Zhao J.; Xiong P. Hanle Effect without a Magnet in Chiral Molecular Junctions. Bull. Am. Phys. Soc. 2023, 68.

[ref67] Eckshtain-Levi M.; Capua E.; Refaely-Abramson S.; Sarkar S.; Gavrilov Y.; Mathew S. P.; Paltiel Y.; Levy Y.; Kronik L.; Naaman R. Cold Denaturation Induces Inversion of Dipole and Spin Transfer in Chiral Peptide Monolayers. Nat. Commun. 2016, 7, 10744 10.1038/ncomms10744. 26916536 PMC4773432

[ref68] Jungwirth T.; Wunderlich J.; Olejnik K. Spin Hall Effect Devices. Nat. Mater. 2012, 11, 382–90. 10.1038/nmat3279. 22522638

[ref69] Fontanesi C.; Capua E.; Paltiel Y.; Waldeck D. H.; Naaman R. Spin-Dependent Processes Measured without a Permanent Magnet. Adv. Mater. 2018, 30, e1707390 10.1002/adma.201707390. 29736985

[ref70] Pauw L. J. v. d. A Method of Measuring Specific Resistivity and Hall Effect of Discs of Arbitrary Shape. Philips Res. Rep 1958, 13, 1–9.

[ref71] Wang S. X.; Chang H. R.; Zhou J. H. RKKY Interaction in Three-dimensional Electron Gases with Linear Spin-orbit Coupling. Phys. Rev. B 2017, 96, 115204 10.1103/PhysRevB.96.115204.

[ref72] Kumar A.; Capua E.; Fontanesi C.; Carmieli R.; Naaman R. Injection of Spin-Polarized Electrons into a AlGaN/GaN Device from an Electrochemical Cell: Evidence for an Extremely Long Spin Lifetime. ACS Nano 2018, 12, 3892–3897. 10.1021/acsnano.8b01347. 29617105

[ref73] Mondal P. C.; Fontanesi C.; Waldeck D. H.; Naaman R. Spin-Dependent Transport through Chiral Molecules Studied by Spin-Dependent Electrochemistry. Acc. Chem. Res. 2016, 49, 2560–2568. 10.1021/acs.accounts.6b00446. 27797176 PMC5112609

[ref74] Kumar A.; Capua E.; Vankayala K.; Fontanesi C.; Naaman R. Magnetless Device for Conducting Three-Dimensional Spin-Specific Electrochemistry. Angew. Chem., Int. Ed. Engl. 2017, 56, 14587–14590. 10.1002/anie.201708829. 28960865

[ref75] Nagaosa N.; Sinova J.; Onoda S.; MacDonald A. H.; Ong N. P. Anomalous Hall Effect. Rev. Mod. Phys. 2010, 82, 1539–1592. 10.1103/RevModPhys.82.1539.

[ref76] Miyasato T.; Abe N.; Fujii T.; Asamitsu A.; Onoda S.; Onose Y.; Nagaosa N.; Tokura Y. Crossover Behavior of the Anomalous Hall Effect and Anomalous Nernst Effect in Itinerant Ferromagnets. Phys. Rev. Lett. 2007, 99, 086602 10.1103/PhysRevLett.99.086602. 17930968

[ref77] Smolinsky E. Z. B.; Neubauer A.; Kumar A.; Yochelis S.; Capua E.; Carmieli R.; Paltiel Y.; Naaman R.; Michaeli K. Electric Field-Controlled Magnetization in GaAs/AlGaAs Heterostructures-Chiral Organic Molecules Hybrids. J. Phys. Chem. Lett. 2019, 10, 1139–1145. 10.1021/acs.jpclett.9b00092. 30785758

[ref78] Ziv A.; Saha A.; Alpern H.; Sukenik N.; Baczewski L. T.; Yochelis S.; Reches M.; Paltiel Y. AFM-Based Spin-Exchange Microscopy Using Chiral Molecules. Adv. Mater. 2019, 31, 1904206 10.1002/adma.201904206. 31423697

[ref79] Nonnenmacher M.; O'Boyle M. P.; Wickramasinghe H. K. Kelvin Probe Force Microscopy. Appl. Phys. Lett. 1991, 58, 2921–2923. 10.1063/1.105227.

[ref80] Abendroth J. M.; Nakatsuka N.; Ye M.; Kim D.; Fullerton E. E.; Andrews A. M.; Weiss P. S. Analyzing Spin Selectivity in DNA-Mediated Charge Transfer via Fluorescence Microscopy. ACS Nano 2017, 11, 7516–7526. 10.1021/acsnano.7b04165. 28672111

[ref81] Roy P.; Kantor-Uriel N.; Mishra D.; Dutta S.; Friedman N.; Sheves M.; Naaman R. Spin-Controlled Photoluminescence in Hybrid Nanoparticles Purple Membrane System. ACS Nano 2016, 10, 4525–4531. 10.1021/acsnano.6b00333. 27018195 PMC4850504

[ref82] Bloom B. P.; Graff B. M.; Ghosh S.; Beratan D. N.; Waldeck D. H. Chirality Control of Electron Transfer in Quantum Dot Assemblies. J. Am. Chem. Soc. 2017, 139, 9038–9043. 10.1021/jacs.7b04639. 28609095

[ref83] Buckingham A. D.; Fischer P. Direct Chiral Discrimination in NMR Spectroscopy. Chem. Phys. 2006, 324, 111–116. 10.1016/j.chemphys.2005.10.009.

[ref84] Buckingham A. D. Communication: Permanent Dipoles Contribute to Electric Polarization in Chiral NMR Spectra. J. Chem. Phys. 2014, 140, 011103 10.1063/1.4859256. 24410214

[ref85] Santos J. I.; Rivilla I.; Cossio F. P.; Matxain J. M.; Grzelczak M.; Mazinani S. K. S.; Ugalde J. M.; Mujica V. Chirality-Induced Electron Spin Polarization and Enantiospecific Response in Solid-State Cross-Polarization Nuclear Magnetic Resonance. ACS Nano 2018, 12, 11426–11433. 10.1021/acsnano.8b06467. 30407788

[ref86] San Sebastian E.; Cepeda J.; Huizi-Rayo U.; Terenzi A.; Finkelstein-Shapiro D.; Padro D.; Santos J. I.; Matxain J. M.; Ugalde J. M.; Mujica V. Enantiospecific Response in Cross-Polarization Solid-State Nuclear Magnetic Resonance of Optically Active Metal Organic Frameworks. J. Am. Chem. Soc. 2020, 142, 17989–17996. 10.1021/jacs.0c04537. 32941015

[ref87] Blumenschein F.; Tamski M.; Roussel C.; Smolinsky E. Z. B.; Tassinari F.; Naaman R.; Ansermet J. P. Spin-dependent Charge Tansfer at Chiral Electrodes Probed by Magnetic Resonance. Phys. Chem. Chem. Phys. 2020, 22, 997–1002. 10.1039/C9CP04681J. 31691683

[ref88] Chiesa A.; Chizzini M.; Garlatti E.; Salvadori E.; Tacchino F.; Santini P.; Tavernelli I.; Bittl R.; Chiesa M.; Sessoli R.; Carretta S. Assessing the Nature of Chiral-Induced Spin Selectivity by Magnetic Resonance. J. Phys. Chem. Lett. 2021, 12, 6341–6347. 10.1021/acs.jpclett.1c01447. 34228926 PMC8397348

[ref89] Luo J. T.; Hore P. J. Chiral-induced Spin Selectivity in the Formation and Recombination of Radical Pairs: Cryptochrome Magnetoreception and EPR Detection. New J. Phys. 2021, 23, 043032 10.1088/1367-2630/abed0b.

[ref90] Volker L. A.; Herb K.; Janitz E.; Degen C. L.; Abendroth J. M. Toward Quantum Sensing of Chiral Induced Spin Selectivity: Probing Donor-bridge-acceptor Molecules with NV Centers in Diamond. J. Chem. Phys. 2023, 158, 161103 10.1063/5.0145466. 37093150

[ref91] Eckvahl H. J.; Tcyrulnikov N. A.; Chiesa A.; Bradley J. M.; Young R. M.; Carretta S.; Krzyaniak M. D.; Wasielewski M. R. Direct Observation of Chirality-induced Spin Selectivity in Electron Donor-acceptor molecules. Science 2023, 382, 197–201. 10.1126/science.adj5328. 37824648

[ref92] Koplovitz G.; Leitus G.; Ghosh S.; Bloom B. P.; Yochelis S.; Rotem D.; Vischio F.; Striccoli M.; Fanizza E.; Naaman R.; Waldeck D. H.; Porath D.; Paltiel Y. Single Domain 10 nm Ferromagnetism Imprinted on Superparamagnetic Nanoparticles Using Chiral Molecules. Small 2019, 15, e1804557 10.1002/smll.201804557. 30462882

[ref93] Ozeri M.; Devidas T. R.; Alpern H.; Persky E.; Bjorlig A. V.; Sukenik N.; Yochelis S.; Di Bernardo A.; Kalisky B.; Millo O.; Paltiel Y. Scanning SQUID Imaging of Reduced Superconductivity Due to the Effect of Chiral Molecule Islands Adsorbed on Nb. Adv. Mater. Interfaces 2023, 10, 2201899 10.1002/admi.202201899.

[ref94] Stephens P. Magnetic Circular Dichroism. Annu. Rev. Phys. Chem. 1974, 25, 201–232. 10.1146/annurev.pc.25.100174.001221.

[ref95] Bai T.; Ai J.; Duan Y.; Han L.; Che S. Spin Selectivity of Chiral Mesostructured Iron Oxides with Different Magnetisms. Small 2022, 18, e2104509 10.1002/smll.202104509. 35098648

[ref96] Ding K.; Ai J.; Chen H.; Qu Z. B.; Liu P. Z.; Han L.; Che S. A.; Duan Y. Y. Spin Selectivity of Chiral Mesostructured Diamagnetic BiOBr Films. Nano Res. 2023, 16, 11444–11449. 10.1007/s12274-023-5866-9.

[ref97] Huang Z.; Bloom B. P.; Ni X.; Georgieva Z. N.; Marciesky M.; Vetter E.; Liu F.; Waldeck D. H.; Sun D. Magneto-Optical Detection of Photoinduced Magnetism via Chirality-Induced Spin Selectivity in 2D Chiral Hybrid Organic-Inorganic Perovskites. ACS Nano 2020, 14, 10370–10375. 10.1021/acsnano.0c04017. 32678570

[ref98] Kim K.; Vetter E.; Yan L.; Yang C.; Wang Z.; Sun R.; Yang Y.; Comstock A. H.; Li X.; Zhou J.; Zhang L.; You W.; Sun D.; Liu J. Chiral-phonon-activated Spin Seebeck Effect. Nat. Mater. 2023, 22, 322–328. 10.1038/s41563-023-01473-9. 36781951

[ref99] Meirzada I.; Sukenik N.; Haim G.; Yochelis S.; Baczewski L. T.; Paltiel Y.; Bar-Gill N. Long-Time-Scale Magnetization Ordering Induced by an Adsorbed Chiral Monolayer on Ferromagnets. ACS Nano 2021, 15, 5574–5579. 10.1021/acsnano.1c00455. 33591720

[ref100] Uchida K.; Takahashi S.; Harii K.; Ieda J.; Koshibae W.; Ando K.; Maekawa S.; Saitoh E. Observation of the Spin Seebeck Effect. Nature 2008, 455, 778–81. 10.1038/nature07321. 18843364

[ref101] Yu T.; Blanter Y. M.; Bauer G. E. W. Chiral Pumping of Spin Waves. Phys. Rev. Lett. 2019, 123, 247202 10.1103/PhysRevLett.123.247202. 31922821

[ref102] Zwang T. J.; Hurlimann S.; Hill M. G.; Barton J. K. Helix-Dependent Spin Filtering through the DNA Duplex. J. Am. Chem. Soc. 2016, 138, 15551–15554. 10.1021/jacs.6b10538. 27934017 PMC5175457

[ref103] Rosenberg R. A.; Symonds J. M.; Kalyanaraman V.; Markus T.; Orlando T. M.; Naaman R.; Medina E. A.; Lopez F. A.; Mujica V. Kinetic Energy Dependence of Spin Filtering of Electrons Transmitted through Organized Layers of DNA. J. Phys. Chem. C 2013, 117, 22307–22313. 10.1021/jp402387y.

[ref104] Mishra S.; Mondal A. K.; Pal S.; Das T. K.; Smolinsky E. Z. B.; Siligardi G.; Naaman R. Length-Dependent Electron Spin Polarization in Oligopeptides and DNA. J. Phys. Chem. C 2020, 124, 10776–10782. 10.1021/acs.jpcc.0c02291.

[ref105] Bangruwa N.; Bhartiya P. K.; Mishra D. A Novel Spin-based Label-free Electrochemical DNA Hybridization Biosensor and its Applications for Dengue Virus Detection. Sens. Actuators B Chem. 2023, 382, 133447 10.1016/j.snb.2023.133447.

[ref106] Pal C.; Majumder S. Manipulating Electron-spin Polarization using Cysteine-DNA Chiral Conjugates. J. Chem. Phys. 2022, 156, 164704 10.1063/5.0088346. 35490028

[ref107] Bangruwa N.; Srivastava M.; Mishra D. Radiation-Induced Effect on Spin-Selective Electron Transfer through Self-Assembled Monolayers of ds-DNA. Magnetochemistry 2021, 7, 98 10.3390/magnetochemistry7070098.

[ref108] Deng L.; Bhat I. H.; Guo A.-M. Spin-selectivity Effect of G-quadruplex DNA Molecules. J. Chem. Phys. 2023, 158, 244116 10.1063/5.0156389. 37377158

[ref109] Mishra S.; Poonia V. S.; Fontanesi C.; Naaman R.; Fleming A. M.; Burrows C. J. Effect of Oxidative Damage on Charge and Spin Transport in DNA. J. Am. Chem. Soc.y 2019, 141, 123–126. 10.1021/jacs.8b12014. 30541275

[ref110] Zhu Q.; Kapon Y.; Fleming A. M.; Mishra S.; Santra K.; Tassinari F.; Cohen S. R.; Das T. K.; Sang Y.; Bhowmick D. K.; Burrows C. J.; Paltiel Y.; Naaman R. The Role of Electrons’ Spin in DNA Oxidative Damage Recognition. Cell Rep. Phys. Sci. 2022, 3, 101157 10.1016/j.xcrp.2022.101157.

[ref111] Santra K.; Lu Y.; Waldeck D. H.; Naaman R. Spin Selectivity Damage Dependence of Adsorption of dsDNA on Ferromagnets. J. Phys. Chem. B 2023, 127, 2344–2350. 10.1021/acs.jpcb.2c08820. 36888909 PMC10041612

[ref112] Sharma A.; Matthes P.; Soldatov I.; Arekapudi S. S. P. K.; Böhm B.; Lindner M.; Selyshchev O.; Thi Ngoc Ha N.; Mehring M.; Tegenkamp C.; Schulz S. E.; Zahn D. R. T.; Paltiel Y.; Hellwig O.; Salvan G. Control of Magneto-optical Properties of Cobalt-layers by Adsorption of α-helical Polyalanine Self-assembled Monolayers. J. Mater. Chem. C 2020, 8, 11822–11829. 10.1039/D0TC02734K.

[ref113] Nguyen T. N. H.; Rasabathina L.; Hellwig O.; Sharma A.; Salvan G.; Yochelis S.; Paltiel Y.; Baczewski L. T.; Tegenkamp C. Cooperative Effect of Electron Spin Polarization in Chiral Molecules Studied with Non-Spin-Polarized Scanning Tunneling Microscopy. ACS Appl. Mater. Interfaces 2022, 14, 38013–38020. 10.1021/acsami.2c08668. 35960822

[ref114] Sukenik N.; Tassinari F.; Yochelis S.; Millo O.; Baczewski L. T.; Paltiel Y. Correlation between Ferromagnetic Layer Easy Axis and the Tilt Angle of Self Assembled Chiral Molecules. Molecules 2020, 25, 6036 10.3390/molecules25246036. 33419359 PMC7765850

[ref115] Kashiwagi K.; Tassinari F.; Haraguchi T.; Banerjee-Gosh K.; Akitsu T.; Naaman R. Electron Transfer via Helical Oligopeptide to Laccase Including Chiral Schiff Base Copper Mediators. Symmetry 2020, 12, 808 10.3390/sym12050808.

[ref116] Kapon Y.; Saha A.; Duanis-Assaf T.; Stuyver T.; Ziv A.; Metzger T.; Yochelis S.; Shaik S.; Naaman R.; Reches M.; Paltiel Y. Evidence for New Enantiospecific Interaction Force in Chiral Biomolecules. Chem. 2021, 7, 2787–2799. 10.1016/j.chempr.2021.08.002.

[ref117] Zhang W. Y.; Banerjee-Ghosh K.; Tassinari F.; Naaman R. Enhanced Electrochemical Water Splitting with Chiral Molecule-Coated Fe_3_O_4_ Nanoparticles. ACS Energy Lett. 2018, 3, 2308–2313. 10.1021/acsenergylett.8b01454.

[ref118] Ben Dor O.; Morali N.; Yochelis S.; Baczewski L. T.; Paltiel Y. Local Light-induced Magnetization using Nanodots and Chiral Molecules. Nano Lett. 2014, 14, 6042–6049. 10.1021/nl502391t. 25313442

[ref119] Alpern H.; Yavilberg K.; Dvir T.; Sukenik N.; Klang M.; Yochelis S.; Cohen H.; Grosfeld E.; Steinberg H.; Paltiel Y.; Millo O. Magnetic-related States and Order Parameter Induced in a Conventional Superconductor by Nonmagnetic Chiral Molecules. Nano Lett. 2019, 19, 5167–5175. 10.1021/acs.nanolett.9b01552. 31361954

[ref120] Peer N.; Dujovne I.; Yochelis S.; Paltiel Y. Nanoscale Charge Separation Using Chiral Molecules. ACS Photonics 2015, 2, 1476–1481. 10.1021/acsphotonics.5b00343.

[ref121] Mathew S. P.; Mondal P. C.; Moshe H.; Mastai Y.; Naaman R. Non-magnetic Organic/Inorganic Spin Injector at Room Temperature. Appl. Phys. Lett. 2014, 105, 242408 10.1063/1.4904941.

[ref122] Torres-Cavanillas R.; Escorcia-Ariza G.; Brotons-Alcazar I.; Sanchis-Gual R.; Mondal P. C.; Rosaleny L. E.; Gimenez-Santamarina S.; Sessolo M.; Galbiati M.; Tatay S.; Gaita-Arino A.; Forment-Aliaga A.; Cardona-Serra S. Reinforced Room-Temperature Spin Filtering in Chiral Paramagnetic Metallopeptides. J. Am. Chem. Soc. 2020, 142, 17572–17580. 10.1021/jacs.0c07531. 32938174

[ref123] Kiran V.; Cohen S. R.; Naaman R. Structure Dependent Spin Selectivity in Electron Transport through Oligopeptides. J. Chem. Phys. 2017, 146, 092302 10.1063/1.4966237.

[ref124] Ozeri M.; Xu J.; Bauer G.; Olde Olthof L. A. B.; Kimbell G.; Wittmann A.; Yochelis S.; Fransson J.; Robinson J. W. A.; Paltiel Y.; Millo O. Modification of Weak Localization in Metallic Thin Films Due to the Adsorption of Chiral Molecules. J. Phys. Chem. Lett. 2023, 14, 4941–4948. 10.1021/acs.jpclett.3c00702. 37212799 PMC10240528

[ref125] Yang Q.; Zhang Z.; Jiang X.; Wang X.; Wang X.; Shang Z.; Liu F.; Deng J.; Zhai T.; Hong J.; Zhang Y.; Zhao W. Realization of High Spin Injection through Chiral Molecules and its Application in Logic Device. IEEE Electron Device Lett. 2022, 43, 1862–1865. 10.1109/LED.2022.3208856.

[ref126] Theiler P. M.; Ritz C.; Hofmann R.; Stemmer A. Detection of a Chirality-Induced Spin Selective Quantum Capacitance in α-Helical Peptides. Nano Lett. 2023, 23, 8280–8287. 10.1021/acs.nanolett.3c02483. 37650519 PMC10510583

[ref127] Naaman R.; Waldeck D. H. Comment on “Spin-Dependent Electron Transmission Model for Chiral Molecules in Mesoscopic Devices. Phys. Rev. B 2020, 101, 026403 10.1103/PhysRevB.101.026403.

[ref128] Kulkarni C.; Mondal A. K.; Das T. K.; Grinbom G.; Tassinari F.; Mabesoone M. F. J.; Meijer E. W.; Naaman R. Highly Efficient and Tunable Filtering of Electrons’ Spin by Supramolecular Chirality of Nanofiber-Based Materials. Adv. Mater. 2020, 32, e1904965 10.1002/adma.201904965. 31922628

[ref129] Mondal A. K.; Preuss M. D.; Sleczkowski M. L.; Das T. K.; Vantomme G.; Meijer E. W.; Naaman R. Spin Filtering in Supramolecular Polymers Assembled from Achiral Monomers Mediated by Chiral Solvents. J. Am. Chem. Soc. 2021, 143, 7189–7195. 10.1021/jacs.1c02983. 33926182 PMC8297732

[ref130] Kiran V.; Mathew S. P.; Cohen S. R.; Hernandez Delgado I.; Lacour J.; Naaman R. Helicenes-A New Class of Organic Spin Filter. Adv. Mater. 2016, 28, 1957–62. 10.1002/adma.201504725. 26742997

[ref131] Giaconi N.; Poggini L.; Lupi M.; Briganti M.; Kumar A.; Das T. K.; Sorrentino A. L.; Viglianisi C.; Menichetti S.; Naaman R.; Sessoli R.; Mannini M. Efficient Spin-Selective Electron Transport at Low Voltages of Thia-Bridged Triarylamine Hetero[4]helicenes Chemisorbed Monolayer. ACS Nano 2023, 17, 15189–15198. 10.1021/acsnano.3c04878. 37493644 PMC10416567

[ref132] Rodriguez R.; Naranjo C.; Kumar A.; Matozzo P.; Das T. K.; Zhu Q.; Vanthuyne N.; Gomez R.; Naaman R.; Sanchez L.; Crassous J. Mutual Monomer Orientation To Bias the Supramolecular Polymerization of [6]Helicenes and the Resulting Circularly Polarized Light and Spin Filtering Properties. J. Am. Chem. Soc. 2022, 144, 7709–7719. 10.1021/jacs.2c00556. 35404592 PMC9073930

[ref133] Liang Y.; Banjac K.; Martin K.; Zigon N.; Lee S.; Vanthuyne N.; Garces-Pineda F. A.; Galan-Mascaros J. R.; Hu X.; Avarvari N.; Lingenfelder M. Enhancement of Electrocatalytic Oxygen Evolution by Chiral Molecular Functionalization of Hybrid 2D Electrodes. Nat. Commun. 2022, 13, 3356 10.1038/s41467-022-31096-8. 35688831 PMC9187664

[ref134] Rodriguez R.; Naranjo C.; Kumar A.; Dhbaibi K.; Matozzo P.; Camerel F.; Vanthuyne N.; Gomez R.; Naaman R.; Sanchez L.; Crassous J. Weakly Self-Assembled [6] Helicenes: Circularly Polarized Light and Spin Filtering Properties. Chem. Eur. J. 2023, 29, e202302254 10.1002/chem.202302254. 37635073

[ref135] Niman C. M.; Sukenik N.; Dang T.; Nwachukwu J.; Thirumurthy M. A.; Jones A. K.; Naaman R.; Santra K.; Das T. K.; Paltiel Y.; Baczewski L. T.; El-Naggar M. Y. Bacterial Extracellular Electron Transfer Components are Spin Selective. J. Chem. Phys. 2023, 159, 145101 10.1063/5.0154211. 37811828

[ref136] Mishra S.; Pirbadian S.; Mondal A. K.; El-Naggar M. Y.; Naaman R. Spin-dependent Electron Transport through Bacterial Cell Surface Multiheme Electron Conduits. J. Am. Chem. Soc. 2019, 141, 19198–19202. 10.1021/jacs.9b09262. 31702906

[ref137] Varade V.; Markus T.; Vankayala K.; Friedman N.; Sheves M.; Waldeck D. H.; Naaman R. Bacteriorhodopsin Based Non-magnetic Spin Filters for Biomolecular Spintronics. Phys. Chem. Chem. Phys. 2018, 20, 1091–1097. 10.1039/C7CP06771B. 29238765

[ref138] Mondal P. C.; Fontanesi C.; Waldeck D. H.; Naaman R. Field and Chirality Effects on Electrochemical Charge Transfer Rates: Spin Dependent Electrochemistry. ACS Nano 2015, 9, 3377–3384. 10.1021/acsnano.5b00832. 25752750

[ref139] Carmeli I.; Senthil Kumar K.; Heifler O.; Carmeli C.; Naaman R. Spin Selectivity in Electron Transfer in Photosystem I. Angew. Chem., Int. Ed. Engl. 2014, 53, 8953–8958. 10.1002/anie.201404382. 24989350

[ref140] Gupta R.; Chinnasamy H. V.; Sahu D.; Matheshwaran S.; Sow C.; Chandra Mondal P. Spin-dependent Electrified Protein Interfaces for Probing the CISS Effect. J. Chem. Phys. 2023, 159, 024708 10.1063/5.0156479. 37439472

[ref141] Mishra D.; Markus T. Z.; Naaman R.; Kettner M.; Göhler B.; Zacharias H.; Friedman N.; Sheves M.; Fontanesi C. Spin-dependent Electron Transmission through Bacteriorhodopsin Embedded in Purple Membrane. Proc. Natl. Acad. Sci. U.S.A. 2013, 110, 14872–14876. 10.1073/pnas.1311493110. 23980184 PMC3773807

[ref142] Sang Y.; Mishra S.; Tassinari F.; Karuppannan S. K.; Carmieli R.; Teo R. D.; Migliore A.; Beratan D. N.; Gray H. B.; Pecht I.; Fransson J.; Waldeck D. H.; Naaman R. Temperature Dependence of Charge and Spin Transfer in Azurin. J. Phys. Chem. C 2021, 125, 9875–9883. 10.1021/acs.jpcc.1c01218. PMC8154855 34055128

[ref143] Ghosh S.; Banerjee-Ghosh K.; Levy D.; Riven I.; Naaman R.; Haran G. Substrates Modulate Charge-Reorganization Allosteric Effects in Protein-Protein Association. J. Phys. Chem. Lett. 2021, 12, 2805–2808. 10.1021/acs.jpclett.1c00437. 33710900 PMC8041378

[ref144] Jia L.; Wang C.; Zhang Y.; Yang L.; Yan Y. Efficient Spin Selectivity in Self-Assembled Superhelical Conducting Polymer Microfibers. ACS Nano 2020, 14, 6607–6615. 10.1021/acsnano.9b07681. 32422046

[ref145] Tassinari F.; Banerjee-Ghosh K.; Parenti F.; Kiran V.; Mucci A.; Naaman R. Enhanced Hydrogen Production With Chiral Conductive Polymer-Based Electrodes. J. Phys. Chem. C 2017, 121, 15777–15783. 10.1021/acs.jpcc.7b04194. 28650163

[ref146] Mondal P. C.; Kantor-Uriel N.; Mathew S. P.; Tassinari F.; Fontanesi C.; Naaman R. Chiral Conductive Polymers as Spin Filters. Adv. Mater. 2015, 27, 1924–1927. 10.1002/adma.201405249. 25619708

[ref147] Mishra S.; Mondal A. K.; Smolinsky E. Z. B.; Naaman R.; Maeda K.; Nishimura T.; Taniguchi T.; Yoshida T.; Takayama K.; Yashima E. Spin Filtering Along Chiral Polymers. Angew. Chem., Int. Ed. Engl. 2020, 59, 14671–14676. 10.1002/anie.202006570. 32533565 PMC7496609

[ref148] Hong K.-I.; Kumar A.; Garcia A. M.; Majumder S.; Carretero A. Electron Spin Polarization in Supramolecular Polymers with Complex Pathways. ChemRxiv. 2023, 10.26434/chemrxiv-2023-j1qt8. 37712794

[ref149] Ha Nguyen T. N.; Paltiel Y.; Baczewski L. T.; Tegenkamp C. Spin Polarization of Polyalanine Molecules in 2D and Dimer-Row Assemblies Adsorbed on Magnetic Substrates: The Role of Coupling, Chirality, and Coordination. ACS Appl. Mater. Interfaces 2023, 15, 17406–17412. 10.1021/acsami.3c01429. 36952617

[ref150] Bhowmick D. K.; Das T. K.; Santra K.; Mondal A. K.; Tassinari F.; Schwarz R.; Diesendruck C. E.; Naaman R. Spin-induced Asymmetry Reaction-The Formation of Asymmetric Carbon by Electropolymerization. Sci. Adv. 2022, 8, eabq2727 10.1126/sciadv.abq2727. 35947656 PMC9365291

[ref151] Ma W.; Xu L.; de Moura A. F.; Wu X.; Kuang H.; Xu C.; Kotov N. A. Chiral Inorganic Nanostructures. Chem. Rev. 2017, 117, 8041–8093. 10.1021/acs.chemrev.6b00755. 28426196

[ref152] Fan J.; Kotov N. A. Chiral Nanoceramics. Adv. Mater. 2020, 32, e1906738 10.1002/adma.201906738. 32500963

[ref153] Long G. K.; Sabatini R.; Saidaminov M. I.; Lakhwani G.; Rasmita A.; Liu X. G.; Sargent E. H.; Gao W. B. Chiral-perovskite Optoelectronics. Nat. Rev. Mater. 2020, 5, 423–439. 10.1038/s41578-020-0181-5.

[ref154] Mondal P. C.; Asthana D.; Parashar R. K.; Jadhav S. Imprinting Chirality in Inorganic Nanomaterials for Optoelectronic and Bio-applications: Strategies, Challenges, and Opportunities. Mater. Adv. 2021, 2, 7620–7637. 10.1039/D1MA00846C.

[ref155] Ko C. H.; Zhu Q. R.; Tassinari F.; Bullard G.; Zhang P.; Beratan D. N.; Naaman R.; Therien M. J. Twisted Molecular Wires Polarize Spin Currents at Room Temperature. Proc. Natl. Acad. Sci. U.S.A. 2022, 119, e2116180119 10.1073/pnas.2116180119. 35115404 PMC8833206

[ref156] Cardona-Serra S.; Rosaleny L. E.; Gimenez-Santamarina S.; Martinez-Gil L.; Gaita-Arino A. Towards Peptide-based Tunable Multistate Memristive Materials. Phys. Chem. Chem. Phys. 2021, 23, 1802–1810. 10.1039/D0CP05236A. 33434247

[ref157] Sang Y.; Zhu Q.; Zhou X.; Jiang Y.; Zhang L.; Liu M. Ultrasound-Directed Symmetry Breaking and Spin Filtering of Supramolecular Assemblies from only Achiral Building Blocks. Angew. Chem., Int. Ed. Engl. 2023, 62, e202215867 10.1002/anie.202215867. 36522559

[ref158] Mtangi W.; Tassinari F.; Vankayala K.; Vargas Jentzsch A.; Adelizzi B.; Palmans A. R.; Fontanesi C.; Meijer E. W.; Naaman R. Control of Electrons’ Spin Eliminates Hydrogen Peroxide Formation During Water Splitting. J. Am. Chem. Soc. 2017, 139, 2794–2798. 10.1021/jacs.6b12971. 28132505 PMC5330654

[ref159] Wang C.; Guo A. M.; Sun Q. F.; Yan Y. Efficient Spin-Dependent Charge Transmission and Improved Enantioselective Discrimination Capability in Self-Assembled Chiral Coordinated Monolayers. J. Phys. Chem. Lett. 2021, 12, 10262–10269. 10.1021/acs.jpclett.1c03106. 34652163

[ref160] Miwa S.; Kondou K.; Sakamoto S.; Nihonyanagi A.; Araoka F.; Otani Y.; Miyajima D. Chirality-induced Effective Magnetic Field in a Phthalocyanine Molecule. Appl. Phys. Express. 2020, 13, 113001 10.35848/1882-0786/abbf67.

[ref161] Kondou K.; Shiga M.; Sakamoto S.; Inuzuka H.; Nihonyanagi A.; Araoka F.; Kobayashi M.; Miwa S.; Miyajima D.; Otani Y. Chirality-Induced Magnetoresistance Due to Thermally Driven Spin Polarization. J. Am. Chem. Soc. 2022, 144, 7302–7307. 10.1021/jacs.2c00496. 35414173 PMC9052755

[ref162] Aizawa H.; Sato T.; Maki-Yonekura S.; Yonekura K.; Takaba K.; Hamaguchi T.; Minato T.; Yamamoto H. M. Enantioselectivity of Discretized Helical Supramolecule Consisting of Achiral Cobalt Phthalocyanines via Chiral-induced Spin Selectivity Effect. Nat. Commun. 2023, 14, 4530 10.1038/s41467-023-40133-z. 37507380 PMC10382588

[ref163] Bloom B. P.; Kiran V.; Varade V.; Naaman R.; Waldeck D. H. Spin Selective Charge Transport through Cysteine Capped CdSe Quantum Dots. Nano Lett. 2016, 16, 4583–9. 10.1021/acs.nanolett.6b01880. 27336320

[ref164] Fridman H. T.; Dehnel J.; Yochelis S.; Lifshitz E.; Paltiel Y. Spin-Exciton Delocalization Enhancement in Multilayer Chiral Linker/Quantum Dot Structures. J. Phys. Chem. Lett. 2019, 10, 3858–3862. 10.1021/acs.jpclett.9b01433. 31241942

[ref165] Bezen L.; Yochelis S.; Jayarathna D.; Bhunia D.; Achim C.; Paltiel Y. Chiral Molecule-Enhanced Extinction Ratios of Quantum Dots Coupled to Random Plasmonic Structures. Langmuir 2018, 34, 3076–3081. 10.1021/acs.langmuir.8b00155. 29424540

[ref166] Cohen E.; Komm P.; Rosenthal-Strauss N.; Dehnel J.; Lifshitz E.; Yochelis S.; Levine R. D.; Remacle F.; Fresch B.; Marcus G.; Paltiel Y. Fast Energy Transfer in CdSe Quantum Dot Layered Structures: Controlling Coupling with Covalent-Bond Organic Linkers. J. Phys. Chem. C 2018, 122, 5753–5758. 10.1021/acs.jpcc.7b11799.

[ref167] Al-Bustami H.; Bloom B. P.; Ziv A.; Goldring S.; Yochelis S.; Naaman R.; Waldeck D. H.; Paltiel Y. Optical Multilevel Spin Bit Device Using Chiral Quantum Dots. Nano Lett. 2020, 20, 8675–8681. 10.1021/acs.nanolett.0c03445. 33185449

[ref168] Lu H.; Wang J.; Xiao C.; Pan X.; Chen X.; Brunecky R.; Berry J. J.; Zhu K.; Beard M. C.; Vardeny Z. V. Spin-dependent Charge Transport through 2D Chiral Hybrid Lead-iodide Perovskites. Sci. Adv. 2019, 5, eaay0571 10.1126/sciadv.aay0571. 31840072 PMC6897542

[ref169] Lee C. U.; Ma S.; Ahn J.; Kyhm J.; Tan J.; Lee H.; Jang G.; Park Y. S.; Yun J.; Lee J.; Son J.; Park J. S.; Moon J. Tailoring the Time-Averaged Structure for Polarization-Sensitive Chiral Perovskites. J. Am. Chem. Soc. 2022, 144, 16020–16033. 10.1021/jacs.2c05849. 36036662

[ref170] Wang Q.; Lu Y.; He R. L.; Chen R.; Qiao L.; Pan F.; Yang Z.; Song C. Spin Selectivity in Chiral Hybrid Cobalt Halide Films with Ultrasmooth Surface. Small Methods 2022, 6, e2201048 10.1002/smtd.202201048. 36403249

[ref171] Kim Y. H.; Zhai Y.; Lu H.; Pan X.; Xiao C.; Gaulding E. A.; Harvey S. P.; Berry J. J.; Vardeny Z. V.; Luther J. M.; Beard M. C. Chiral-induced Spin Selectivity Enables a Room-temperature Spin Light-emitting Diode. Science 2021, 371, 1129–1133. 10.1126/science.abf5291. 33707260

[ref172] Lu Y.; Wang Q.; Chen R. Y.; Qiao L. L.; Zhou F. X.; Yang X.; Wang D.; Cao H.; He W. L.; Pan F.; Yang Z.; Song C. Spin-Dependent Charge Transport in 1D Chiral Hybrid Lead-Bromide Perovskite with High Stability. Adv. Funct. Mater. 2021, 31, 2104605 10.1002/adfm.202104605.

[ref173] Lu H.; Xiao C.; Song R.; Li T.; Maughan A. E.; Levin A.; Brunecky R.; Berry J. J.; Mitzi D. B.; Blum V.; Beard M. C. Highly Distorted Chiral Two-Dimensional Tin Iodide Perovskites for Spin Polarized Charge Transport. J. Am. Chem. Soc. 2020, 142, 13030–13040. 10.1021/jacs.0c03899. 32602710

[ref174] Maiti A.; Pal A. J. Spin-Selective Charge Transport in Lead-Free Chiral Perovskites: The Key towards High-Anisotropy in Circularly-Polarized Light Detection. Angew. Chem., Int. Ed. Engl. 2022, 61, e202214161 10.1002/anie.202214161. 36325645

[ref175] Lu Y.; Wang Q.; He R.; Zhou F.; Yang X.; Wang D.; Cao H.; He W.; Pan F.; Yang Z.; Song C. Highly Efficient Spin-Filtering Transport in Chiral Hybrid Copper Halides. Angew. Chem., Int. Ed. Engl. 2021, 60, 23578–23583. 10.1002/anie.202109595. 34423529

[ref176] Chen Y.; Liu Z.; Li J.; Cheng X.; Ma J.; Wang H.; Li D. Robust Interlayer Coupling in Two-Dimensional Perovskite/Monolayer Transition Metal Dichalcogenide Heterostructures. ACS Nano 2020, 14, 10258–10264. 10.1021/acsnano.0c03624. 32806069

[ref177] Chen Y.; Ma J.; Liu Z.; Li J.; Duan X.; Li D. Manipulation of Valley Pseudospin by Selective Spin Injection in Chiral Two-Dimensional Perovskite/Monolayer Transition Metal Dichalcogenide Heterostructures. ACS Nano 2020, 14, 15154–15160. 10.1021/acsnano.0c05343. 33108721

[ref178] Shrestha S.; Li M.; Park S.; Tong X.; DiMarzio D.; Cotlet M. Room Temperature Valley Polarization via Spin Selective Charge Transfer. Nat. Commun. 2023, 14, 1–9. 10.1038/s41467-023-40967-7. 37633986 PMC10460417

[ref179] Wang J.; Lu H.; Pan X.; Xu J.; Liu H.; Liu X.; Khanal D. R.; Toney M. F.; Beard M. C.; Vardeny Z. V. Spin-Dependent Photovoltaic and Photogalvanic Responses of Optoelectronic Devices Based on Chiral Two-Dimensional Hybrid Organic-Inorganic Perovskites. ACS Nano 2021, 15 (1), 588–595. 10.1021/acsnano.0c05980. 33241679

[ref180] Kim Y. H.; Song R. Y.; Hao J.; Zhai Y. X.; Yan L.; Moot T.; Palmstrom A. F.; Brunecky R.; You W.; Berry J. J.; Blackburn J. L.; Beard M. C.; Blum V.; Luther J. M. The Structural Origin of Chiroptical Properties in Perovskite Nanocrystals with Chiral Organic Ligands. Adv. Funct. Mater. 2022, 32, 2200454 10.1002/adfm.202200454.

[ref181] Hao J.; Lu H.; Mao L.; Chen X.; Beard M. C.; Blackburn J. L. Direct Detection of Circularly Polarized Light Using Chiral Copper Chloride-Carbon Nanotube Heterostructures. ACS Nano 2021, 15 (4), 7608–7617. 10.1021/acsnano.1c01134. 33821628 PMC10156083

[ref182] Qian Q.; Ren H.; Zhou J.; Wan Z.; Zhou J.; Yan X.; Cai J.; Wang P.; Li B.; Sofer Z.; Li B.; Duan X.; Pan X.; Huang Y.; Duan X. Chiral Molecular Intercalation Superlattices. Nature 2022, 606, 902–908. 10.1038/s41586-022-04846-3. 35768590

[ref183] Bai X.; Cao Y.; Xu Y.; Huang W.; Deng P.; Tian X.; Liu Z.; Wang J.; Tu J. Enhanced Photocatalytic Hydrolysis Performance of Chiral Molecule Loaded Titanium Disulfide Nanosheets. ChemPhysChem 2022, 23, e202200156 10.1002/cphc.202200156. 35393729

[ref184] Bian Z.; Kato K.; Ogoshi T.; Cui Z.; Sa B.; Tsutsui Y.; Seki S.; Suda M. Hybrid Chiral MoS_2_ Layers for Spin-Polarized Charge Transport and Spin-Dependent Electrocatalytic Applications. Adv. Sci. 2022, 9, e2201063 10.1002/advs.202201063. PMC9189682 35481673

[ref185] Bian Z.; Nakano Y.; Miyata K.; Oya I.; Nobuoka M.; Tsutsui Y.; Seki S.; Suda M. Chiral van der Waals Superlattices for Enhanced Spin-Selective Transport and Spin-Dependent Electrocatalytic Performance. Adv. Mater. 2023, 35, 2306061 10.1002/adma.202306061. 37695880

[ref186] Gao G.; Zhu J.; Wei S.; Cao Y.; Huang W.; Liu Z.; Wang J.; Shen Y. Chiral Molecule Induced Valley Polarization Enhancement of MoS_2_. Phys. Chem. Chem. Phys. 2023, 25, 18998–19003. 10.1039/D2CP04397A. 37416951

[ref187] Aivazian G.; Gong Z. R.; Jones A. M.; Chu R. L.; Yan J.; Mandrus D. G.; Zhang C. W.; Cobden D.; Yao W.; Xu X. Magnetic Control of Valley Pseudospin in Monolayer WSe_2_. Nat. Phys. 2015, 11, 148–152. 10.1038/nphys3201.

[ref188] Srivastava A.; Sidler M.; Allain A. V.; Lembke D. S.; Kis A.; Imamoglu A. Valley Zeeman Effect in Elementary Optical Excitations of Monolayer WSe_2_. Nat. Phys. 2015, 11, 141–147. 10.1038/nphys3203.

[ref189] Liu X.; Du Y.; Mourdikoudis S.; Zheng G.; Wong K. Y. Chiral Magnetic Oxide Nanomaterials: Magnetism Meets Chirality. Adv. Optic. Mater. 2023, 11, 2202859 10.1002/adom.202202859.

[ref190] Kothari H. M.; Kulp E. A.; Boonsalee S.; Nikiforov M. P.; Bohannan E. W.; Poizot P.; Nakanishi S.; Switzer J. A. Enantiospecific Electrodeposition of Chiral CuO Films from Copper(II) Complexes of Tartaric and Amino Acids on Single-Crystal Au(001). Chem. Mater. 2004, 16, 4232–4244. 10.1021/cm048939x.

[ref191] Switzer J. A.; Kothari H. M.; Poizot P.; Nakanishi S.; Bohannan E. W. Enantiospecific Electrodeposition of a Chiral Catalyst. Nature 2003, 425, 490–3. 10.1038/nature01990. 14523441

[ref192] Ghosh S. Chiral Induced Spin Selectivity Effect: Fundamental Studies and Applications. Ph.D., University of Pittsburgh, Ann Arbor, 2021.

[ref193] Im H.; Ma S.; Lee H.; Park J.; Park Y. S.; Yun J.; Lee J.; Moon S.; Moon J. Elucidating the Chirality Transfer Mechanisms During Enantioselective Synthesis for the Spin-controlled Oxygen Evolution Reaction. Energy Environ. Sci. 2023, 16, 1797–1797. 10.1039/D3EE90020G.

[ref194] Bai T.; Ai J.; Liao L.; Luo J.; Song C.; Duan Y.; Han L.; Che S. Chiral Mesostructured NiO Films with Spin Polarisation. Angew. Chem., Int. Ed. Engl. 2021, 60, 9421–9426. 10.1002/anie.202101069. 33554464

[ref195] Al-Bustami H.; Khaldi S.; Shoseyov O.; Yochelis S.; Killi K.; Berg I.; Gross E.; Paltiel Y.; Yerushalmi R. Atomic and Molecular Layer Deposition of Chiral Thin Films Showing up to 99% Spin Selective Transport. Nano Lett. 2022, 22, 5022–5028. 10.1021/acs.nanolett.2c01953. 35679580

[ref196] Wang H. L.; Du C. H.; Pu Y.; Adur R.; Hammel P. C.; Yang F. Y. Scaling of Spin Hall Angle in 3d, 4d, and 5d Metals from Y_3_Fe_5_O_12_/Metal Spin Pumping. Phys. Rev. Lett. 2014, 112, 197201 10.1103/PhysRevLett.112.197201. 24877962

[ref197] Nabei Y.; Hirobe D.; Shimamoto Y.; Shiota K.; Inui A.; Kousaka Y.; Togawa Y.; Yamamoto H. M. Current-induced Bulk Magnetization of a Chiral Crystal CrNb_3_S_6_. Appl. Phys. Lett. 2020, 117, 052408 10.1063/5.0017882.

[ref198] Shishido H.; Sakai R.; Hosaka Y.; Togawa Y. Detection of chirality-induced Spin Polarization over Millimeters in Polycrystalline Bulk Samples of Chiral Disilicides NbSi_2_ and TaSi_2_. Appl. Phys. Lett. 2021, 119, 182403 10.1063/5.0074293.

[ref199] Shiota K.; Inui A.; Hosaka Y.; Amano R.; Onuki Y.; Hedo M.; Nakama T.; Hirobe D.; Ohe J. I.; Kishine J. I.; Yamamoto H. M.; Shishido H.; Togawa Y. Chirality-Induced Spin Polarization over Macroscopic Distances in Chiral Disilicide Crystals. Phys. Rev. Lett. 2021, 127, 126602 10.1103/PhysRevLett.127.126602. 34597079

[ref200] Calavalle F.; Suarez-Rodriguez M.; Martin-Garcia B.; Johansson A.; Vaz D. C.; Yang H.; Maznichenko I. V.; Ostanin S.; Mateo-Alonso A.; Chuvilin A.; Mertig I.; Gobbi M.; Casanova F.; Hueso L. E. Gate-tuneable and Chirality-dependent Charge-to-spin Conversion in Tellurium Nanowires. Nat. Mater. 2022, 21, 526–532. 10.1038/s41563-022-01211-7. 35256792

[ref201] Shishido H.; Hosaka Y.; Monden K.; Inui A.; Sayo T.; Kousaka Y.; Togawa Y. Spin Polarization Gate Device Based on the Chirality-induced Spin Selectivity and Robust Nonlocal Spin Polarization. J. Chem. Phys. 2023, 159, 064502 10.1063/5.0156505. 37551815

[ref202] Hoff D. A.; Rego L. G. C. Chirality-Induced Propagation Velocity Asymmetry. Nano Lett. 2021, 21, 8190–8196. 10.1021/acs.nanolett.1c02636. 34551246

[ref203] Kousaka Y.; Sayo T.; Iwasaki S.; Saki R.; Shimada C.; Shishido H.; Togawa Y. Chirality-selected Srystal Growth and Spin Polarization Over Centimeters of Transition Metal Disilicide Crystals. Jpn. J. Appl. Phys. 2022, 62, 015506 10.35848/1347-4065/aca8e2.

[ref204] Huizi-Rayo U.; Gutierrez J.; Seco J. M.; Mujica V.; Diez-Perez I.; Ugalde J. M.; Tercjak A.; Cepeda J.; San Sebastian E. An Ideal Spin Filter: Long-Range, High-Spin Selectivity in Chiral Helicoidal 3-Dimensional Metal Organic Frameworks. Nano Lett. 2020, 20, 8476–8482. 10.1021/acs.nanolett.0c02349. 33170013

[ref205] Mondal A. K.; Brown N.; Mishra S.; Makam P.; Wing D.; Gilead S.; Wiesenfeld Y.; Leitus G.; Shimon L. J. W.; Carmieli R.; Ehre D.; Kamieniarz G.; Fransson J.; Hod O.; Kronik L.; Gazit E.; Naaman R. Long-Range Spin-Selective Transport in Chiral Metal-Organic Crystals with Temperature-Activated Magnetization. ACS Nano 2020, 14, 16624–16633. 10.1021/acsnano.0c07569. 33095016 PMC7760088

[ref206] Kumar Das T.; Mondal A. K.; Tiwari O. S.; Makam P.; Leitus G.; Gazit E.; Claudio F.; Naaman R. Spin-induced Electron Transmission through Metal-organic Chiral Crystals. Phys. Chem. Chem. Phys. 2023, 25, 22124–22129. 10.1039/D3CP02579A. 37563955

[ref207] Amsallem D.; Kumar A.; Naaman R.; Gidron O. Spin Polarization through Axially Chiral Linkers: Length Dependence and Correlation with the Dissymmetry Factor. Chirality 2023, 35, 562–568. 10.1002/chir.23556. 36896481

[ref208] Aragonès A. C.; Aravena D.; Ugalde J. M.; Medina E.; Gutierrez R.; Ruiz E.; Mujica V.; Díez-Pérez I. Magnetoresistive Single-Molecule Junctions: the Role of the Spinterface and the CISS Effect. Isr. J. Chem. 2022, 62, e202200090 10.1002/ijch.202200090.

[ref209] Yang C.; Li Y. W.; Zhou S. Y.; Guo Y. L.; Jia C. C.; Liu Z. R.; Houk K. N.; Dubi Y.; Guo X. F. Real-time Monitoring of Reaction Stereochemistry through Single-molecule Observations of Chirality-induced Spin Selectivity. Nat. Chem. 2023, 15, 972–979. 10.1038/s41557-023-01212-2. 37188972

[ref210] Varela S.; Mujica V.; Medina E. Effective Spin-orbit Couplings in an Analytical Tight-binding Model of DNA: Spin Filtering and Chiral Spin Transport. Phys. Rev. B 2016, 93, 155436 10.1103/PhysRevB.93.155436.

[ref211] Guo A. M.; Sun Q. F. Spin-selective Transport of Electrons in DNA Double Helix. Phys. Rev. Lett. 2012, 108, 218102 10.1103/PhysRevLett.108.218102. 23003304

[ref212] Medina E.; Gonzalez-Arraga L. A.; Finkelstein-Shapiro D.; Berche B.; Mujica V. Continuum Model for Chiral Induced Spin Selectivity in Helical Molecules. J. Chem. Phys. 2015, 142, 194308 10.1063/1.4921310. 26001462

[ref213] Gutierrez R.; Díaz E.; Naaman R.; Cuniberti G. Spin-selective Transport through Helical Molecular Systems. Phys. Rev. B 2012, 85, 081404 10.1103/PhysRevB.85.081404.

[ref214] Geyer M.; Gutierrez R.; Mujica V.; Cuniberti G. Chirality-Induced Spin Selectivity in a Coarse-Grained Tight-Binding Model for Helicene. J. Phys. Chem. C 2019, 123, 27230–27241. 10.1021/acs.jpcc.9b07764.

[ref215] Mao S. N.; Amin N.; Murdock E. Temperature Dependence of Giant Magnetoresistance Properties of NiMn Pinned Spin Valves. J. Appl. Phys. 1998, 83, 6807–6809. 10.1063/1.367639.

[ref216] Ju H. L.; Gopalakrishnan J.; Peng J. L.; Li Q.; Xiong G. C.; Venkatesan T.; Greene R. L. Dependence of Giant Magnetoresistance on Oxygen Stoichiometry and Magnetization in Polycrystalline La_0.67_Ba_0.33_MnO_z_. Phys. Rev. B 1995, 51, 6143–6146. 10.1103/PhysRevB.51.6143. 9979541

[ref217] Rahman M. W.; Manas-Torres M. C.; Firouzeh S.; Illescas-Lopez S.; Cuerva J. M.; Lopez-Lopez M. T.; de Cienfuegos L. A.; Pramanik S. Chirality-Induced Spin Selectivity in Heterochiral Short-Peptide-Carbon-Nanotube Hybrid Networks: Role of Supramolecular Chirality. ACS Nano 2022, 16, 16941–16953. 10.1021/acsnano.2c07040. 36219724

[ref218] Rahman M. W.; Manas-Torres M. C.; Firouzeh S.; Cuerva J. M.; Alvarez de Cienfuegos L.; Pramanik S. Molecular Functionalization and Emergence of Long-Range Spin-Dependent Phenomena in Two-Dimensional Carbon Nanotube Networks. ACS Nano 2021, 15, 20056–20066. 10.1021/acsnano.1c07739. 34870421

[ref219] Fransson J. Chirality-induced Spin Selectivity: The Role of Electron Correlations. J. Phys. Chem. Lett. 2019, 10, 7126–7132. 10.1021/acs.jpclett.9b02929. 31657931

[ref220] Fransson J. Charge Redistribution and Spin Polarization Driven by Correlation Induced Electron Exchange in Chiral Molecules. Nano Lett. 2021, 21, 3026–3032. 10.1021/acs.nanolett.1c00183. 33759530 PMC8050826

[ref221] Lu Y.; Qiu T.; Bloom B. P.; Subotnik J. E.; Waldeck D. H. Spin-Based Chiral Separations and the Importance of Molecule-Solvent Interactions. J. Phys. Chem. C 2023, 127, 14155–14162. 10.1021/acs.jpcc.3c01159. PMC10389781 37529661

[ref222] Lu Y.; Joy M.; Bloom B. P.; Waldeck D. H. Beyond Stereoisomeric Effects: Exploring the Importance of Intermolecular Electron Spin Interactions in Biorecognition. J. Phys. Chem. Lett. 2023, 14, 7032–7037. 10.1021/acs.jpclett.3c01595. 37524051 PMC10424231

[ref223] Al-Bustami H.; Koplovitz G.; Primc D.; Yochelis S.; Capua E.; Porath D.; Naaman R.; Paltiel Y. Single Nanoparticle Magnetic Spin Memristor. Small 2018, 14 (30), 1801249 10.1002/smll.201801249. 29952065

[ref224] Wei M.; Lu X.; Qiao J.; Ren S.; Hao X. T.; Qin W. Response of Spin to Chiral Orbit and Phonon in Organic Chiral Ferrimagnetic Crystals. ACS Nano 2022, 16, 13049–13056. 10.1021/acsnano.2c05601. 35943139

[ref225] Zhou Y.; Bai T.; Duan Y. Chiral Mesostructured NiFe_2_O_4_ Films with Chirality Induced Spin Selectivity. Chem. Commun. 2023, 59, 13207–13210. 10.1039/D3CC03183G. 37853755

[ref226] Alpern H.; Katzir E.; Yochelis S.; Katz N.; Paltiel Y.; Millo O. Unconventional Superconductivity Induced in Nb Films by Adsorbed Chiral Molecules. New J. Phys. 2016, 18, 113048 10.1088/1367-2630/18/11/113048.

[ref227] Periyasamy M.; Bradshaw H.; Sukenik N.; Alpern H.; Yochelis S.; Robinson J. W. A.; Millo O.; Paltiel Y. Universal Proximity Effects in Hybrid Superconductor-linker Molecule-nanoparticle Systems: The Effect of Molecular Chirality. Appl. Phys. Lett. 2020, 117, 242601 10.1063/5.0030892.

[ref228] Sukenik N.; Alpern H.; Katzir E.; Yochelis S.; Millo O.; Paltiel Y. Proximity Effect through Chiral Molecules in Nb-Graphene-Based Devices. Adv. Mater. Technol. 2018, 3, 1700300 10.1002/admt.201700300.

[ref229] Shapira T.; Alpern H.; Yochelis S.; Lee T. K.; Kaun C. C.; Paltiel Y.; Koren G.; Millo O. Unconventional Order Parameter Induced by Helical Chiral Molecules Adsorbed on a Metal Proximity Coupled to a Superconductor. Phys. Rev. B 2018, 98, 214513 10.1103/PhysRevB.98.214513.

[ref230] Dianat A.; Gutierrez R.; Alpern H.; Mujica V.; Ziv A.; Yochelis S.; Millo O.; Paltiel Y.; Cuniberti G. Role of Exchange Interactions in the Magnetic Response and Intermolecular Recognition of Chiral Molecules. Nano Lett. 2020, 20, 7077–7086. 10.1021/acs.nanolett.0c02216. 32786950

[ref231] Al-Bustami H.; Belsey S.; Metzger T.; Voignac D.; Yochelis S.; Shoseyov O.; Paltiel Y. Spin-Induced Organization of Cellulose Nanocrystals. Biomacromolecules 2022, 23, 2098–2105. 10.1021/acs.biomac.2c00099. 35289591

[ref232] Levy H. M.; Schneider A.; Tiwari S.; Zer H.; Yochelis S.; Goloubinoff P.; Keren N.; Paltiel Y. The Effect of Spin Exchange Interaction on Protein Structural Stability. Phys. Chem. Chem. Phys. 2022, 24, 29176–29185. 10.1039/D2CP03331C. 36444947

[ref233] Barron L. D. Symmetry and Chirality: Where Physics Shakes Hands with Chemistry and Biology. Isr. J. Chem. 2021, 61, 517–529. 10.1002/ijch.202100044.

[ref234] Kishine J. i.; Kusunose H.; Yamamoto H. M. On the Definition of Chirality and Enantioselective Fields. Isr. J. Chem. 2022, 62, e202200049 10.1002/ijch.202200049.

[ref235] Huisman K. H.; Heinisch J.-B. M.-Y.; Thijssen J. M. Chirality-Induced Spin Selectivity (CISS) Effect: Magnetocurrent-Voltage Characteristics with Coulomb Interactions I. J. Phys. Chem. C 2023, 127, 6900–6905. 10.1021/acs.jpcc.2c08807. PMC10108364 37081995

[ref236] Yeganeh S.; Ratner M. A.; Medina E.; Mujica V. Chiral Electron Transport: Scattering through Helical Potentials. J. Chem. Phys. 2009, 131, 014707 10.1063/1.3167404. 19586117

[ref237] Shitade A.; Minamitani E. Geometric Spin-orbit Coupling and Chirality-induced Spin Selectivity. New. J. Phys. 2020, 22, 113023 10.1088/1367-2630/abc920.

[ref238] Yu Z.-G. Chirality-induced Spin-orbit Coupling, Spin Transport, and Natural Optical Activity in Hybrid Organic-inorganic Perovskites. J. Phys. Chem. Lett. 2020, 11, 8638–8646. 10.1021/acs.jpclett.0c02589. 32991181

[ref239] Sahu P.; Bhowal S.; Satpathy S. Effect of the Inversion Symmetry Breaking on the Orbital Hall Effect: A Model Study. Phys. Rev. B 2021, 103, 085113 10.1103/PhysRevB.103.085113.

[ref240] Yang X.; van der Wal C. H.; van Wees B. J. Spin-dependent Electron Transmission Model for Chiral Molecules in Mesoscopic Devices. Phys. Rev. B 2019, 99, 024418 10.1103/PhysRevB.99.024418.

[ref241] Boulougouris G. C. Multidimensional Direct Free Energy Perturbation. J. Chem. Phys. 2013, 138, 114111 10.1063/1.4795319. 23534631

[ref242] Liu Y.; Xiao J.; Koo J.; Yan B. Chirality-driven Topological Electronic Structure of DNA-like Materials. Nat. Mater. 2021, 20, 638–644. 10.1038/s41563-021-00924-5. 33558719 PMC7610709

[ref243] Dubi Y. Spinterface Chirality-induced Spin Selectivity Effect in Bio-molecules. Chem. Sci. 2022, 13, 10878–10883. 10.1039/D2SC02565E. 36320704 PMC9491198

[ref244] Zöllner M. S.; Saghatchi A.; Mujica V.; Herrmann C. Influence of Electronic Structure Modeling and Junction Structure on First-principles Chiral Induced Spin Selectivity. J. Chem. Theory Comput. 2020, 16, 7357–7371. 10.1021/acs.jctc.0c00621. 33167619

[ref245] Gersten J.; Kaasbjerg K.; Nitzan A. Induced Spin Filtering in Electron Transmission through Chiral Molecular Layers Adsorbed on Metals with Strong Spin-orbit Coupling. J. Chem. Phys. 2013, 139, 114111 10.1063/1.4820907. 24070283

[ref246] Naskar S.; Mujica V.; Herrmann C. Chiral-Induced Spin Selectivity and Non-equilibrium Spin Accumulation in Molecules and Interfaces: A First-Principles Study. J. Phys. Chem. Lett. 2023, 14, 694–701. 10.1021/acs.jpclett.2c03747. 36638217

[ref247] Huisman K. H.; Thijssen J. M. CISS Effect: A Magnetoresistance Through Inelastic Scattering. J. Phys. Chem. C 2021, 125, 23364–23369. 10.1021/acs.jpcc.1c06193. PMC8558858 34737840

[ref248] Dednam W.; García-Blázquez M. A.; Zotti L. A.; Lombardi E. B.; Sabater C.; Pakdel S.; Palacios J. A Group-Theoretic Approach to the Origin of Chirality-Induced Spin-Selectivity in Nonmagnetic Molecular Junctions. ACS Nano 2023, 17, 6452–6465. 10.1021/acsnano.2c11410. 36947721 PMC10100547

[ref249] Vittmann C.; Kessing R. K.; Lim J.; Huelga S. F.; Plenio M. B. Interface-induced Conservation of Momentum Seads to Chiral-induced Spin Selectivity. J. Phys. Chem. Lett. 2022, 13, 1791–1796. 10.1021/acs.jpclett.1c03975. 35170964

[ref250] Adhikari Y.; Liu T.; Wang H.; Hua Z.; Liu H.; Lochner E.; Schlottmann P.; Yan B.; Zhao J.; Xiong P. Interplay of Structural Chirality, Electron Spin and Topological Orbital in Chiral Molecular Spin Valves. Nat. Commun. 2023, 14, 5163 10.1038/s41467-023-40884-9. 37620378 PMC10449876

[ref251] Naskar S.; Saghatchi A.; Mujica V.; Herrmann C. Common Trends of Chiral Induced Spin Selectivity and Optical Dichroism with Varying Helix Pitch: A First-Principles Study. Isr. J. Chem. 2022, 62, e202200053 10.1002/ijch.202200053.

[ref252] Varela S.; Gutierrez R.; Cuniberti G.; Medina E.; Mujica V. Electron Spin Polarization as a Predictor of Chiroptical Activity in Helical Molecules. arXiv:2309.00919 [cond-mat.mes-hall] 2023, 10.48550/arXiv.2309.00919.

[ref253] Wu Y.; Miao G.; Subotnik J. E. Chemical Reaction Rates for Systems with Spin-orbit Coupling and an Odd Number of Electrons: Does Berry’s Phase Lead to Meaningful Spin-dependent Nuclear Dynamics for a Two State Crossing?. J. Phys. Chem. A 2020, 124, 7355–7372. 10.1021/acs.jpca.0c04562. 32869999

[ref254] Teh H.-H.; Dou W.; Subotnik J. E. Spin Polarization through a Molecular Junction based on Nuclear Berry Curvature Effects. Phys. Rev. B 2022, 106, 184302 10.1103/PhysRevB.106.184302.

[ref255] Wu Y.; Subotnik J. E. Electronic Spin Separation Induced by Nuclear Motion Near Conical Intersections. Nat. Commun. 2021, 12, 700 10.1038/s41467-020-20831-8. 33514700 PMC7846775

[ref256] Chandran S.; Wu Y.; Teh H.-H.; Waldeck D. H.; Subotnik J. E. Electron Transfer and Spin-Orbit Coupling: How Strong are Berry Force Effects In and Out of Equilibrium in the Presence of Nuclear Friction?. J. Chem. Phys. 2022, 156, 174113 10.1063/5.0086554. 35525658

[ref257] Chandran S. S.; Wu Y.; Teh H.-H.; Waldeck D. H.; Subotnik J. E. Electron Transfer and Spin-Orbit Coupling: Can Nuclear Motion Lead to Spin Selective Rates?. J. Chem. Phys. 2022, 156, 174113 10.1063/5.0086554. 35525658

[ref258] Bian X.; Wu Y.; Rawlinson J.; Littlejohn R. G.; Subotnik J. E. Modeling Spin-dependent Nonadiabatic Dynamics with Electronic Degeneracy: a Phase-space Surface-hopping Method. J. Phys. Chem. Lett. 2022, 13, 7398–7404. 10.1021/acs.jpclett.2c01802. 35926097

[ref259] Fransson J. The Chiral Induced Spin Selectivity Effect What it is, What it is Not, and Why it Matters. Isr. J. Chem. 2022, 62, e202200046 10.1002/ijch.202200046.

[ref260] Fransson J. Vibrational Origin of Exchange Splitting and Chiral-induced Spin Selectivity. Phys. Rev. B 2020, 102, 235416 10.1103/PhysRevB.102.235416.

[ref261] Zhang L.; Hao Y.; Qin W.; Xie S.; Qu F. Chiral-induced Spin Selectivity: A Polaron Transport Model. Phys. Rev. B 2020, 102, 214303 10.1103/PhysRevB.102.214303.

[ref262] Kato A.; Yamamoto H. M.; Kishine J.-I. Chirality-induced Spin Filtering in Pseudo Jahn-Teller Molecules. Phys. Rev. B 2022, 105, 195117 10.1103/PhysRevB.105.195117.

[ref263] Barroso M.; Balduque J.; Domínguez-Adame F.; Díaz E. Spin-dependent Polaron Transport in Helical Molecules. Appl. Phys. Lett. 2022, 121, 143505 10.1063/5.0109240.

[ref264] Klein D.; Michaeli K. Giant Chirality-induced Spin Selectivity of Polarons. Phys. Rev. B 2023, 107, 045404 10.1103/PhysRevB.107.045404.

[ref265] Vittmann C.; Lim J.; Tamascelli D.; Huelga S. F.; Plenio M. B. Spin-Dependent Momentum Conservation of Electron-Phonon Scattering in Chirality-Induced Spin Selectivity. J. Phys. Chem. Lett. 2023, 14, 340–346. 10.1021/acs.jpclett.2c03224. 36625481

[ref266] Fransson J. Temperature Activated Chiral Induced Spin Selectivity. J. Chem. Phys. 2023, 159, 084115 10.1063/5.0155854. 37638628

[ref267] Fransson J. Chiral Phonon Induced Spin Polarization. Phys. Rev. Res. 2023, 5, L022039 10.1103/PhysRevResearch.5.L022039.

[ref268] Fathizadeh S. Phonon-assisted Nearly Pure Spin Current in DNA Molecular Chains: a Multifractal Analysis. Sci. Rep. 2023, 13, 21281 10.1038/s41598-023-48644-x. 38042962 PMC10693578

[ref269] Hedegård P. Spin Dynamics and Chirality Induced Spin Selectivity. J. Chem. Phys. 2023, 159, 104104 10.1063/5.0160233. 37694743

[ref270] Fransson J. Charge and Spin Dynamics and Enantioselectivity in Chiral Molecules. J. Phys. Chem. Lett. 2022, 13, 808–814. 10.1021/acs.jpclett.1c03925. 35068158 PMC8802319

[ref271] Kondou K.; Miwa S.; Miyajima D. Spontaneous Spin Selectivity in Chiral Molecules at the Interface. J. Magn. Magn. Mater. 2023, 585, 171157 10.1016/j.jmmm.2023.171157.

[ref272] Shiranzaei M.; Kalhöfer S.; Fransson J. Emergent Magnetism as a Cooperative Effect of Interactions and Reservoir. J. Phys. Chem. Lett. 2023, 14, 5119–5126. 10.1021/acs.jpclett.3c00526. 37249543 PMC10258847

[ref273] Geyer M.; Gutierrez R.; Mujica V.; Silva J.; Dianat A.; Cuniberti G. The Contribution of Intermolecular Spin Interactions to the London Dispersion Forces between Chiral Molecules. J. Chem. Phys. 2022, 156, 234106 10.1063/5.0090266. 35732515

[ref274] Hedegård P. Chiral-Induced Spin Selectivity in Capacitively Coupled Molecules. J. Phys. Chem. A 2022, 126, 3157–3166. 10.1021/acs.jpca.2c01156. 35575635

[ref275] Schweicher G.; Garbay G.; Jouclas R.; Vibert F.; Devaux F.; Geerts Y. H. Molecular Semiconductors for Logic Operations: Dead-End or Bright Future?. Adv. Mater. 2020, 32, 1905909 10.1002/adma.201905909. 31965662

[ref276] Michaeli K.; Varade V.; Naaman R.; Waldeck D. H. A New Approach Towards Spintronics - Spintronics with No Magnets. J. Phys.: Condens. Matter 2017, 29, 103002 10.1088/1361-648X/aa54a4. 28145273

[ref277] Bader S. D.; Parkin S. S. P. Spintronics. Annu. Rev. Condens. Matter Phys. 2010, 1, 71–88. 10.1146/annurev-conmatphys-070909-104123.

[ref278] Hirohata A.; Yamada K.; Nakatani Y.; Prejbeanu I. L.; Dieny B.; Pirro P.; Hillebrands B. Review on Spintronics: Principles and Device Applications. J. Magn Magn Mater. 2020, 509, 166711 10.1016/j.jmmm.2020.166711.

[ref279] Zutic I.; Fabian J.; Das Sarma S. Spintronics: Fundamentals and Applications. Rev. Mod. Phys. 2004, 76 (2), 323 10.1103/RevModPhys.76.323.

[ref280] Rikken G. L. Physics. A New Twist on Spintronics. Science 2011, 331, 864–5. 10.1126/science.1201663. 21330521

[ref281] Wolf S. A.; Awschalom D. D.; Buhrman R. A.; Daughton J. M.; von Molnar S.; Roukes M. L.; Chtchelkanova A. Y.; Treger D. M. Spintronics: A Spin-based Electronics Vision for the Future. Science 2001, 294, 1488–95. 10.1126/science.1065389. 11711666

[ref282] Baibich M. N.; Broto J. M.; Fert A.; Nguyen Van Dau F.; Petroff F.; Etienne P.; Creuzet G.; Friederich A.; Chazelas J. Giant Magnetoresistance of (001)Fe/(001)Cr Magnetic Superlattices. Phys. Rev. Lett. 1988, 61, 2472–2475. 10.1103/PhysRevLett.61.2472. 10039127

[ref283] Parkin S. S. P. Giant Magnetoresistance in Magnetic Nanostructures. Annu. Rev. Mater. Sci. 1995, 25, 357–388. 10.1146/annurev.ms.25.080195.002041.

[ref284] Julliere M. Tunneling Between Ferromagnetic-Films. Phys. Lett. A 1975, 54, 225–226. 10.1016/0375-9601(75)90174-7.

[ref285] Maekawa S.; Gafvert U. Electron-Tunneling between Ferromagnetic-Films. IEEE Trans. Magn. 1982, 18, 707–708. 10.1109/TMAG.1982.1061834.

[ref286] Akerman J. Applied Physics. Toward a Universal Memory. Science 2005, 308, 508–10. 10.1126/science.1110549. 15845842

[ref287] Gallagher W. J.; Parkin S. S. Development of the Magnetic Tunnel Junction MRAM at IBM: From First Junctions to a 16-Mb MRAM Demonstrator Chip. IBM J. Res. Dev. 2006, 50, 5–23. 10.1147/rd.501.0005.

[ref288] Katine J. A.; Fullerton E. E. Device Implications of Spin-transfer Torques. J. Magn Magn Mater. 2008, 320, 1217–1226. 10.1016/j.jmmm.2007.12.013.

[ref289] Ralph D. C.; Stiles M. D. Spin Transfer Torques. J. Magn Magn Mater. 2008, 320, 1190–1216. 10.1016/j.jmmm.2007.12.019.

[ref290] Yang S. H.; Ryu K. S.; Parkin S. Domain-wall Velocities of Up to 750 m s^–1^ Driven by Exchange-coupling Torque in Synthetic Antiferromagnets. Nat. Nanotechnol. 2015, 10, 221–6. 10.1038/nnano.2014.324. 25705867

[ref291] Dolui K.; Narayan A.; Rungger I.; Sanvito S. Efficient Spin Injection and Giant Magnetoresistance in Fe/MoS_2_/Fe Junctions. Phys. Rev. B 2014, 90, 041401 10.1103/PhysRevB.90.041401.

[ref292] Schmidt G.; Ferrand D.; Molenkamp L. W.; Filip A. T.; van Wees B. J. Fundamental Obstacle for Electrical Spin Injection from a Ferromagnetic Metal into a Diffusive Semiconductor. Phys. Rev. B 2000, 62, R4790–R4793. 10.1103/PhysRevB.62.R4790.

[ref293] van’t Erve O.; Friedman A.; Cobas E.; Li C.; Hanbicki A.; McCreary K.; Robinson J.; Jonker B. A Graphene Solution to Conductivity Mismatch: Spin Injection from Ferromagnetic Metal/Graphene Tunnel Contacts into Silicon. J. Appl. Phys. 2013, 113, 17C502 10.1063/1.4793712.

[ref294] Hosomi M.; Yamagishi H.; Yamamoto T.; Bessho K.; Higo Y.; Yamane K.; Yamada H.; Shoji M.; Hachino H.; Fukumoto C. A Novel Nonvolatile Memory with Spin Torque Transfer Magnetization Switching: Spin-RAM. IEEE InternationalElectron Devices Meeting, 2005, IEDM Technical Digest, IEEE: 2005; pp 459–462.

[ref295] Zhang Y.; Yuan H. Y.; Wang X. S.; Wang X. R. Breaking the Current Density Threshold in Spin-orbit-torque Magnetic Random Access Memory. Phys. Rev. B 2018, 97, 144416 10.1103/PhysRevB.97.144416.

[ref296] Yang S.-H.; Naaman R.; Paltiel Y.; Parkin S. S. Chiral Spintronics. Nat. Rev. Phys. 2021, 3 (5), 328–343. 10.1038/s42254-021-00302-9.

[ref297] Al-Bustami H.; Koplovitz G.; Primc D.; Yochelis S.; Capua E.; Porath D.; Naaman R.; Paltiel Y. Single Nanoparticle Magnetic Spin Memristor. Small 2018, 14, 1801249 10.1002/smll.201801249. 29952065

[ref298] Koplovitz G.; Primc D.; Ben Dor O.; Yochelis S.; Rotem D.; Porath D.; Paltiel Y. Magnetic Nanoplatelet-Based Spin Memory Device Operating at Ambient Temperatures. Adv. Mater. 2017, 29, 1606748 10.1002/adma.201606748. 28256757

[ref299] Ghosh S.; Bloom B. P.; Lu Y. Y.; Lamont D.; Waldeck D. H. Increasing the Efficiency of Water Splitting through Spin Polarization Using Cobalt Oxide Thin Film Catalysts. J. Phys. Chem. C 2020, 124, 22610–22618. 10.1021/acs.jpcc.0c07372.

[ref300] Goren N.; Das T. K.; Brown N.; Gilead S.; Yochelis S.; Gazit E.; Naaman R.; Paltiel Y. Metal Organic Spin Transistor. Nano Lett. 2021, 21, 8657–8663. 10.1021/acs.nanolett.1c01865. 34662128 PMC8859851

[ref301] Ostroverkhova O. Organic Optoelectronic Materials: Mechanisms and Applications. Chem. Rev. 2016, 116, 13279–13412. 10.1021/acs.chemrev.6b00127. 27723323

[ref302] Chen Q.; De Marco N.; Yang Y.; Song T. B.; Chen C. C.; Zhao H. X.; Hong Z. R.; Zhou H. P.; Yang Y. Under the Spotlight: The Organic-inorganic Hybrid Halide Perovskite for Optoelectronic Applications. Nano Today 2015, 10, 355–396. 10.1016/j.nantod.2015.04.009.

[ref303] Wang J.; Zhang C.; Liu H.; McLaughlin R.; Zhai Y.; Vardeny S. R.; Liu X.; McGill S.; Semenov D.; Guo H.; Tsuchikawa R.; Deshpande V. V.; Sun D.; Vardeny Z. V. Spin-optoelectronic Devices Based on Hybrid Organic-inorganic Trihalide Perovskites. Nat. Commun. 2019, 10, 129 10.1038/s41467-018-07952-x. 30631053 PMC6328620

[ref304] Fiederling R.; Keim M.; Reuscher G.; Ossau W.; Schmidt G.; Waag A.; Molenkamp L. W. Injection and Detection of a Spin-polarized Current in a Light-emitting diode. Nature 1999, 402, 787–790. 10.1038/45502.

[ref305] Ohno Y.; Young D. K.; Beschoten B.; Matsukura F.; Ohno H.; Awschalom D. D. Electrical Spin Injection in a Ferromagnetic Semiconductor Heterostructure. Nature 1999, 402, 790–792. 10.1038/45509.

[ref306] Garcia A. M.; Martínez G.; Ruiz-Carretero A. The Importance of Spin State in Chiral Supramolecular Electronics. Front. Chem. 2021, 9, 722727 10.3389/fchem.2021.722727. 34422770 PMC8371180

[ref307] Zhu Q.; Danowski W.; Mondal A. K.; Tassinari F.; van Beek C. L. F.; Heideman G. H.; Santra K.; Cohen S. R.; Feringa B. L.; Naaman R. Multistate Switching of Spin Selectivity in Electron Transport through Light-Driven Molecular Motors. Adv. Sci. 2021, 8, e2101773 10.1002/advs.202101773. PMC8456272 34292678

[ref308] Suda M.; Thathong Y.; Promarak V.; Kojima H.; Nakamura M.; Shiraogawa T.; Ehara M.; Yamamoto H. M. Light-driven Molecular Switch for Reconfigurable Spin Filters. Nat. Commun. 2019, 10, 2455 10.1038/s41467-019-10423-6. 31165729 PMC6549145

[ref309] Malatong R.; Sato T.; Kumsampao J.; Minato T.; Suda M.; Promarak V.; Yamamoto H. M. Highly Durable Spin Filter Switching Based on Self-Assembled Chiral Molecular Motor. Small 2023, 19, e2302714 10.1002/smll.202302714. 37154235

[ref310] Wang Q.; Zhu H.; Tan Y.; Hao J.; Ye T.; Tang H.; Wang Z.; Ma J.; Sun J.; Zhang T.; Zheng F.; Zhang W.; Choi H. W.; Choy W. C. H.; Wu D.; Sun X. W.; Wang K. Spin Quantum Dot Light-Emitting Diodes Enabled by 2D Chiral Perovskite with Spin-Dependent Carrier Transport. Adv. Mater. 2023, 36, 2305604 10.1002/adma.202305604. 37789724

[ref311] Ye C.; Jiang J.; Zou S.; Mi W.; Xiao Y. Core-Shell Three-Dimensional Perovskite Nanocrystals with Chiral-Induced Spin Selectivity for Room-Temperature Spin Light-Emitting Diodes. J. Am. Chem. Soc. 2022, 144, 9707–9714. 10.1021/jacs.2c01214. 35574835

[ref312] Mustaqeem M.; Chou P. T.; Kamal S.; Ahmad N.; Lin J. Y.; Lu Y. J.; Lee X. H.; Lin K. H.; Lu K. L.; Chen Y. F. Solution-Processed and Room-Temperature Spin Light-Emitting Diode Based on Quantum Dots/Chiral Metal-Organic Framework Heterostructure. Adv. Funct. Mater. 2023, 33, 2213587 10.1002/adfm.202213587.

[ref313] Sherson J. F.; Krauter H.; Olsson R. K.; Julsgaard B.; Hammerer K.; Cirac I.; Polzik E. S. Quantum Teleportation between Light and Matter. Nature 2006, 443, 557–60. 10.1038/nature05136. 17024089

[ref314] Farshchi R.; Ramsteiner M.; Herfort J.; Tahraoui A.; Grahn H. T. Optical Communication of Spin Information Between Light Emitting Diodes. Appl. Phys. Lett. 2011, 98, 162508 10.1063/1.3582917.

[ref315] Han H.; Lee Y. J.; Kyhm J.; Jeong J. S.; Han J. H.; Yang M. K.; Lee K. M.; Choi Y.; Yoon T. H.; Ju H.; Ahn S. K.; Lim J. A. High-Performance Circularly Polarized Light-sensing Near-infrared Organic Phototransistors for Optoelectronic Cryptographic Primitives. Adv. Funct. Mater. 2020, 30, 2006236 10.1002/adfm.202006236.

[ref316] Shang X.; Wan L.; Wang L.; Gao F.; Li H. Emerging Materials for Circularly Polarized Light Detection. J. Mater. Chem. C 2022, 10, 2400–2410. 10.1039/D1TC04163K.

[ref317] Yang Y.; Da Costa R. C.; Fuchter M. J.; Campbell A. J. Circularly Polarized Light Detection by a Chiral Organic Semiconductor Transistor. Nat. Photonics 2013, 7 (8), 634–638. 10.1038/nphoton.2013.176.

[ref318] Hou H. Y.; Tian S.; Ge H. R.; Chen J. D.; Li Y. Q.; Tang J. X. Recent Progress of Polarization-Sensitive Perovskite Photodetectors. Adv. Funct. Mater. 2022, 32, 2209324 10.1002/adfm.202209324.

[ref319] Yang Y.; da Costa R. C.; Fuchter M. J.; Campbell A. J. Circularly Polarized Light Detection by a Chiral Organic Semiconductor Transistor. Nat. Photonics. 2013, 7, 634–638. 10.1038/nphoton.2013.176.

[ref320] Li W.; Coppens Z. J.; Besteiro L. V.; Wang W.; Govorov A. O.; Valentine J. Circularly Polarized Light Detection with Hot Electrons in Chiral Plasmonic Metamaterials. Nat. Commun. 2015, 6, 8379 10.1038/ncomms9379. 26391292 PMC4595755

[ref321] Zhang C.; Wang X.; Qiu L. Circularly Polarized Photodetectors Based on Chiral Materials: A Review. Front. Chem. 2021, 9, 711488 10.3389/fchem.2021.711488. 34568276 PMC8455893

[ref322] Ishii A.; Miyasaka T. Direct Detection of Circular Polarized Light in Helical 1D Perovskite-based Photodiode. Sci. Adv. 2020, 6, eabd3274 10.1126/sciadv.abd3274. 33177095 PMC7673728

[ref323] Chen C.; Gao L.; Gao W.; Ge C.; Du X.; Li Z.; Yang Y.; Niu G.; Tang J. Circularly Polarized Light Detection Using Chiral Hybrid Perovskite. Nat. Commun. 2019, 10, 1927 10.1038/s41467-019-09942-z. 31028272 PMC6486588

[ref324] Wei Q.; Ning Z. J. Chiral Perovskite Spin-Optoelectronics and Spintronics: Toward Judicious Design and Application. ACS. Mater. Lett. 2021, 3, 1266–1275. 10.1021/acsmaterialslett.1c00274.

[ref325] Heberle A. P.; Ruhle W. W.; Ploog K. Quantum Beats of Electron Larmor Precession in GaAs Wells. Phys. Rev. Lett. 1994, 72, 3887–3890. 10.1103/PhysRevLett.72.3887. 10056322

[ref326] Awschalom D. D.; Kikkawa J. M. Electron Spin and Optical Coherence in Semiconductors. Phys. Today 1999, 52, 33–38. 10.1063/1.882695.

[ref327] Xiao J.; Zheng H. F.; Wang R. L.; Wang Y. L.; Hou S. C. Spin-polarized Excitons and Charge Carriers in Chiral Metal Halide Semiconductors. J. Mater. Chem. A 2022, 10, 19367–19386. 10.1039/D2TA02207A.

[ref328] Feng T.; Wang Z.; Zhang Z.; Xue J.; Lu H. Spin Selectivity in Chiral Metal-halide Semiconductors. Nanoscale 2021, 13, 18925–18940. 10.1039/D1NR06407J. 34783816

[ref329] Zhang X.; Weng W.; Li L.; Wu H.; Yao Y.; Wang Z.; Liu X.; Lin W.; Luo J. Heterogeneous Integration of Chiral Lead-Chloride Perovskite Crystals with Si Wafer for Boosted Circularly Polarized Light Detection in Solar-Blind Ultraviolet Region. Small 2021, 17, e2102884 10.1002/smll.202102884. 34480523

[ref330] Peng Y.; Liu X.; Li L.; Yao Y.; Ye H.; Shang X.; Chen X.; Luo J. Realization of vis-NIR Dual-Modal Circularly Polarized Light Detection in Chiral Perovskite Bulk Crystals. J. Am. Chem. Soc. 2021, 143, 14077–14082. 10.1021/jacs.1c07183. 34428042

[ref331] Zhang X.; Liu X.; Li L.; Ji C.; Yao Y.; Luo J. Great Amplification of Circular Polarization Sensitivity via Heterostructure Engineering of a Chiral Two-Dimensional Hybrid Perovskite Crystal with a Three-Dimensional MAPbI_3_ Crystal. ACS Cent. Sci. 2021, 7, 1261–1268. 10.1021/acscentsci.1c00649. 34345674 PMC8323243

[ref332] Zhang X.; Ye H.; Liang L.; Niu X.; Wu J.; Luo J. Direct Detection of Near-Infrared Circularly Polarized Light via Precisely Designed Chiral Perovskite Heterostructures. ACS Appl. Mater. Interfaces 2022, 14, 36781–36788. 10.1021/acsami.2c07208. 35917147

[ref333] Yao B.; Wei Q.; Yang Y.; Zhou W.; Jiang X.; Wang H.; Ma M.; Yu D.; Yang Y.; Ning Z. Symmetry-Broken 2D Lead-Tin Mixed Chiral Perovskite for High Asymmetry Factor Circularly Polarized Light Detection. Nano Lett. 2023, 23, 1938–1945. 10.1021/acs.nanolett.2c05085. 36802631

[ref334] Pan R.; Tang X.; Kan L.; Li Y.; Yu H.; Wang K. Spin-photogalvanic Effect in Chiral Lead Halide Perovskites. Nanoscale 2023, 15, 3300–3308. 10.1039/D2NR06919A. 36723152

[ref335] Eschrig M. Spin-polarized Supercurrents for Spintronics: a Review of Current Progress. Rep. Prog. Phys. 2015, 78, 104501 10.1088/0034-4885/78/10/104501. 26397456

[ref336] Eschrig M. Spin-polarized Supercurrents for Spintronics. Phys. Today 2011, 64, 43–49. 10.1063/1.3541944. 26397456

[ref337] Linder J.; Robinson J. W. A. Superconducting Spintronics. Nat. Phys. 2015, 11, 307–315. 10.1038/nphys3242.

[ref338] Bergeret F. S.; Volkov A. F.; Efetov K. B. Long-range Proximity Effects in Superconductor-ferromagnet Structures. Phys. Rev. Lett. 2001, 86, 4096–9. 10.1103/PhysRevLett.86.4096. 11328104

[ref339] Kadigrobov A.; Shekhter R. I.; Jonson M. Triplet Superconducting Proximity Effect in Inhomogeneous Magnetic Materials. Low. Temp. Phys. 2001, 27, 760–766. 10.1063/1.1401185.

[ref340] Volkov A. F.; Bergeret F. S.; Efetov K. B. Odd Triplet Superconductivity in Superconductor-Ferromagnet Multilayered Structures. Phys. Rev. Lett. 2003, 90, 117006 10.1103/PhysRevLett.90.117006. 12688960

[ref341] Bergeret F. S.; Volkov A. F.; Efetov K. B. Odd Triplet Superconductivity and Related Phenomena in Superconductor-ferromagnet Structures. Rev. Mod. Phys. 2005, 77, 1321–1373. 10.1103/RevModPhys.77.1321. 12688960

[ref342] Buzdin A. I. Proximity Effects in Superconductor-ferromagnet Heterostructures. Rev. Mod. Phys. 2005, 77, 935–976. 10.1103/RevModPhys.77.935.

[ref343] Buzdin A. I.; Bulaevskii L.; Panyukov S. Critical-current Oscillations as a Function of the Exchange Field and Thickness of the Ferromagnetic Metal (F) in an SFS Josephson Junction. JETP Lett. 1982, 35, 178–180.

[ref344] Demler E. A.; Arnold G. B.; Beasley M. R. Superconducting Proximity Effects in Magnetic Metals. Phys. Rev. B 1997, 55, 15174–15182. 10.1103/PhysRevB.55.15174.

[ref345] Kontos T.; Aprili M.; Lesueur J.; Grison X. Inhomogeneous Superconductivity Induced in a Ferromagnet by Proximity Effect. Phys. Rev. Lett. 2001, 86, 304–7. 10.1103/PhysRevLett.86.304. 11177817

[ref346] Ryazanov V.; Oboznov V.; Rusanov A. Y.; Veretennikov A.; Golubov A. A.; Aarts J. Coupling of Two Superconductors through a Ferromagnet: Evidence for a π Junction. Phys. Rev. Lett. 2001, 86, 2427 10.1103/PhysRevLett.86.2427. 11289946

[ref347] Khaire T. S.; Khasawneh M. A.; Pratt W. P. Jr.; Birge N. O. Observation of Spin-triplet Superconductivity in Co-based Josephson Junctions. Phys. Rev. Lett. 2010, 104, 137002 10.1103/PhysRevLett.104.137002. 20481906

[ref348] Robinson J. W. A.; Witt J. D. S.; Blamire M. G. Controlled Injection of Spin-Triplet Supercurrents into a Strong Ferromagnet. Science 2010, 329, 59–61. 10.1126/science.1189246. 20538913

[ref349] Keizer R. S.; Goennenwein S. T. B.; Klapwijk T. M.; Miao G. X.; Xiao G.; Gupta A. A Spin Triplet Supercurrent through the Half-metallic Ferromagnet CrO_2_. Nature 2006, 439, 825–827. 10.1038/nature04499. 16482152

[ref350] Anwar M.; Czeschka F.; Hesselberth M.; Porcu M.; Aarts J. Long-range Supercurrents through Half-metallic Ferromagnetic CrO_2_. Phys. Rev. B 2010, 82, 100501 10.1103/PhysRevB.82.100501.

[ref351] Kuznetsova V.; Gromova Y.; Martinez-Carmona M.; Purcell-Milton F.; Ushakova E.; Cherevkov S.; Maslov V.; Gun’ko Y. K. Ligand-induced Chirality and Optical Activity in Semiconductor Nanocrystals: Theory and Applications. Nanophotonics 2020, 10, 797–824. 10.1515/nanoph-2020-0473.

[ref352] Ben-Moshe A.; Teitelboim A.; Oron D.; Markovich G. Probing the Interaction of Quantum Dots with Chiral Capping Molecules Using Circular Dichroism Spectroscopy. Nano Lett. 2016, 16, 7467–7473. 10.1021/acs.nanolett.6b03143. 27960517 PMC5207631

[ref353] Ben Moshe A.; Szwarcman D.; Markovich G. Size Dependence of Chiroptical Activity in Colloidal Quantum Dots. ACS Nano 2011, 5, 9034–9043. 10.1021/nn203234b. 21967095

[ref354] Georgieva Z. N.; Bloom B. P.; Ghosh S.; Waldeck D. H. Imprinting Chirality onto the Electronic States of Colloidal Perovskite Nanoplatelets. Adv. Mater. 2018, 30, 1800097 10.1002/adma.201800097. 29700859

[ref355] Debnath G. H.; Georgieva Z. N.; Bloom B. P.; Tan S.; Waldeck D. H. Using Post-Synthetic Ligand Modification to Imprint Chirality onto the Electronic States of Cesium Lead Bromide (CsPbBr_3_) Perovskite Nanoparticles. Nanoscale 2021, 13, 15248–15256. 10.1039/D1NR04274B. 34553742

[ref356] Tabassum N.; Georgieva Z. N.; Debnath G. H.; Waldeck D. H. Size-dependent Chiro-optical Properties of CsPbBr_3_ Nanoparticles. Nanoscale 2023, 15, 2143–2151. 10.1039/D2NR06751J. 36633325

[ref357] Duran Pachon L.; Yosef I.; Markus T. Z.; Naaman R.; Avnir D.; Rothenberg G. Chiral Imprinting of Palladium with Cinchona Alkaloids. Nat. Chem. 2009, 1, 160–164. 10.1038/nchem.180. 21378830

[ref358] Gautier C.; Burgi T. Chiral Gold Nanoparticles. ChemPhysChem 2009, 10, 483–492. 10.1002/cphc.200800709. 19142928

[ref359] Lawton T. J.; Pushkarev V.; Wei D.; Lucci F. R.; Sholl D. S.; Gellman A. J.; Sykes E. C. H. Long Range Chiral Imprinting of Cu(110) by Tartaric Acid. J. Phys. Chem. C 2013, 117, 22290–22297. 10.1021/jp402015r.

[ref360] Goldsmith M.-R.; George C. B.; Zuber G.; Naaman R.; Waldeck D. H.; Wipf P.; Beratan D. N. The Chiroptical Signature of Achiral Metal Clusters Induced by Dissymmetric Adsorbates. Phys. Chem. Chem. Phys. 2006, 8, 63–67. 10.1039/B511563A. 16482245

[ref361] Alpern H.; Katzir E.; Yochelis S.; Katz N.; Paltiel Y.; Millo O. Unconventional Superconductivity Induced in Nb Films by Adsorbed Chiral Molecules. New J. Phys. 2016, 18, 113048 10.1088/1367-2630/18/11/113048.

[ref362] Alpern H.; Amundsen M.; Hartmann R.; Sukenik N.; Spuri A.; Yochelis S.; Prokscha T.; Gutkin V.; Anahory Y.; Scheer E.; Linder J.; Salman Z.; Millo O.; Paltiel Y.; Di Bernardo A. Unconventional Meissner Screening Induced by Chiral Molecules in a Conventional Superconductor. Phys. Rev. Mater. 2021, 5, 114801 10.1103/PhysRevMaterials.5.114801.

[ref363] Volosniev A. G.; Alpern H.; Paltiel Y.; Millo O.; Lemeshko M.; Ghazaryan A. Interplay Between Friction and Spin-orbit Coupling as a Source of Spin Polarization. Phys. Rev. B 2021, 104, 024430 10.1103/PhysRevB.104.024430.

[ref364] Aiello C. D.; Abendroth J. M.; Abbas M.; Afanasev A.; Agarwal S.; Banerjee A. S.; Beratan D. N.; Belling J. N.; Berche B.; Botana A.; Caram J. R.; Celardo G. L.; Cuniberti G.; Garcia-Etxarri A.; Dianat A.; Diez-Perez I.; Guo Y.; Gutierrez R.; Herrmann C.; Hihath J.; Kale S.; Kurian P.; Lai Y. C.; Liu T.; Lopez A.; Medina E.; Mujica V.; Naaman R.; Noormandipour M.; Palma J. L.; Paltiel Y.; Petuskey W.; Ribeiro-Silva J. C.; Saenz J. J.; Santos E. J. G.; Solyanik-Gorgone M.; Sorger V. J.; Stemer D. M.; Ugalde J. M.; Valdes-Curiel A.; Varela S.; Waldeck D. H.; Wasielewski M. R.; Weiss P. S.; Zacharias H.; Wang Q. H. A Chirality-Based Quantum Leap. ACS Nano 2022, 16, 4989–5035. 10.1021/acsnano.1c01347. 35318848 PMC9278663

[ref365] Olshansky J. H.; Krzyaniak M. D.; Young R. M.; Wasielewski M. R. Photogenerated Spin-Entangled Qubit (Radical) Pairs in DNA Hairpins: Observation of Spin Delocalization and Coherence. J. Am. Chem. Soc. 2019, 141, 2152–2160. 10.1021/jacs.8b13155. 30636401

[ref366] Fridman H. T.; Dehnel J.; Yochelis S.; Lifshitz E.; Paltiel Y. Spin-exciton Delocalization Enhancement in Multilayer Chiral Linker/Quantum Dot Structures. J. Phys. Chem. Lett. 2019, 10, 3858–3862. 10.1021/acs.jpclett.9b01433. 31241942

[ref367] Fridman H. T.; Levy H. M.; Meir A.; Casotto A.; Malkinson R.; Dehnel J.; Yochelis S.; Lifshitz E.; Bar-Gill N.; Collini E.; Paltiel Y. Ultrafast Coherent Delocalization Revealed in Multilayer QDs Under a Chiral Potential. J. Phys. Chem. Lett. 2023, 14, 2234–2240. 10.1021/acs.jpclett.2c03743. 36820505 PMC11139383

[ref368] Cao Y.; Xing G. Z.; Lin H.; Zhang N.; Zheng H. Z.; Wang K. Y. Prospect of Spin-Orbitronic Devices and Their Applications. iScience 2020, 23, 101614 10.1016/j.isci.2020.101614. 33089103 PMC7559259

[ref369] Woo S.; Litzius K.; Kruger B.; Im M. Y.; Caretta L.; Richter K.; Mann M.; Krone A.; Reeve R. M.; Weigand M.; Agrawal P.; Lemesh I.; Mawass M. A.; Fischer P.; Klaui M.; Beach G. R. S. D. Observation of Room-temperature Magnetic Skyrmions and their Current-driven Dynamics in Ultrathin Metallic Ferromagnets. Nat. Mater. 2016, 15, 501–506. 10.1038/nmat4593. 26928640

[ref370] Gibertini M.; Koperski M.; Morpurgo A. F.; Novoselov K. S. Magnetic 2D Materials and Heterostructures. Nat. Nanotechnol. 2019, 14, 408–419. 10.1038/s41565-019-0438-6. 31065072

[ref371] Wang Q. H.; Bedoya-Pinto A.; Blei M.; Dismukes A. H.; Hamo A.; Jenkins S.; Koperski M.; Liu Y.; Sun Q.-C.; Telford E. J.; Kim H. H.; Augustin M.; Vool U.; Yin J.-X.; Li L. H.; Falin A.; Dean C. R.; Casanova F.; Evans R. F. L.; Chshiev M.; Mishchenko A.; Petrovic C.; He R.; Zhao L.; Tsen A. W.; Gerardot B. D.; Brotons-Gisbert M.; Guguchia Z.; Roy X.; Tongay S.; Wang Z.; Hasan M. Z.; Wrachtrup J.; Yacoby A.; Fert A.; Parkin S.; Novoselov K. S.; Dai P.; Balicas L.; Santos E. J. G. The Magnetic Genome of Two-dimensional van der Waals Materials. ACS Nano 2022, 16, 6960–7079. 10.1021/acsnano.1c09150. 35442017 PMC9134533

[ref372] Nakatsuji S.; Kiyohara N.; Higo T. Large Anomalous Hall Effect in a Non-collinear Antiferromagnet at Room Temperature. Nature 2015, 527, 212–215. 10.1038/nature15723. 26524519

[ref373] Marti X.; Fina I.; Frontera C.; Liu J.; Wadley P.; He Q.; Paull R. J.; Clarkson J. D.; Kudrnovsky J.; Turek I.; Kunes J.; Yi D.; Chu J. H.; Nelson C. T.; You L.; Arenholz E.; Salahuddin S.; Fontcuberta J.; Jungwirth T.; Ramesh R. Room-temperature Antiferromagnetic Memory Resistor. Nat. Mater. 2014, 13, 367–74. 10.1038/nmat3861. 24464243

[ref374] Grinolds M. S.; Maletinsky P.; Hong S.; Lukin M. D.; Walsworth R. L.; Yacoby A. Quantum Control of Proximal Spins using Nanoscale Magnetic Resonance Imaging. Nat. Phys. 2011, 7, 687–692. 10.1038/nphys1999.

[ref375] Wan L.; Liu Y.; Fuchter M. J.; Yan B. Anomalous Circularly Polarized Light Emission in Organic Light-emitting Diodes Caused by Orbital-momentum Locking. Nat. Photonics. 2023, 17, 193–199. 10.1038/s41566-022-01113-9.

[ref376] Abendroth J. M.; Stemer D. M.; Bloom B. P.; Roy P.; Naaman R.; Waldeck D. H.; Weiss P. S.; Mondal P. C. Spin Selectivity in Photoinduced Charge-Transfer Mediated by Chiral Molecules. ACS Nano 2019, 13, 4928–4946. 10.1021/acsnano.9b01876. 31016968

[ref377] Fiebig M.; Lottermoser T.; Meier D.; Trassin M. The Evolution of Multiferroics. Nat. Rev. Mater. 2016, 1, 1–14. 10.1038/natrevmats.2016.46.

[ref378] Ma J.; Hu J.; Li Z.; Nan C. W. Recent Progress in Multiferroic Magnetoelectric Composites: from Bulk to Thin Films. Adv. Mater. 2011, 23, 1062–87. 10.1002/adma.201003636. 21294169

[ref379] Van Aken B. B.; Rivera J. P.; Schmid H.; Fiebig M. Observation of Ferrotoroidic Domains. Nature 2007, 449, 702–5. 10.1038/nature06139. 17928856

[ref380] Kimura T.; Goto T.; Shintani H.; Ishizaka K.; Arima T.; Tokura Y. Magnetic Control of Ferroelectric Polarization. Nature 2003, 426, 55–8. 10.1038/nature02018. 14603314

[ref381] Cheong S. W.; Mostovoy M. Multiferroics: a Magnetic Twist for Ferroelectricity. Nat. Mater. 2007, 6, 13–20. 10.1038/nmat1804. 17199121

[ref382] Bhoi K.; Mohanty H. S.; Ravikant; Abdullah M. F.; Pradhan D. K.; Babu S. N.; Singh A. K.; Vishwakarma P. N.; Kumar A.; Thomas R.; Pradhan D. K. Unravelling the Nature of Magneto-electric Coupling in Room Temperature Multiferroic Particulate PbFe_0.5_Nb_0.5_O_3_-Co_0.6_Zn_0.4_Fe_1.7_Mn_0.3_O_4_ Composites. Sci. Rep. 2021, 11, 3149 10.1038/s41598-021-82399-7. 33542285 PMC7862596

[ref383] Ruff A.; Lunkenheimer P.; von Nidda H. A. K.; Widmann S.; Prokofiev A.; Svistov L.; Loidl A.; Krohns S. Chirality-driven Ferroelectricity in LiCuVO_4_. Npj Quantum Mater. 2019, 4, 24 10.1038/s41535-019-0163-2.

[ref384] Hu Y.; Florio F.; Chen Z.; Phelan W. A.; Siegler M. A.; Zhou Z.; Guo Y.; Hawks R.; Jiang J.; Feng J.; Zhang L.; Wang B.; Wang Y.; Gall D.; Palermo E. F.; Lu Z.; Sun X.; Lu T. M.; Zhou H.; Ren Y.; Wertz E.; Sundararaman R.; Shi J. A Chiral Switchable Photovoltaic Ferroelectric 1D Perovskite. Sci. Adv. 2020, 6, eaay4213 10.1126/sciadv.aay4213. 32158941 PMC7048427

[ref385] Gao W.; Zhang Z.; Li P. F.; Tang Y. Y.; Xiong R. G.; Yuan G.; Ren S. Chiral Molecular Ferroelectrics with Polarized Optical Effect and Electroresistive Switching. ACS Nano 2017, 11, 11739–11745. 10.1021/acsnano.7b07090. 29136365

[ref386] Katsura H.; Nagaosa N.; Balatsky A. V. Spin Current and Magnetoelectric Effect in Noncollinear Magnets. Phys. Rev. Lett. 2005, 95, 057205 10.1103/PhysRevLett.95.057205. 16090916

[ref387] Sergienko I. A.; Dagotto E. Role of the Dzyaloshinskii-Moriya Interaction in Multiferroic Perovskites. Phys. Rev. B 2006, 73, 094434 10.1103/PhysRevB.73.094434.

[ref388] Dzyaloshinsky I. A Thermodynamic Theory of Weak Ferromagnetism of Antiferromagnetics. J. Phys. Chem. Solids 1958, 4, 241–255. 10.1016/0022-3697(58)90076-3.

[ref389] Xu Y.; Mi W. Chiral-induced Spin Selectivity in Biomolecules, Hybrid Organic-inorganic Perovskites and Inorganic Materials: a Comprehensive Review on Recent Progress. Mater. Horiz. 2023, 10, 1924–1955. 10.1039/D3MH00024A. 36989068

[ref390] Burner U.; Obinger C. Transient-state and Steady-state Kinetics of the Oxidation of Aliphatic and Aromatic Thiols by Horseradish Peroxidase. FEBS Lett. 1997, 411, 269–74. 10.1016/S0014-5793(97)00713-8. 9271219

[ref391] Hager G.; Brolo A. G. Protonation and Deprotonation of Cysteine and Cystine Monolayers Probed by Impedance Spectroscopy. J. Electroanal. Chem. 2009, 625, 109–116. 10.1016/j.jelechem.2008.10.026.

[ref392] Tassinari F.; Steidel J.; Paltiel S.; Fontanesi C.; Lahav M.; Paltiel Y.; Naaman R. Enantioseparation by Crystallization using Magnetic Substrates. Chem. Sci. 2019, 10, 5246–5250. 10.1039/C9SC00663J. 31191879 PMC6540959

[ref393] Bhowmick D.; Sang Y. T.; Santra K.; Halbauer M.; Capua E.; Paltiel Y.; Naaman R.; Tassinari F. Simultaneous High-Purity Enantiomeric Resolution of Conglomerates Using Magnetic Substrates. Cryst. Growth Des. 2021, 21, 2925–2931. 10.1021/acs.cgd.1c00093.

[ref394] Ozturk S. F.; Liu Z.; Sutherland J. D.; Sasselov D. D. Origin of Biological Homochirality by Crystallization of an RNA Precursor on a Magnetic Surface. Sci. Adv. 2023, 9, eadg8274 10.1126/sciadv.adg8274. 37285423 PMC10246896

[ref395] Metzger T. S.; Tokatly Y.; Avigad E.; Yochelis S.; Paltiel Y. Selective Enantiomer Purification Using Magnetic Oriented Interacting Microparticles. Sep. Purif. Technol. 2020, 239, 116501 10.1016/j.seppur.2020.116501.

[ref396] Seitz L. C.; Dickens C. F.; Nishio K.; Hikita Y.; Montoya J.; Doyle A.; Kirk C.; Vojvodic A.; Hwang H. Y.; Norskov J. K.; Jaramillo T. F. A Highly Active and Stable IrO_x_/SrIrO_3_ Catalyst for the Oxygen Evolution Reaction. Science 2016, 353, 1011–1014. 10.1126/science.aaf5050. 27701108

[ref397] Kong F. Q. Synthesis of Rod and Beadlike Co_3_O_4_ and Bi-functional Properties as Air/Oxygen Electrode Materials. Electrochim. Acta 2012, 68, 198–201. 10.1016/j.electacta.2012.02.064.

[ref398] Zou L.; Cheng J. F.; Jiang Y. X.; Gong Y. P.; Chi B.; Pu J.; Jian L. Spinel MnCo_2_O_4_ Nanospheres as an Effective Cathode Electrocatalyst for Rechargeable Lithium-oxygen batteries. Rsc Adv. 2016, 6, 31248–31255. 10.1039/C5RA27615B.

[ref399] Frydendal R.; Paoli E. A.; Chorkendorff I.; Rossmeisl J.; Stephens I. E. L. Toward an Active and Stable Catalyst for Oxygen Evolution in Acidic Media: Ti-Stabilized MnO_2_. Adv. Energy Mater. 2015, 5, 1500991 10.1002/aenm.201500991.

[ref400] Debe M. K. Electrocatalyst Approaches and Challenges for Automotive Fuel Cells. Nature 2012, 486, 43–51. 10.1038/nature11115. 22678278

[ref401] Cao R.; Lee J. S.; Liu M. L.; Cho J. Recent Progress in Non-Precious Catalysts for Metal-Air Batteries. Adv. Energy Mater. 2012, 2, 816–829. 10.1002/aenm.201200013.

[ref402] Meng Y.; Zhang X.; Hung W. H.; He J.; Tsai Y. S.; Kuang Y.; Kenney M. J.; Shyue J. J.; Liu Y.; Stone K. H.; Zheng X.; Suib S. L.; Lin M. C.; Liang Y.; Dai H. Highly Active Oxygen Evolution Integrated with Efficient CO_2_ to CO Electroreduction. Proc. Natl. Acad. Sci. U.S.A. 2019, 116, 23915–23922. 10.1073/pnas.1915319116. 31723041 PMC6883796

[ref403] Kauffman D. R.; Alfonso D.; Tafen D.; Lekse J.; Wang C. J.; Deng X. Y.; Lee J.; Jang H.; Lee J. S.; Kumar S.; Matranga C. Electrocatalytic Oxygen Evolution with an Atomically Precise Nickel Catalyst. ACS Catal. 2016, 6, 1225–1234. 10.1021/acscatal.5b02633.

[ref404] Liang Y. C.; Lihter M.; Lingenfelder M. Spin-Control in Electrocatalysis for Clean Energy. Isr. J. Chem. 2022, 62, e202200052 10.1002/ijch.202200052.

[ref405] Mtangi W.; Kiran V.; Fontanesi C.; Naaman R. Role of the Electron Spin Polarization in Water Splitting. J. Phys. Chem. Lett. 2015, 6, 4916–22. 10.1021/acs.jpclett.5b02419. 26615833 PMC4685426

[ref406] Seabold J. A.; Choi K.-S. Effect of a Cobalt-Based Oxygen Evolution Catalyst on the Stability and the Selectivity of Photo-Oxidation Reactions of a WO_3_ Photoanode. Chem. Mater. 2011, 23, 1105–1112. 10.1021/cm1019469.

[ref407] Adelizzi B.; Rosch A. T.; van Rijen D. J.; Martire R. S.; Esiner S.; Lutz M.; Palmans A. R. A.; Meijer E. W. Chiral Aggregates of Triphenylamine-Based Dyes for Depleting the Production of Hydrogen Peroxide in the Photochemical Water-Splitting Process. Helv. Chim. Acta 2019, 102, e1900065 10.1002/hlca.201900065.

[ref408] Gazzotti M.; Stefani A.; Bonechi M.; Giurlani W.; Innocenti M.; Fontanesi C. Influence of Chiral Compounds on the Oxygen Evolution Reaction (OER) in the Water Splitting Process. Molecules 2020, 25, 3988 10.3390/molecules25173988. 32883035 PMC7504774

[ref409] Bhartiya P. K.; Srivastava M.; Mishra D. Chiral-induced Enhanced Electrocatalytic Behaviour of Cysteine Coated Bifunctional Au-Ni Bilayer Thin Film Device for Water Splitting Application. Int. J. Hydrogen Energy 2022, 47, 42160–42170. 10.1016/j.ijhydene.2021.08.219.

[ref410] Zhang W. Y.; Wang W.; Hu Y. F.; Guan H. M.; Hao L. Y. Take a Cue from Nature: Promoting Electrocatalytic Watersplitting with a Helping Hand of Hemoglobin. Int. J. Hydrogen Energy 2021, 46, 3504–3509. 10.1016/j.ijhydene.2020.10.154.

[ref411] Lee H.; Ma S.; Oh S.; Tan J.; Lee C. U.; Son J.; Park Y. S.; Yun J.; Jang G.; Moon J. Chirality-Induced Spin Selectivity of Chiral 2D Perovskite Enabling Efficient Spin-Dependent Oxygen Evolution Reaction. Small 2023, 19, 2304166 10.1002/smll.202304166. 37282813

[ref412] Feng T.; Chen W.; Xue J.; Cao F.; Chen Z.; Ye J.; Xiao C.; Lu H. Spin Polarization of Chiral Amorphous Fe-Ni Electrocatalysts Enabling Efficient Electrochemical Oxygen Evolution. Adv. Funct. Mater. 2023, 33, 2215051 10.1002/adfm.202215051.

[ref413] Mingoes C. J.; Schroeder B. C.; Jorge Sobrido A. B. Electron Spin Selective Iridium Electrocatalysts for the Oxygen Evolution Reaction. ACS Mater. Au 2023. 10.1021/acsmaterialsau.3c00084 PMC10941284 38496043

[ref414] Ai M.; Pan L.; Shi C.; Huang Z. F.; Zhang X.; Mi W.; Zou J. J. Spin Selection in Atomic-level Chiral Metal Oxide for Photocatalysis. Nat. Commun. 2023, 14, 4562 10.1038/s41467-023-40367-x. 37507418 PMC10382512

[ref415] Jin Y.; Fu W.; Wen Z.; Tan L.; Chen Z.; Wu H.; Wang P.-p. Chirality Engineering of Colloidal Copper Oxide Nanostructures for Tailored Spin-Polarized Catalysis. J. Am. Chem. Soc. 2023, 146, 2798 10.1021/jacs.3c12965. 38145451

[ref416] Vadakkayil A.; Clever C.; Kunzler K. N.; Tan S.; Bloom B. P.; Waldeck D. H. Chiral Electrocatalysts Eclipse Water Splitting Metrics through Spin Control. Nat. Commun. 2023, 14, 1067 10.1038/s41467-023-36703-w. 36828840 PMC9958132

[ref417] Jiao Y.; Sharpe R.; Lim T.; Niemantsverdriet J. W. H.; Gracia J. Photosystem II Acts as a Spin-Controlled Electron Gate During Oxygen Formation and Evolution. J. Am. Chem. Soc. 2017, 139, 16604–16608. 10.1021/jacs.7b07634. 29064697

[ref418] Zhang W. Y.; Wang W.; Hu Y. F.; Guan H. M.; Yang X. L.; Hao L. Y. Chiral CuO@Ni with Continuous Macroporous Framework and its High Catalytic Activity for Electrochemical Water Oxidation. Int. J. Hydrogen Energy 2021, 46, 8922–8931. 10.1016/j.ijhydene.2020.12.210.

[ref419] Feng T. L.; Chen W. H.; Xue J.; Cao F. F.; Chen Z. W.; Ye J. C.; Xiao C. X.; Lu H. P. Spin Polarization of Chiral Amorphous Fe-Ni Electrocatalysts Enabling Efficient Electrochemical Oxygen Evolution. Adv. Funct. Mater. 2023, 33, 2215051 10.1002/adfm.202215051.

[ref420] Trojanowicz M.; Bobrowski K.; Szreder T.; Bojanowska-Czajka A., Gamma-ray, X-ray and Electron Beam Based Processes. In Advanced Oxidation Processes for Waste Water Treatment, Ameta S. C.; Ameta R., Eds. Academic Press: 2018; pp 257–331.

[ref421] Ren X.; Wu T.; Sun Y.; Li Y.; Xian G.; Liu X.; Shen C.; Gracia J.; Gao H. J.; Yang H.; Xu Z. J. Spin-polarized Oxygen Evolution Reaction Under Magnetic Field. Nat. Commun. 2021, 12, 2608 10.1038/s41467-021-22865-y. 33972558 PMC8110536

[ref422] Man I. C.; Su H. Y.; Calle-Vallejo F.; Hansen H. A.; Martinez J. I.; Inoglu N. G.; Kitchin J.; Jaramillo T. F.; Norskov J. K.; Rossmeisl J. Universality in Oxygen Evolution Electrocatalysis on Oxide Surfaces. Chem. Catal. Chem. 2011, 3, 1159–1165. 10.1002/cctc.201000397.

[ref423] Seh Z. W.; Kibsgaard J.; Dickens C. F.; Chorkendorff I.; Norskov J. K.; Jaramillo T. F. Combining Theory and Experiment in Electrocatalysis: Insights into Materials Design. Science 2017, 355, eaad4998 10.1126/science.aad4998. 28082532

[ref424] Gracia J. Spin Dependent Interactions Catalyse the Oxygen Electrochemistry. Phys. Chem. Chem. Phys. 2017, 19, 20451–20456. 10.1039/C7CP04289B. 28745744

[ref425] Wu T.; Ren X.; Sun Y.; Sun S.; Xian G.; Scherer G. G.; Fisher A. C.; Mandler D.; Ager J. W.; Grimaud A.; Wang J.; Shen C.; Yang H.; Gracia J.; Gao H. J.; Xu Z. J. Spin Pinning Effect to Reconstructed Oxyhydroxide Layer on Ferromagnetic Oxides for Enhanced Water Oxidation. Nat. Commun. 2021, 12, 3634 10.1038/s41467-021-23896-1. 34131143 PMC8206068

[ref426] Wu T. Z.; Xu Z. C. J. Oxygen Evolution in Spin-sensitive Pathways. Curr. Opin Electroche 2021, 30, 100804 10.1016/j.coelec.2021.100804.

[ref427] Jung S.; McCrory C. C. L.; Ferrer I. M.; Peters J. C.; Jaramillo T. F. Benchmarking Nanoparticulate Metal Oxide Electrocatalysts for the Alkaline Water Oxidation Reaction. J. Mater. Chem. A 2016, 4, 3068–3076. 10.1039/C5TA07586F.

[ref428] Sang Y.; Tassinari F.; Santra K.; Zhang W.; Fontanesi C.; Bloom B. P.; Waldeck D. H.; Fransson J.; Naaman R. Chirality Enhances Oxygen Reduction. Proc. Natl. Acad. Sci. U.S.A. 2022, 119, e2202650119 10.1073/pnas.2202650119. 35858429 PMC9335305

[ref429] Scarpetta-Pizo L.; Venegas R.; Barrías P.; Muñoz-Becerra K.; Vilches-Labbé N.; Mura F.; Méndez-Torres A. M.; Ramírez-Tagle R.; Toro-Labbé A.; Hevia S.; Zagal J. H.; Oñate R.; Aspée A.; Ponce I. Electron Spin-Dependent Electrocatalysis for the Oxygen Reduction Reaction in a Chiro-Self-Assembled Iron Phthalocyanine Device. Angew. Chem., Int. Ed. 2023, 63, e202315146 10.1002/anie.202315146. 37953459

[ref430] Gupta A.; Kumar A.; Bhowmick D. K.; Fontanesi C.; Paltiel Y.; Fransson J.; Naaman R. Does Coherence Affect the Multielectron Oxygen Reduction Reaction?. J. Phys. Chem. Lett. 2023, 14, 9377–9384. 10.1021/acs.jpclett.3c02594. 37824289 PMC10614294

[ref431] Bloom B. P.; Lu Y.; Metzger T.; Yochelis S.; Paltiel Y.; Fontanesi C.; Mishra S.; Tassinari F.; Naaman R.; Waldeck D. H. Asymmetric Reactions Induced by Electron Spin Polarization. Phys. Chem. Chem. Phys. 2020, 22, 21570–21582. 10.1039/D0CP03129A. 32697241

[ref432] Gazzotti M.; Arnaboldi S.; Grecchi S.; Giovanardi R.; Cannio M.; Pasquali L.; Giacomino A.; Abollino O.; Fontanesi C. Spin-dependent Electrochemistry: Enantio-selectivity Driven by Chiral-induced Spin Selectivity Effect. Electrochim. Acta 2018, 286, 271–278. 10.1016/j.electacta.2018.08.023.

[ref433] Metzger T. S.; Siam R.; Kolodny Y.; Goren N.; Sukenik N.; Yochelis S.; Abu-Reziq R.; Avnir D.; Paltiel Y. Dynamic Spin-Controlled Enantioselective Catalytic Chiral Reactions. J. Phys. Chem. Lett. 2021, 12, 5469–5472. 10.1021/acs.jpclett.1c01518. 34085834

[ref434] Tassinari F.; Amsallem D.; Bloom B. P.; Lu Y. Y.; Bedi A.; Waldeck D. H.; Gidron O.; Naaman R. Spin-Dependent Enantioselective Electropolymerization. J. Phys. Chem. C 2020, 124, 20974–20980. 10.1021/acs.jpcc.0c06238.

[ref435] Monzon L. M. A.; Coey J. M. D. Magnetic Fields in Electrochemistry: The Lorentz Force. A Mini-review. Electrochem. Commun. 2014, 42, 38–41. 10.1016/j.elecom.2014.02.006.

[ref436] Hedström S.; dos Santos E. C.; Liu C.; Chan K.; Abild-Pedersen F.; Pettersson L. G. M. Spin Uncoupling in Chemisorbed OCCO and CO_2_: Two High-Energy Intermediates in Catalytic CO_2_ Reduction. J. Phys. Chem. C 2018, 122, 12251–12258. 10.1021/acs.jpcc.8b02165.

[ref437] Player T. C.; Hore P. J. Source of Magnetic Field Effects on the Electrocatalytic Reduction of CO_2_. J. Chem. Phys. 2020, 153, 084303 10.1063/5.0021643. 32872863

[ref438] Pan H.; Jiang X.; Wang X.; Wang Q.; Wang M.; Shen Y. Effective Magnetic Field Regulation of the Radical Pair Spin States in Electrocatalytic CO_2_ Reduction. J. Phys. Chem. Lett. 2020, 11, 48–53. 10.1021/acs.jpclett.9b03146. 31821005

[ref439] Cao A.; Bukas V. J.; Shadravan V.; Wang Z.; Li H.; Kibsgaard J.; Chorkendorff I.; Norskov J. K. A Spin Promotion Effect in Catalytic Ammonia Synthesis. Nat. Commun. 2022, 13, 2382 10.1038/s41467-022-30034-y. 35501341 PMC9061734

[ref440] Zhao Z.; Wang D.; Gao R.; Wen G.; Feng M.; Song G.; Zhu J.; Luo D.; Tan H.; Ge X.; Zhang W.; Zhang Y.; Zheng L.; Li H.; Chen Z. Magnetic-Field-Stimulated Efficient Photocatalytic N_2_ Fixation over Defective BaTiO_3_ Perovskites. Angew. Chem., Int. Ed. Engl. 2021, 60, 11910–11918. 10.1002/anie.202100726. 33605019

[ref441] Lielmezs J.; Morgan J. P. Magneto-Catalytic Effect in Ethylene Hydrogenation Reaction. Chem. Eng. Sci. 1967, 22, 781–791. 10.1016/0009-2509(67)80092-7.

[ref442] Thomas N.; Dionysiou D. D.; Pillai S. C. Heterogeneous Fenton Catalysts: A Review of Recent Advances. J. Hazard Mater. 2021, 404, 124082 10.1016/j.jhazmat.2020.124082. 33069994 PMC7530584

[ref443] Biz C.; Fianchini M.; Gracia J. Strongly Correlated Electrons in Catalysis: Focus on Quantum Exchange. ACS Catal. 2021, 11, 14249–14261. 10.1021/acscatal.1c03135.

[ref444] Biz C.; Gracia J.; Fianchini M. Review on Magnetism in Catalysis: From Theory to PEMFC Applications of 3d Metal Pt-Based Alloys. Int. J. Mol. Sci. 2022, 23, 14768 10.3390/ijms232314768. 36499096 PMC9739051

[ref445] Monzon L. M. A.; Coey J. M. D. Magnetic Fields in Electrochemistry: The Kelvin Force. A Mini-review. Electrochem. Commun. 2014, 42, 42–45. 10.1016/j.elecom.2014.02.005.

[ref446] Bhargava S. S.; Azmoodeh D.; Chen X. Y.; Cofell E. R.; Esposito A. M.; Verma S.; Gewirth A. A.; Kenis P. J. A. Decreasing the Energy Consumption of the CO_2_ Electrolysis Process Using a Magnetic Field. ACS Energy Lett. 2021, 6, 2427–2433. 10.1021/acsenergylett.1c01029.

[ref447] Kodaimati M. S.; Gao R.; Root S. E.; Whitesides G. M. Magnetic Fields Enhance Mass Transport during Electrocatalytic Reduction of CO_2_. Chem. Catal. 2022, 2, 797–815. 10.1016/j.checat.2022.01.023.

[ref448] Ghosh M.; Shinde V. S.; Rueping M. A Review of Asymmetric Synthetic Organic Electrochemistry and Electrocatalysis: Concepts, Applications, Recent Developments and Future Directions. Beilstein J. Org. Chem. 2019, 15, 2710–2746. 10.3762/bjoc.15.264. 31807206 PMC6880813

[ref449] Yamamoto K.; Kuriyama M.; Onomura O. Asymmetric Electrosynthesis: Recent Advances in Catalytic Transformations. Curr. Opin. Electrochem. 2021, 28, 100714 10.1016/j.coelec.2021.100714.

[ref450] Fay T. P.; Limmer D. T. Origin of Chirality Induced Spin Selectivity in Photoinduced Electron Transfer. Nano Lett. 2021, 21, 6696–6702. 10.1021/acs.nanolett.1c02370. 34291928

[ref451] Steiner U. E.; Ulrich T. Magnetic-Field Effects in Chemical-Kinetics and Related Phenomena. Chem. Rev. 1989, 89, 51–147. 10.1021/cr00091a003.

[ref452] Tiwari Y.; Poonia V. S. Role of Chiral-induced Spin selectivity in the Radical Pair Mechanism of Avian Magnetoreception. Phys. Rev. E 2022, 106, 064409 10.1103/PhysRevE.106.064409. 36671157

[ref453] Tiwari Y.; Poonia V. S. Quantum Coherence Enhancement by the Chirality-induced Spin Selectivity Effect in the Radical-pair Mechanism. Phys. Rev. A 2023, 107, 052406 10.1103/PhysRevA.107.052406.

[ref454] Marsh J. A.; Teichmann S. A. Structure, Dynamics, Assembly, and Evolution of Protein Complexes. Annu. Rev. Biochem. 2015, 84, 551–75. 10.1146/annurev-biochem-060614-034142. 25494300

[ref455] Wilson W. D. Analyzing Biomolecular Interactions. Science 2002, 295, 2103–5. 10.1126/science.295.5562.2103. 11896282

[ref456] Wagner J. R.; Lee C. T.; Durrant J. D.; Malmstrom R. D.; Feher V. A.; Amaro R. E. Emerging Computational Methods for the Rational Discovery of Allosteric Drugs. Chem. Rev. 2016, 116, 6370–6390. 10.1021/acs.chemrev.5b00631. 27074285 PMC4901368

[ref457] Christensen A. S.; Kubar T.; Cui Q.; Elstner M. Semiempirical Quantum Mechanical Methods for Noncovalent Interactions for Chemical and Biochemical Applications. Chem. Rev. 2016, 116, 5301–5337. 10.1021/acs.chemrev.5b00584. 27074247 PMC4867870

[ref458] Wilchek M.; Bayer E. A.; Livnah O. Essentials of Biorecognition: The (Strept) Avidin-biotin System as a Model for Protein-protein and Protein-ligand Interaction. Immunol. Lett. 2006, 103, 27–32. 10.1016/j.imlet.2005.10.022. 16325268

[ref459] Williams D. H.; Stephens E.; O’Brien D. P.; Zhou M. Understanding Noncovalent Interactions: Ligand Binding Energy and Catalytic Efficiency from Ligand-induced Reductions in Motion Within Receptors and Enzymes. Angew. Chem., Int. Ed. Engl. 2004, 43, 6596–6616. 10.1002/anie.200300644. 15593167

[ref460] Kapon Y.; Zhu Q.; Yochelis S.; Naaman R.; Gutierrez R.; Cuniberti G.; Paltiel Y.; Mujica V. Probing Chiral Discrimination in Biological Systems using Atomic Force Microscopy: The Role of van der Waals and Exchange Interactions. J. Chem. Phys. 2023, 159, 224702 10.1063/5.0171742. 38063226

[ref461] Liu J.; Nussinov R. Allostery: An Overview of Its History, Concepts, Methods, and Applications. PLoS Comput. Biol. 2016, 12, e1004966 10.1371/journal.pcbi.1004966. 27253437 PMC4890769

[ref462] Monod J.; Wyman J.; Changeux J.-P. On the Nature of Allosteric Transitions: a Plausible Model. J. Mol. Biol. 1965, 12, 88–118. 10.1016/S0022-2836(65)80285-6. 14343300

[ref463] Laskowski R. A.; Gerick F.; Thornton J. M. The Structural Basis of Allosteric Regulation in Proteins. FEBS Lett. 2009, 583, 1692–8. 10.1016/j.febslet.2009.03.019. 19303011

[ref464] Motlagh H. N.; Wrabl J. O.; Li J.; Hilser V. J. The Ensemble Nature of Allostery. Nature 2014, 508, 331–9. 10.1038/nature13001. 24740064 PMC4224315

[ref465] Gunasekaran K.; Ma B.; Nussinov R. Is Allostery an Intrinsic Property of All Dynamic Proteins?. Proteins: Struct. Funct. Genet. 2004, 57, 433–443. 10.1002/prot.20232. 15382234

[ref466] Ghosh S.; Banerjee-Ghosh K.; Levy D.; Scheerer D.; Riven I.; Shin J.; Gray H. B.; Naaman R.; Haran G. Control of Protein Activity by Photoinduced Spin Polarized Charge Reorganization. Proc. Natl. Acad. Sci. U.S.A. 2022, 119, e2204735119 10.1073/pnas.2204735119. 35994638 PMC9436351

[ref467] Michaeli K.; Kantor-Uriel N.; Naaman R.; Waldeck D. H. The Electron’s Spin and Molecular Chirality - How Are They Related and How Do They Affect Life Processes?. Chem. Soc. Rev. 2016, 45, 6478–6487. 10.1039/C6CS00369A. 27734046

[ref468] Ozturk S. F.; Sasselov D. D. On the Origins of Life’s Homochirality: Inducing Enantiomeric Excess with Spin-polarized Electrons. Proc. Natl. Acad. Sci. U.S.A. 2022, 119, e2204765119 10.1073/pnas.2204765119. 35787048 PMC9282223

[ref469] Sasselov D. D.; Grotzinger J. P.; Sutherland J. D. The Origin of Life as a Planetary Phenomenon. Sci. Adv. 2020, 6, eaax3419 10.1126/sciadv.aax3419. 32076638 PMC7002131

[ref470] Patel B. H.; Percivalle C.; Ritson D. J.; Duffy C. D.; Sutherland J. D. Common Origins of RNA, Protein and Lipid Precursors in a Cyanosulfidic Protometabolism. Nat. Chem. 2015, 7, 301–7. 10.1038/nchem.2202. 25803468 PMC4568310

[ref471] Blackmond D. G. Autocatalytic Models for the Origin of Biological Homochirality. Chem. Rev. 2020, 120, 4831–4847. 10.1021/acs.chemrev.9b00557. 31797671

[ref472] Frank F. C. On Spontaneous Symmetric Synthesis. Biochim. Biophys. Acta 1953, 11, 459–63. 10.1016/0006-3002(53)90082-1. 13105666

[ref473] Howlett M. G.; Fletcher S. P. From Autocatalysis to Survival of the Fittest in Self-reproducing Lipid Systems. Nat. Rev. Chem. 2023, 7, 1–19. 10.1038/s41570-023-00524-8. 37612460

[ref474] Guijarro A.; Yus M. The Origin of Chirality in the Molecules of Life: a Revision from Awareness to the Current Theories and Perspectives of this Unsolved Problem; RSC Publishing: 2008.

[ref475] Black R.; Cohen Z.; Todd Z.; Maibaum L.; Catling D., Stabilization of Prebiotic Vesicles by Peptides Depends on Sequence and Chirality. Research Square 2023, 10.21203/rs.3.rs-3136920/v1 38629792

[ref476] Nutman A. P.; Bennett V. C.; Friend C. R.; Van Kranendonk M. J.; Chivas A. R. Rapid Emergence of Life Shown by Discovery of 3,700-million-year-old Microbial Structures. Nature 2016, 537, 535–538. 10.1038/nature19355. 27580034

[ref477] Benyus J. M. Biomimicry: Innovation Inspired by Nature, 1st ed.; Morrow New York: New York, 1997.

[ref478] Zhang W.; Li J.; Lu G.; Guan H.; Hao L. Enantiomer-selective Sensing and the Light Response of Chiral Molecules Coated with a Persistent Luminescent Material. Chem. Commun. 2019, 55, 13390–13393. 10.1039/C9CC06014F. 31637379

[ref479] Ziv A.; Shoseyov O.; Karadan P.; Bloom B. P.; Goldring S.; Metzger T.; Yochelis S.; Waldeck D. H.; Yerushalmi R.; Paltiel Y. Chirality Nanosensor with Direct Electric Readout by Coupling of Nanofloret Localized Plasmons with Electronic Transport. Nano Lett. 2021, 21, 6496–6503. 10.1021/acs.nanolett.1c01539. 34297582

[ref480] Cavin R. K.; Lugli P.; Zhirnov V. V. Science and Engineering Beyond Moore’s Law. Proc, IEEE 2012, 100, 1720–1749. 10.1109/JPROC.2012.2190155.

